# Comparative Proteomic Analysis of *Histoplasma capsulatum* Yeast and Mycelium Reveals Differential Metabolic Shifts and Cell Wall Remodeling Processes in the Different Morphotypes

**DOI:** 10.3389/fmicb.2021.640931

**Published:** 2021-06-11

**Authors:** Marcos Abreu Almeida, Lilian Cristiane Baeza, Rodrigo Almeida-Paes, Alexandre Melo Bailão, Clayton Luiz Borges, Allan Jefferson Guimarães, Célia Maria Almeida Soares, Rosely Maria Zancopé-Oliveira

**Affiliations:** ^1^Instituto Nacional de Infectologia Evandro Chagas, Fundação Oswaldo Cruz, Rio de Janeiro, Brazil; ^2^Centro de Ciências Médicas e Farmacêuticas, Universidade Estadual do Oeste do Paraná, Cascavel, Brazil; ^3^Instituto de Ciências Biológicas, Universidade Federal de Goiás, Goiânia, Brazil; ^4^Departamento de Microbiologia e Parasitologia, Universidade Federal Fluminense, Niterói, Brazil

**Keywords:** *Histoplasma capsulatum*, dimorphism, yeast, mycelium, proteomic analysis, fungal biology

## Abstract

*Histoplasma capsulatum* is a thermally dimorphic fungus distributed worldwide, but with the highest incidence in the Americas within specific geographic areas, such as the Mississippi River Valley and regions in Latin America. This fungus is the etiologic agent of histoplasmosis, an important life-threatening systemic mycosis. Dimorphism is an important feature for fungal survival in different environments and is related to the virulence of *H. capsulatum*, and essential to the establishment of infection. Proteomic profiles have made important contributions to the knowledge of metabolism and pathogenicity in several biological models. However, *H. capsulatum* proteome studies have been underexplored. In the present study, we report the first proteomic comparison between the mycelium and the yeast cells of *H. capsulatum*. Liquid chromatography coupled to mass spectrometry was used to evaluate the proteomic profile of the two phases of *H. capsulatum* growth, mycelium, and yeast. In summary, 214 and 225 proteins were only detected/or preferentially abundant in mycelium or yeast cells, respectively. In mycelium, enzymes related to the glycolytic pathway and to the alcoholic fermentation occurred in greater abundance, suggesting a higher use of anaerobic pathways for energy production. In yeast cells, proteins related to the tricarboxylic acid cycle and response to temperature stress were in high abundance. Proteins related to oxidative stress response or involved with cell wall metabolism were identified with differential abundance in both conditions. Proteomic data validation was performed by enzymatic activity determination, Western blot assays, or immunofluorescence microscopy. These experiments corroborated, directly or indirectly, the abundance of isocitrate lyase, 2-methylcitrate synthase, catalase B, and mannosyl-oligosaccharide-1,2-alpha-mannosidase in the mycelium and heat shock protein (HSP) 30, HSP60, glucosamine-fructose-6-phosphate aminotransferase, glucosamine-6-phosphate deaminase, and *N*-acetylglucosamine-phosphate mutase in yeast cells. The proteomic profile-associated functional classification analyses of proteins provided new and interesting information regarding the differences in metabolism between the two distinct growth forms of *H. capsulatum*.

## Introduction

The thermally dimorphic fungus *Histoplasma capsulatum* is the etiological agent of histoplasmosis, an important systemic mycosis with a worldwide distribution that is endemic to areas of the United States of America, especially in the Ohio and Mississippi River Valleys, and most Latin American countries (Armstrong et al., 2018; Sahaza et al., 2020). Histoplasmosis causes a wide spectrum of clinical forms, from an asymptomatic, self-limited illness to a progressive disseminated and often fatal disease. The prognosis of infection depends fundamentally on three factors: the host immune response, the inhaled fungal inoculum, and the specific virulence characteristics of the infecting strain (Deepe and Buesing, 2012). *Histoplasma capsulatum* grows as a hyphal form in the environment at room temperature. Infection is initiated upon host inhalation of fungal propagules (conidia and/or hyphal fragments), which are then taken up by macrophages and other phagocytic cells. Once inside the mammalian host, the filamentous phase quickly differentiates into the yeast form, which can evade phagocytic killing and is able to replicate in phagosome milieu (Woods, 2003).

The morphological shift of *H. capsulatum* from mycelium to yeast cells, or vice versa, can also be reproduced *in vitro* by switching the fungal growth temperature from 25 to 37°C or the other direction (Maresca et al., 1994). *Histoplasma* differentiation into yeast-like cells is required for virulence, since *Histoplasma* strains genetically or chemically prevented from transitioning into yeasts are attenuated (Medoff et al., 1986; Nguyen and Sil, 2008). Several genes have been reported to play roles in phase transition regulation, such as the hybrid histidine kinase (Drk1) and four transcriptional regulators (Ryp1, Ryp2, Ryp3, and Ryp4) (Nemecek et al., 2006; Nguyen and Sil, 2008; Webster and Sil, 2008; Beyhan et al., 2013). Edwards et al. (2013) proposed that many genes specifically expressed by the yeast phase worked as virulence factors and greatly contributed to *Histoplasma* pathogenesis. Nonetheless, studies on the characterizations of several biological aspects of *H. capsulatum* continue to be applied to mycelium and/or yeast cells grown in the laboratory in order to identify genes and their products connected to virulence and explore their role during pathogenesis (Edwards and Rappleye, 2011; Mihu and Nosanchuk, 2012; Woods, 2016).

There are several *H. capsulatum* cell surface components identified that are related to virulence/pathogenicity: α-1,3-glucan synthase (Edwards et al., 2011), Yps3 (Bohse and Woods, 2007), Hsp60 (Habich et al., 2006), Histone-2B (Nosanchuk et al., 2003), and melanin (Nosanchuk et al., 2002). Additionally, iron acquisition by *H. capsulatum* (Hwang et al., 2008; Hilty et al., 2011) and several fungal secretory proteins (Deepe and Gibbons, 2001; Guimarães et al., 2008; Edwards and Rappleye, 2011) are also associated with pathogenesis.

Fungal cell walls are dynamic and essential structures for cell viability, morphogenesis, and pathogenesis. The cell wall composition greatly influences the fungus’ ecology, and this composition is highly regulated in response to environmental conditions and imposed stress conditions (Gow et al., 2017). Glucose, mannose, and galactose are the most abundant monosaccharides found in the *H. capsulatum* cell wall under both morphologies. The saccharide concentrations vary according to the culture medium composition, environmental conditions, and the extraction method used (Domer et al., 1967; Kanetsuna et al., 1974). Glucans are glucose polymers that have a glycoside bond that differs in mycelium and yeast cells. The α- and β-glucans present in the cell walls of these morphological forms have different biological roles. While β-1,3-glucan is an antigenic molecule and can be recognized by innate immunity cell through dectin-1 and is involved in the modulation of the host immune response (Gorocica et al., 2009), the α-1,3-glucan present on yeasts can inhibit the recognition of the fungal cell and facilitate fungal immune escape (Rappleye et al., 2007).

Despite the importance of morphology transition in *H. capsulatum*, there is a lack of proteomic quantitative comparisons between mycelium and yeast cells of this fungus. Thus, in this present study, proteomic strategies were used in order to identify molecular differences between the two *H. capsulatum* morphologies. We believe that the obtained data will be helpful in the determination of metabolic and molecular aspects preferentially used by mycelial and yeast cells, and the data provide new information that is helpful for understanding facets of the morphological transition.

## Materials and Methods

### Microorganism and Culture Conditions

*Histoplasma capsulatum* G217B (ATCC 26032) was used throughout this study. The cells were cultured in Ham’s F12 medium supplemented with cystine (8.4 mg/L), HEPES (6 g/L), glutamic acid (1 g/L), and glucose (18.2 g/L), pH 7.5, at 36°C and 25°C, to yield yeast and mycelial phases, respectively.

### Preparation of Protein Extracts

Cultures were carried out in 50 ml of Ham’s F12 medium, with an initial density of 1 × 10^8^cells/ml, under agitation (150x rpm), at 36°C (72 h) or 25°C (96 h), for the yeast and mycelial phases, respectively. After the incubation times, cells were harvested by centrifugation at 10,000 × *g* for 15 min at 4°C, followed by three washes with phosphate-buffered saline (PBS; 1.4 mM KH_2_PO_4_, 8 mM Na_2_HPO_4_, 140 mM NaCl, 2.7 mM KCl; pH 7.2). Then, the cells were suspended in extraction buffer (20 mM Tris-HCl pH 8.8; 2 mM CaCl_2_) containing a mixture of nuclease and protease inhibitors (GE Healthcare, Uppsala, Sweden). This mixture was distributed in tubes containing an equal volume of cell pellet and glass beads (0.5 mm, BioSpec Products), and the suspension was processed in a bead beater (BioSpec, Bartlesville, OK, United States) apparatus with five cycles of 30 s, with intervals of 1 min on ice. The supernatant was collected by centrifugation at 10,000 × *g* for 15 min at 4°C, and the protein concentrations were determined by Bradford assay using bovine serum albumin (BSA, Sigma-Aldrich Steinheim, Germany) as a standard (Bradford, 1976). The profile of the protein extracts (20 μg) obtained was evaluated by sodium dodecyl sulfate-polyacrylamide gel electrophoresis (SDS-PAGE) on 12% polyacrylamide gels (Laemmli, 1970).

### Digestion of Protein Extracts for Mass Spectrometry

Protein enzymatic digestion was processed according to the protocol described by Murad et al. (2011). Of the protein from each sample, 150 μg was added to 50 mM ammonium bicarbonate buffer, pH 8.5, and RapiGESTTM SF (0.2% v/v) (Waters, Milford, MA, United States), and incubated at 80°C for 15 min. Disulfide bonds were reduced by incubation with 100 mM dithiothreitol (DTT) (GE Healthcare, Piscataway, NJ, United States) at 60°C for 30 min, and then alkylation was performed by incubation with 300 mM iodoacetamide (GE Healthcare, Piscataway, NJ, United States) at room temperature in the dark for 30 min. Trypsin digestion was performed overnight at 37°C through the addition of 30 μl of a 0.05 -μg/μl trypsin solution (Promega, Madison, WI, United States). Digestion was stopped with the addition of 30 μl of 5% trifluoroacetic acid (v/v), followed by incubation at 37°C for 90 min. The samples were centrifuged, and supernatants were dried in a speed vacuum. The peptides were suspended in a solution containing 20 mM ammonium formate and 150 fmol/μl of Rabbit Phosphorylase B (PHB; Waters, Manchester, United Kingdom) as an internal standard.

### Proteomic Analysis by NanoUPLC-MS^*E*^

The tryptic peptides were separated by Ultra Performance Liquid Chromatography according to Pigosso et al. (2017). Peptides from each sample (1,660 ng) were injected to the nanoACQUITYTM UPLC system (Waters, Manchester, United Kingdom). The first dimension chromatography included a 5-μm NanoEaseTM BEH130 C18, 300 μm × 50 mm column (Waters, Milford, MA, United States). The bound peptides were separated into five fractions eluting at 11.4, 14.7, 17.4, 25.7, and 50% (v/v) acetonitrile/0.1% (v/v) formic acid gradient with a flow rate of 2,000 μl/min. In-line, eluted fractions were trapped in 5-μm Symmetry C18, 180 μm × 20 mm column (Waters, Milford, MA, United States). Second, dimension chromatography was carried out with a 1.7-μm NanoEaseTM BEH130 C18, 100 μm × 100 mm analytical column (Waters, Milford, MA, United States). All analyses were performed with nanoelectrospray ionization in the positive ion mode nanoESI(+) with a NanoLockSpray source. The double charged [(M + 2H)2 + = 785.8426] precursor ion [Glu]1-Fibrinopeptide B (GluFib) (Sigma, St. Louis, United States) at 200 fmol/μl solution was delivered through the reference sprayer of the NanoLockSpray source, and the MS/MS fragment ions of GluFib were used to obtain the final instrument calibration. Data-independent scanning (MSE) experiments were performed with a SynaptTM G1 HDMSTM System mass spectrometer (Waters, Manchester, United Kingdom) with a hybrid quadrupole/time-of-flight. The radiofrequency applied to the quadrupole mass analyzer was adjusted, such that the ions from m/z 50 to 2,000 were efficiently transmitted. Moreover, the spectrometer was automatically programmed to switch between low collision energy MS (3 eV) and elevated collision energies MS^*E*^ (12–40 eV). The transfer collision cell was adjusted to 1 eV with a scan time of 1.0 s, both in low and high energies, to give a minimum of 10 points for both conditions above 10% of peak capacity. The time-of-flight (TOF) analyzer was operated in mode “V” reflection. Runs were performed in triplicates for each sample.

### Raw Data Processing and Analysis

Raw files were analyzed together using the ProteinLynx Global Server software version 3.0.2 (PLGS) (Waters, Manchester, United Kingdom). The protein identifications and quantitative packaging were generated using specific algorithms (Silva et al., 2005, 2006), and search was performed against an *H. capsulatum*-specific database^1^. The ProteinLynx Global server v.3.0.2 (PLGS) with Expression E informatics v.3.0.2 was used for proper spectral processing, database searching conditions, and quantitative comparisons. The mathematic model used to calculate the ratios is part of the expression algorithm inside the PLGS from Waters Corporation (Geromanos et al., 2009). The identified proteins are organized by the expression algorithm into a statistically significant list corresponding to induced and reduced regulation ratios between yeast and mycelium phases. The software shows the expression analysis statistics as the induced proteins with a probability of upregulation of 0.95 or more and also the reduced proteins with a probability of 0.05 or less. A value of 1.00 indicates that the cluster is definitely upregulated; a value of 0.00 indicates that the cluster is definitively downregulated. PLGS uses the following strategy to peptide identification: first, only completely cleaved tryptic peptides are used for identification (PepFrag1). The second pass of the database algorithm (PepFrag2) is designed to identify peptide modifications and non-specific cleavage products to proteins that were positively identified in the first pass. The parameters for protein identification were as follows: (i) the detection of at least two fragment ions per peptide, (ii) five fragments per protein, (iii) the determination of at least one peptide per protein, (iv) maximum protein mass (600 kDa), (v) one missed cleavage site was allowed for trypsin, (vi) carbamidomethylation of cysteine was a fixed modification, (vii) methionine oxidation and phosphoryl STY as a variable modification, (viii) and a maximum 4% false-positive discovery rate, in at least two out of three technical replicate injections. Correct and reversed sequences databases were used to estimate false-positive rates (FPR). Proteins identified with low accuracy were excluded; on the other hand, proteins with one or more peptides were admitted, in at least two replicates. For the analysis of protein quantification levels, the observed intensity measurements were normalized with a protein that showed a variance coefficient of 0.05 and that was detected in all replicates (accession number: HCAG_07031). A 50%-fold change (Baeza et al., 2017) was used as a cutoff to determine the differentially abundant proteins in the yeast and mycelial forms of the fungus. The mass spectrometry proteomics data have been deposited in the PRIDE partner repository for the ProteomeXchange Consortium with the data set identifier: PXD022623. Proteins were functionally classified using Uniprot^2^ and KEGG^3^ databases. The annotation of non-characterized proteins was performed by homology from proteins present in the NCBI^4^. Experiment dynamic range, the peptide parts per million error (ppm) and peptide detection type using the softwares such as MassPivot v1.0.1. and Spotfire v8.0. Microsoft Excel 2013 (Microsoft, United States) was also used for table manipulations. Heat maps generated by the MultiExperiment Viewer software V.4.9^5^. Thereafter, the proteomic data were further validated through the experiments described below.

### Ethanol Quantification Assay

Protein extracts were obtained from 0.5 g of normalized dry weight cells from each morphology as described above. The ethanol concentrations in extracts were determined using an enzymatic detection kit according to the manufacturer’s instructions (UV-test for ethanol, RBiopharm, Darmstadt, Germany). The ethanol quantification was performed in triplicate.

### Western Blot Analysis

Proteins from yeast and mycelial phases of *H. capsulatum* were separated by SDS-PAGE, as described above, and transferred to 0.2-μm nitrocellulose membranes (Bio-Rad, Germany). The membranes were blocked at room temperature for 1 h PBS containing 5% (w/v) non-fat skim milk and supplemented with 0.2% Tween 20 (pH 7.2) (T-PBS). Then, the membranes were washed three times with T-PBS (5 min per wash). Next, the membranes were incubated overnight at 4°C with antibodies diluted in T-PBS: polyclonal antibody anti-HSP30 [1/500 (v/v)] (protein molecular mass 27.6 kDa), monoclonal antibody (mAb) anti-HSP60 [1/1,000 (v/v)] (protein molecular mass 61.8 kDa), or mAb anti-catalase [1/100 (v/v)] (protein molecular mass 57.3 kDa). A commercial polyclonal antibody (Abcam Plc, Cambridge, United Kingdom) anti-β-tubulin [1.0 mg/ml] [1/5,000 (v/v)] (molecular mass of 55.0 kDa), was used as a loading control. This step was followed by three washes of 5 min in T-PBS. The membranes were incubated with the appropriate conjugated secondary antibody, either a horseradish-peroxidase conjugated anti-rabbit IgG (Jackson ImmunoResearch, United States) or anti-mouse IgG (Jackson ImmunoResearch, United States), at 0.16 μg/ml. Finally, the Western blots were developed with SuperSignal West Dura Chemiluminescent substrate (Pierce, Rockford, IL, United States). The X-ray films were exposed and developed according to the manufacturer’s instructions (Kodak, Rochester, NY, United States).

### Isocitrate Lyase Activity

In this assay, 50 μg of protein extracts from mycelial and yeast cells were used. The isocitrate lyase activity was determined by measuring the glyoxylate formation as its phenylhydrazone derivative as described (Ebel et al., 2006). The product formation was followed by measuring the absorbance at 324 nm using an extinction coefficient of 16.8 mM^–1^ cm^–1^ in a reaction mixture containing 2 mM threo-D,L-isocitrate (Sigma Aldrich), 2 mM MgCl_2_, 10 mM phenylhydrazine HCl (Sigma Aldrich), 2 mM dithiothreitol, and 50 mM potassium phosphate at pH 7.0. Specific activity was determined as the amount of enzyme required to form 1 μmol of glyoxylate-phenylhydrazone per min per mg of total protein. Enzyme activity was performed in three biologically independent replicates.

### Methylcitrate Synthase Activity Measurement

The determination of methylcitrate synthase activity is based on the nitrothiophenolate (2-mercapto-5-nitrobenzoate dianion) formation during the reaction of 5,5’-ditiobis-(2-nitrobenzoate) (DTNB) with CoASH, which is released during the condensation of oxaloacetate with propionyl-CoA, as described (Brock and Buckel, 2004). A standard curve of DTT (Dithiothreitol), using concentrations ranging from 1 to 25 mM, was used as a control, as it exerted an analogous function to the CoASH group during reaction with 1 mM DTNB. The reaction final volume was adjusted to 150 μl with 50 mM Tris-HCl pH 8.0 and spectrophotometric measurements performed at 412 nm. For the assay, 5 μg of either crude protein extract was used, 1 mM DTNB, 0.2 mM propionyl-CoA, and the final reaction volume adjusted to 150 μl with Tris-HCl pH 8.0 at 50 mM. The assay was started by the addition of 1 mM oxaloacetate and monitored at 412 nm. The experiment was performed in triplicate. One unit of activity (U) was defined as the release of 1 μmol CoASH per milligram of protein per minute per ml.

### Immunofluorescence Microscopy

Differences in the cell wall constitutions of both phases of *H. capsulatum* were evaluated by determining the binding profiles of lectins such as concanavalin A (ConA) against mannosylated residues, wheat germ agglutinin (WGA) to chitoligomers, and Dectin-Fc against β-1,3-glucan by fluorescence microscopy. Uvitex 2B fluorescent dye was used for determining total chitin in fungal cell walls. Briefly, after the cultivation of yeast and mycelial phase cells of *H. capsulatum* as described previously, cells were washed three times in PBS, and 10^6^ cells were fixed with 4% paraformaldehyde overnight at –20°C. After, the cells were blocked with 1% BSA in PBS for 1 h. Then the analyses were carried out in two different systems: (i) Cells were incubated with Con A-FITC at a final concentration of 10 μg/ml and WGA-Alexa 546 at a final concentration of 1 μg/ml for 1 h, in an orbital shaker, in the dark at room temperature. (ii) Cells were incubated with Dectin-Fc at a final concentration of 5 μg/ml and WGA-Alexa 546 at a final concentration of 1 μg/ml for 1 h, under shaking, in the dark, at room temperature. A subsequent incubation with 2 μg/ml of goat anti-mouse IgG-Alexa 488 for 1 h was performed. Chitin in the cell wall was visualized by incubating the cells with a 5-mg/ml solution of Uvitex 2B in PBS for 15 min, at room temperature. Cells were washed three times with PBS between incubations. The samples were visualized using a fluorescence microscope (Carl Zeiss MicroImaging, Inc.), with a magnification of 100X. The fluorescence intensity of Images was analyzed by Image J (NIH, Bethesda, MD, United States) and edited with Adobe Photoshop C5S (Adobe Systems Software). A minimum of 100 cells were measured and total intensities compared between yeast and mycelium phases.

### Statistical Analyses

Statistical analyzes were performed using the GraphPad Prism 7.0. The Student’s *t*-test was employed, and *p* ≤ 0.05 was considered significant.

## Results

### Protein Extracts

The electrophoretic profiles of the proteins extracts from the yeast and mycelium forms of *H. capsulatum* were qualitatively evaluated through SDS-PAGE. For both samples, proteins displayed molecular weights above 30 kDa (Supplementary Figure 1).

### Evaluation of the Proteomic Data

From the analyses of the mycelium and yeast extracts by the nanoUPLC-MS^*E*^, 54 and 57% of the peptides were obtained upon the first step of protein fragmentation, respectively, whereas 10% occurred in the second step for both. Totals of 14 and 13% of the total peptides were identified by the lost trypsin cleavage, respectively, whereas the fragmentation rate that occurred at the ionization source was 12% for both conditions (Supplementary Figure 2). False-positive rates were 0.77 and 1.12%, respectively. These data represented good quality, since identifications by PepFrag 1 and 2 correspond to more than 50%, whereas in source fragmentation and lost cleavages, the comprised values were lower than 20% each. Most of the peptides generated by the two samples (>90%) were detected within a 10-ppm error (Supplementary Figure 3). The dynamic range of peptide detection analysis (Supplementary Figure 4) indicated distribution of three orders of magnitude, and demonstrating high and low abundance proteins levels. The dynamic detection range data for both samples are in accordance with the literature, as the sigmoid pattern curve and the spiked standard of rabbit phosphorylase B (PHB) were displayed in a similar way within the two samples.

### Differentially Abundant Proteins on Mycelial and Yeast *H. capsulatum* Morphologies

**FIGURE 1:**
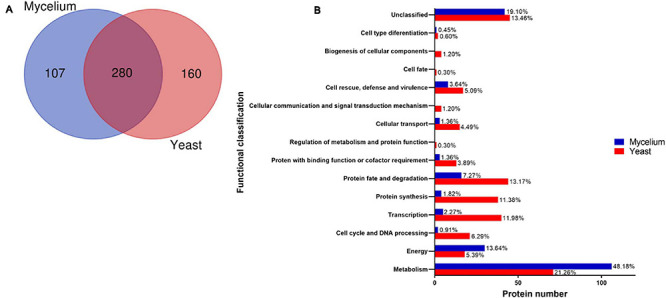
Proteome profile of *Histoplasma capsulatum* mycelium and yeast forms. **(A)** Venn diagram presenting the total number of proteins identified in extracts from both *H. capsulatum* morphologies (http://bioinformatics.psb.ugent.be/webtools/Venn/); **(B)** Functional classification of *H. capsulatum* proteins obtained by NanoUPLC-MS^*E*^ analysis identified with preferential abundance in mycelium and yeast forms. The biological processes of the differentially expressed proteins in the isolates were obtained using the Uniprot (http://www.uniprot.org) and KEGG: Kyoto Encyclopedia of Genes and Genomes (www.genome.jp/kegg).

Proteins (387) were identified in the mycelial form extracts (Supplementary Table 1) in comparison with 440 proteins in the yeast form (Supplementary Table 2). Proteins (280) were commonly present in extracts of both morphologies of *H. capsulatum* (Figure 1A). Two hundred fourteen were more abundant in mycelium, and 335 were more abundant in *H. capsulatum* yeast cells (*p* < 0.05). The exclusively detected or differentially abundant proteins within each extract were categorized and grouped in accordance with the Functional Catalog (FunCat2), accessed in the MIPS database (Ruepp et al., 2004). The functional classification of proteins within both extracts is described in Supplementary Tables 3, 4. Proteins related to metabolism are the most abundant in both yeast (21.3%) and mycelium (48.2%) forms. In the yeast phase, the next most prevalent protein types belonged to protein fate and degradation (13.2%), transcription (12.0%), and protein synthesis (11.4%) categories. For the mycelial morphotype after metabolism, the most common proteins belonged to energy (13.6%) and protein fate and degradation (7.3%) categories. Less than 20% of the analyzed proteins were not classified or had unknown functions (Figure 1B).

### Glycolysis and Fermentation Are Favored Pathways in Mycelial *H. capsulatum* Cells

**FIGURE 2:**
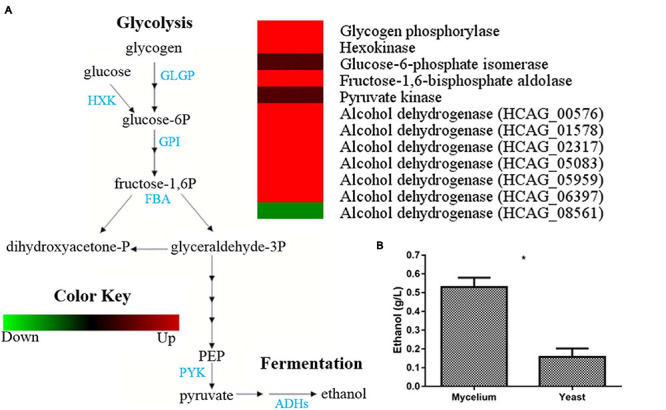
Comparison of protein profiles related to glycolysis and fermentation in *H. capsulatum* mycelium and yeast forms. A 50%-fold change was used as a cutoff to determine the differentially abundant proteins in the fungus yeast and mycelial forms. The Multi Experiment Viewer software V.4.9 was used to group and compare the abundance data. **(A)** The diagram representing the glycolytic and fermentation metabolic pathways represents differentially abundant proteins in the mycelium and yeast phases. Changes in the abundance levels in the mycelium compared with the yeast are represented in the heat map. Experimental triplicate mean values are presented for the lowest abundance (green) and the *H. capsulatum* proteins highest abundance (red) in the mycelium form. Black indicates that no significant difference was observed. GLGP, glycogen phosphorylase; HXK, hexokinase; GPI, glucose-6-phosphate isomerase; FBA, fructose-1,6-bisphosphate aldolase; PYK, pyruvate kinase; ADHs, alcohol dehydrogenases. **(B)** Ethanol quantification assay. The ethanol concentration (g/L) was determined in *H. capsulatum* yeast and mycelial phase cells, upon growth in Hams F12 media for 72 h (36°C) and 96 h (25°C), respectively. The cells were disrupted in the bead beater, and the ethanol compound was quantified using an enzymatic detection kit (UV-test for ethanol, RBiopharm, Darmstadt, Germany). Data are expressed as the mean ± standard deviation of biological triplicates in independent experiments. *p < 0.05.

Glycolysis enzymes, such as hexokinase (HCAG_03661) and fructose-1,6-bisphosphate aldolase (HCAG_08789), were detected exclusively in mycelium. Besides detection in both morphologies, glucose-6-phosphate isomerase (HCAG_08202) and pyruvate kinase (HCAG_07781) were more abundant in the mycelium (Supplementary Table 3). Notably, the enzyme glycogen phosphorylase (HCAG_08447), which digests glycogen and provides glucose for glycolysis, accumulated in mycelium. Besides the detection of the fermentation enzyme alcohol dehydrogenases in both morphologies, six of them were exclusive at the mycelial extracts (HCAG_05959, HCAG_00576, HCAG_01578, HCAG_02317, HCAG_05083, and HCAG_06397), whereas only HCAG_08561 was more abundant in yeast. The comparison of abundance levels of enzymes related to glycolysis and fermentation in the mycelium and yeast forms of *H. capsulatum* are presented in Figure 2A.

In order to validate whether, in fact, fermentation occurs preferably in *H. capsulatum* mycelia, the capacity for ethanol production of both extracts was compared. Upon incubations, ethanol concentrations were determined to be almost five times higher in the mycelial extract than in the yeast extract (*p* = 0.0006), confirming the higher capacity of *H. capsulatum* mycelial cells to obtain energy through fermentative metabolism than yeasts (Figure 2B).

### Pentose Phosphate Pathway and Stress Response Are Different in Mycelial and Yeast *H. capsulatum* Cells

**FIGURE 3:**
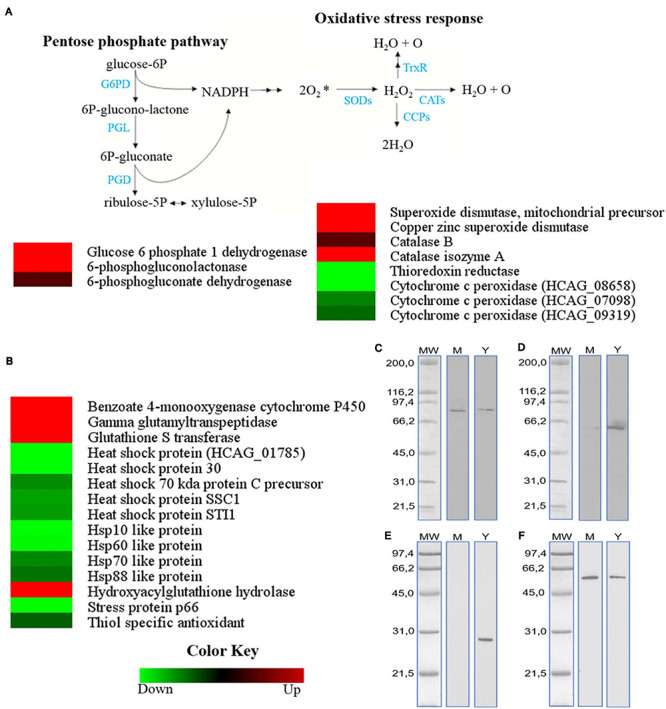
Comparison of protein profiles related to pentose phosphate pathway and stress response in *H. capsulatum* mycelium and yeast forms. Dataset comparisons were carried out as cited in the Figure 2 legend. **(A)** The diagram representing the pentose phosphate and oxidative stress response pathways represents differentially abundant proteins in the mycelium and yeast phases. **(B)** Comparison of protein profiles related to the stress response in mycelium and yeast forms of *H. capsulatum*. Changes in the abundance levels in the mycelium compared with the yeast are represented in the heat map. Experimental triplicate mean values are presented for the lowest (green) and the highest (red) abundance of *H. capsulatum* proteins in the mycelium form. Black indicates that no significant difference was observed. G6PD, glucose 6 phosphate 1 dehydrogenase; PGL, 6-phosphogluconolactonase; PGD, 6-phosphogluconate dehydrogenase; SODs, superoxide dismutases; TRxR, thioredoxin reductase; CATs, catalases; C, cytochrome *c* peroxidases. Confirmatory Western blot analysis of selected proteins detected in the *H. capsulatum* proteomic analyses. Protein extracts are shown after the reaction with antibodies against the following proteins: **(C)** catalase; **(D)** HSP60; **(E)** HSP30; **(F)** β-tubulin, as a loading control. The blots were incubated with goat anti-mouse IgG polyclonal or goat anti-rabbit IgG polyclonal antibodies coupled to peroxidase and developed with SuperSignal West Dura Chemiluminescent substrate (Pierce, Rockford, IL, United States). The X-ray films were developed using Kodak imaging films according to the manufacturer’s instructions.

Mass spectrometry detection of proteins suggests a preferential use of the pentose phosphate pathway by *H. capsulatum* mycelia (Figure 3A, left panel). The enzymes glucose-6-phosphate 1-dehydrogenase (HCAG_04329), 6-phosphogluconolactonase (HCAG_04762), and 6-phosphogluconate dehydrogenase (HCAG_05884) were more abundant in this morphology, indicating NADPH generation by mycelium. Since NADPH oxidases generate oxygen free radicals, we further evaluated the proteins involved in the oxidative stress response. Enzymes related to detoxification and response to oxidative stress were differentially expressed in either morphologies of *H. capsulatum*, as depicted in Figure 3A (right panel). Two superoxide dismutase (SODs, HCAG_00642, and HCAG_01543) and a catalase A (HCAG_05109) were identified exclusively in the mycelium proteome. In contrast, a catalase B (HCAG_08064), which was identified in both phases, was preferentially expressed in the mycelium form. A cytochrome *c* peroxidase (HCAG_08658) and thioredoxin reductase (HCAG_07019) were exclusively found in yeasts. Two other cytochrome *c* peroxidases (HCAG_07098 and HCAG_09319) were also detected in both phases, but more abundant in the yeast proteome. Other proteins related to the stress response were also differentially abundant in mycelial or yeast cells of *H. capsulatum* (Figure 3B). All heat shock proteins (HSPs) were exclusive or more abundant in yeast cells. The expression of catalase B, HSP60, and HSP30 in distinct phase extracts were further evaluated using a Western blot approach (Figures 3C–E, respectively), which demonstrated expression levels in agreement with the proteomic data. Figure 3F shows the β-tubulin control, with similar expression in both morphotypes.

### Beta-Oxidation, Methylcitrate, and Glyoxylate Cycles Occur Preferentially in *H. capsulatum* Mycelia

**FIGURE 4:**
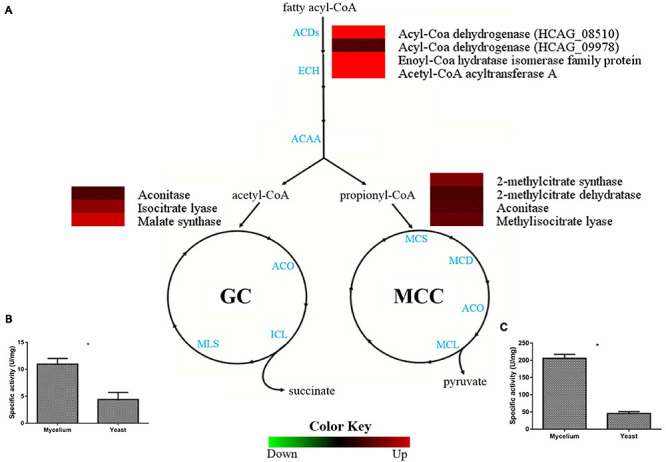
Comparison of protein profiles related to beta-oxidation, methylcitrate, and glyoxylate cycles in *H. capsulatum* mycelium and yeast forms. Dataset comparisons were carried out as cited in the Figure 2 legend. **(A)** The diagram representing the metabolic pathways of beta-oxidation, methylcitrate, and glyoxylate cycles represents differentially abundant proteins in the mycelium and yeast phases. Changes in the abundance levels in the mycelium compared with the yeast are represented in the heat map. Experimental triplicate mean values are presented for the lowest (green) and the highest (red) abundance of *H. capsulatum* proteins in the mycelium form. Black indicates that no significant difference was observed. ACDs, Acyl-Coa dehydrogenases; ECH, enoyl-Coa hydratase; ACAA, Acetyl-CoA acyltransferase; ACO, aconitase; ICL, isocitrate lyase; MLS, malate synthase; MCS, 2-methylcitrate synthase; MCD, 2-methylcitrate dehydratase; MCL, methylisocitrate lyase; **(B)** Isocitrate lyase (ICL) activity was determined by measuring glyoxylate formation as its phenylhydrazone derivative under the two conditions. The specific activity of ICL was determined as the amount of enzyme required to form 1 μmol of glyoxylate-phenylhydrazone per minute, per mg of total protein, and represented as U⋅mg-1. Errors bars represent standard deviation from three biological replicates. *p < 0.05. **(C)** Methylcitrate synthase measurement was determined in *H. capsulatum* yeast and mycelial phase cells, upon growth in Hams F12 media for 72 h (36°C) and 96°h (25°C), respectively. Activity was determined by nitrothiophenolate (2-mercapto-5-nitrobenzoate dianion) formation during the 5,5’-Dithiobis (2-nitrobenzoic acid) (DTNB) reaction with Coenzyme A, released during the condensation of oxaloacetate with propionyl-CoA.

Pathways involved in lipid utilization, such as beta-oxidation, methylcitrate, and glyoxylate cycles were more abundant in the *H. capsulatum* mycelial form, as demonstrated by proteomics (Figure 4A). The enzymes acetyl-CoA C-acyltransferase (HCAG_00780), enoyl-CoA hydratase (HCAG_02218), and acyl-CoA dehydrogenase (HCAG_08510) were identified exclusively in mycelia, and a second acyl-CoA dehydrogenase (HCAG_09978) was also more abundant in this form, indicating preferably lipid degradation and generation of acetyl-CoA and propionyl-CoA by this morphology. Regarding the glyoxalate cycle, the enzymatic activity of its key enzyme, an isocitrate lyase (HCAG_10962), was evaluated in both protein extracts, and a twofold increase (*p* = 0.0002) was detected in the mycelia (Figure 4B). A similar scenario was observed for enzymes related to the methylcitrate cycle, such as the methylcitrate synthase (HCAG_05090), an enzyme that catalyzes a key step of the pathway and, therefore, regulates cycle speed, whose activity was four times higher in the mycelial extract (*p* = 0.0001) (Figure 4C).

### Amino Acid Metabolism Is Active and Distinct in *H. capsulatum* Mycelia and Yeast Phases

**FIGURE 5:**
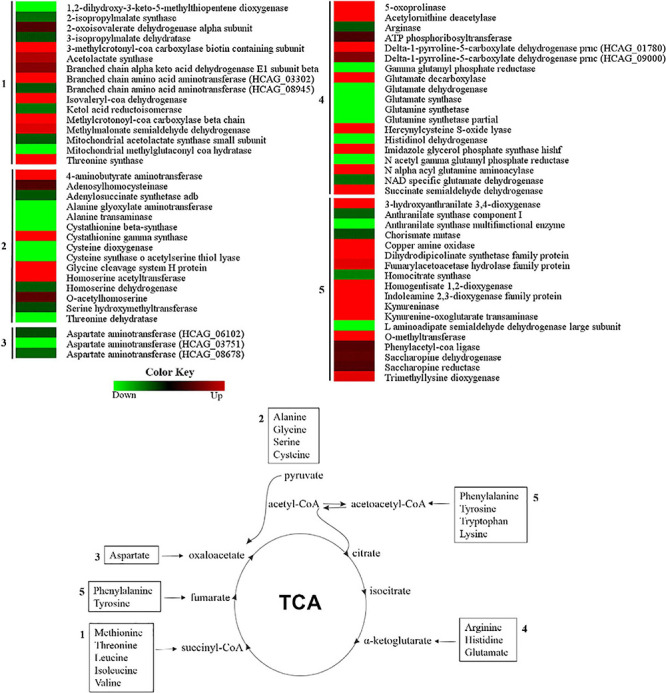
Comparison of protein profiles related to amino acid metabolism in *H. capsulatum* mycelium and yeast forms. Dataset comparisons were carried out as cited in the Figure 2 legend. Changes in the abundance levels in the mycelium compared with the yeast are represented in the heat map. Experimental triplicate mean values are presented for the lowest (green) and the highest (red) abundance of *H. capsulatum* proteins in the mycelium form. Black indicates that no significant difference was observed.

The main products generated by the amino acid group degradation and their fate on the tricarboxylic acid (TCA) cycle, as well as a heat map of the enzyme abundance levels in the mycelium extract, in comparison with the yeast extract are displayed in Figure 5. Seventy-two proteins in this category presented differential abundance among the two fungal morphologies. In general, proteins involved on the amino acid degradation that generate pyruvate precursors, such as alanine transaminase (HCAG_05679) and alanine-glyoxylate aminotransferase (HCAG_10952), were more abundant in yeast cells. Additionally, enzymes involved in the generation of oxaloacetate from aspartate, as the aspartyl transferases HCAG_03751, HCAG_06102, and HCAG_08678, and arginase (HCAG_00035), which generates α-ketoglutarate from arginine, were also more abundant in yeasts. In contrast, mycelial samples had a greater abundance of enzymes producing fumarate from tyrosine and phenylalanine, such as fumarylacetoacetase hydrolase (HCAG_02120) and homogentisate 1,2-dioxygenase (HCAG_05721), and those involved in the conversion of succinyl-CoA from branched amino acids, such as 2-oxoisovalerate dehydrogenase (HCAG_05761), methylmalonate-semialdehyde dehydrogenase (HCAG_06059), and branched-chain amino acid aminotransferase (HCAG_03302).

### Tricarboxylic Acid Cycle and Oxidative Phosphorylation Are More Active in *H. capsulatum* Yeast Cells

**FIGURE 6:**
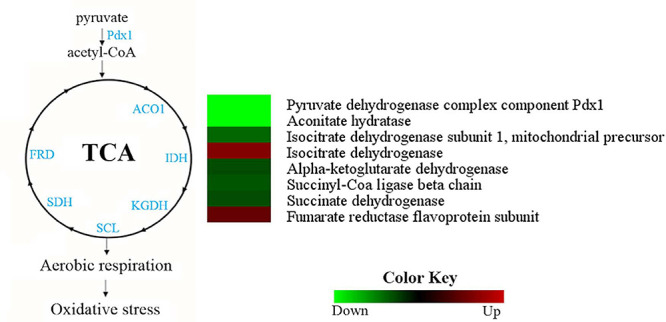
Comparison of protein profiles related to tricarboxylic acid (TCA) cycle and oxidative phosphorylation in *H. capsulatum* mycelium and yeast forms. Dataset comparisons were carried out as cited in the Figure 2 legend. The diagram representing the metabolic pathways of TCA cycle and oxidative phosphorylation represents differentially abundant proteins in the mycelium and yeast phases. Changes in the abundance levels in the mycelium compared with the yeast are represented in the heat map. Experimental triplicate mean values are presented for the lowest (green) and the highest (red) abundance of *H. capsulatum* proteins in the mycelium form. Black indicates that no significant difference was observed. Pdx1, pyruvate dehydrogenase; ACO1, aconitate hydratase; IDH, isocitrate dehydrogenase; KGDH, alpha-ketoglutarate dehydrogenase; SCL, succinyl-Coa ligase; SDH, succinate dehydrogenase; FRD, fumarate reductase.

Most TCA cycle enzymes were more abundant in the *H. capsulatum* yeast phase (Figure 6). A pyruvate dehydrogenase (HCAG_04294), which promotes the conversion of pyruvate into acetyl-CoA for the initiation of the TCA cycle, and aconitate hydratase (HCAG_05531) were exclusively found in the yeast proteome. Additionally, four other enzymes belonging to the TCA cycle were more abundant in the yeast form: alpha-ketoglutarate dehydrogenase (HCAG_01535), isocitrate dehydrogenase (HCAG_04093), succinate dehydrogenase (HCAG_06317), and succinyl-CoA ligase (HCAG_07697). In contrast, only two enzymes were predominant in mycelium: fumarate reductase (HCAG_03323) and isocitrate dehydrogenase (HCAG_04358). As for oxidative phosphorylation, proteins associated with the electron membrane transport and respiration were more abundant in yeast than the mycelial proteome (Supplementary Tables 3, 4).

### Differential Abundance of Enzymes Involved in the Cell Wall Synthesis Dictates Compositional Differences Between Mycelium and Yeast

**FIGURE 7:**
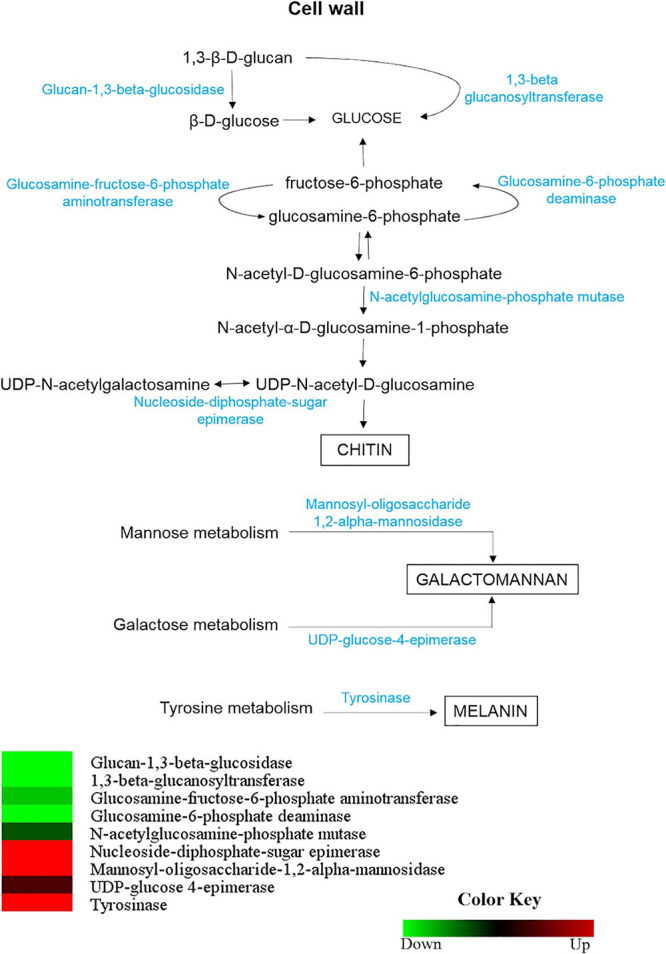
Comparison of protein profiles related to the metabolism of compounds present in the *H. capsulatum* mycelium and yeast forms the cell wall. Dataset comparisons were carried out as cited in the Figure 2 legend. Changes in the abundance levels in the mycelium compared with the yeast are represented in the heat map. Experimental triplicate mean values are presented for the lowest (green) and the highest (red) abundance of *H. capsulatum* proteins in the mycelium form. Black indicates that no significant difference was observed.

Enzymes involved in the synthesis and/or degradation of *H. capsulatum* cell with differential abundance in mycelium and yeast are shown in Figure 7. The enzymes glycan 1,3-beta glucosidase (HCAG_01828) and 1,3-beta glucanosyltransferase (HCAG_07210), which catalyze the conversion of β-1,3-glucan to glucose and the linkage of distinct β-1,3-glucan and polysaccharide fiber elongation, respectively, were identified exclusively in the yeast proteome. The high expression of both could lead to a rapid turnover and remodeling on the *H. capsulatum* cell wall.

Enzymes related to chitin production in *H. capsulatum*, such as glucosamine-fructose-6-phosphate aminotransferase (HCAG_04088), glucosamine-6-phosphate deaminase (HCAG_08152) and *N*-acetylglucosamine-phosphate mutase (HCAG_00064), were also more abundant in the yeast form. In contrast, the enzyme nucleoside-diphosphate-sugar epimerase, which catalyzes the conversion of UDP-*N*-acetylgalactosamine into UDP-*N*-acetyl-D-glucosamine, was preferentially abundant in the mycelium phase, indicating the use of this substrate in the synthesis of chitin by this morphology. Enzymes related to mannose metabolism, such as mannosyl-oligosaccharide 1,2-alpha-mannosidase (HCAG_08449), and galactose metabolism, UDP-glucose 4-epimerase (HCAG_09614), were also more abundant in the *H. capsulatum* mycelium.

**FIGURE 8:**
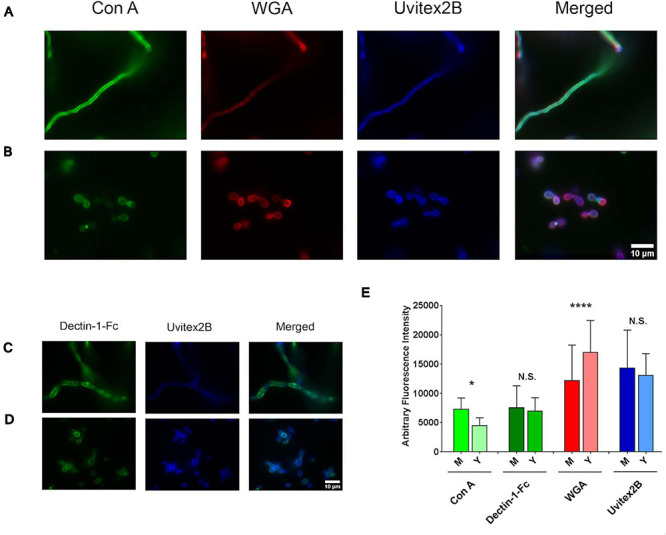
*Histoplasma capsulatum* cell wall composition from mycelium and yeast cells. Immunofluorescence microscopy showing the labeling pattern of *H. capsulatum* mycelium **(A**,**C)** and yeast cells **(B**,**D)** by Con A–FITC, WGA-Fc (Alexa 546), Uvitex 2B, and Dectin-Fc. **(E)** Arbitrary fluorescence intensity of markers presented in previous panels. N. S., not significant; *p < 0.05; ****p < 0.0001.

To validate the above findings, fluorescence microscopy targeting cell wall structures was performed with mycelium and yeast forms, and fluorescence levels were compared (Figure 8). Concanavalin A, which interacts with mannose residues, bound more intensely to the mycelial phase cell wall, displaying higher fluorescence intensity (*P* < 0.0001). WGA, a lectin with affinity for chitin oligomers, showed better binding to and higher fluorescence intensity with *H. capsulatum* yeast (*P* < 0.0001). Dectin-1-Fc, which recognizes β-1,3-glucan structures, did not produce statistically significant differences in binding when comparing mycelium and yeast fluorescence levels (*P* = 0.7725). Uvitex 2B was used to detect total cell wall chitin and delineate the cell wall dimensions, and fluorescence levels showed no difference when comparing both morphologies (*P* = 0.0549).

The enzyme tyrosinase (HCAG_04336), an enzyme directly related to the production of melanin, which is another important component of the *H. capsulatum* cell wall, was more abundant in the *H. capsulatum* mycelium form.

## Discussion

Dimorphism is one of the factors associated with fungal pathogenicity (Gauthier, 2017). The knowledge on the proteomic profile of both saprotrophic and parasitic forms of *H. capsulatum* is a way to understand its metabolism and to potentially reveal new strategies to be applied in the histoplasmosis management. This is the first study that has compared the proteomic profiles of the two morphologies of *H. capsulatum*. It demonstrates that proteins specifically expressed or differentially abundant in either phase are involved in distinct pathways, suggesting a metabolic change during dimorphism.

In our model, glycolysis and pyruvate generation occurs preferentially in mycelium, since pyruvate kinase was more abundant, and hexokinase was identified exclusively in the mycelial phase. An increase in enzymes involved in glycogen metabolism, such as glycogen phosphorylase and glucose-6-phosphate isomerase, was detected in mycelium, which could explain a glucose supply to the glycolytic pathway. As a result, there is an increase in glucose-6-phosphate (G6P) and fructose-6-phosphate (F6P) derivatives, which could be used in other metabolic pathways. The pentose phosphate pathway is linked to glycolysis at G6P and glyceraldehyde 3-phosphate (G3P) levels. The G6P resultin from hexokinase or glycogen phosphorylase activities can be used in the pentose phosphate pathway, since the first enzyme in the pathway (glucose-6-phosphate 1-dehydrogenase) was also detected only in mycelium, as well as 6-phosphogluconolactonase. Consequently, the pentose phosphate pathway in this morphological phase of *H. capsulatum* was also increased, unlike in *Candida albicans* and *Paracoccidioides lutzii* models, in which these pathways were increased in yeast cells (Monteoliva et al., 2011; Rezende et al., 2011).

In the comparative *H. capsulatum* proteome, some alcohol dehydrogenases were exclusively identified in mycelium suggesting that *H. capsulatum* mycelium is capable of utilizing glucose anaerobic metabolism. Higher ethanol concentrations in mycelium confirmed that the alcoholic fermentation occurs preferably in the *H. capsulatum* mycelial phase, which is similar to processes in *Paracoccidioides brasiliensis* (Vaz, 2014) and *C. albicans* (Monteoliva et al., 2011). These studies differ from the *P. lutzii* proteome, where alcoholic fermentation occurs preferentially in the yeast form (Rezende et al., 2011).

In fungi, HSPs respond to various stimuli, such as pH changes, oxidative stress, and increased temperatures (Burnie et al., 2006). Their adaptive expression in fungi indicates their importance in dimorphic pathogens (Cleare et al., 2017). Nine HSPs were identified in this study, all with greater abundance in or exclusive to the yeast form. HSP60 has been described as the main cell recognition ligand between *H. capsulatum* and CD11b/CD18 (CR3) in host macrophages (Long et al., 2003), highlighting its importance in fungal pathogenesis. Its protective role was also demonstrated through three different mouse strains in which vaccination with HSP60 provided protection against an intravenous lethal challenge with *H. capsulatum* yeasts (Gómez et al., 1991) and in a pulmonary histoplasmosis model (Gómez et al., 1995). Its importance as a target for passive immunotherapies was also described (Guimarães et al., 2009). *H. capsulatum* HSP60 interacts with other proteins, acts on dimorphism, heat stress, and pathogenesis of the fungus, as demonstrated by an analysis of the physical interaction network with this chaperone, which showed a possible involvement of this protein in the unfolding and protein translocation pathways (Guimarães et al., 2011a). Translocation of stress can also be performed with other chaperones’ help, such as HSP70. Similarities reported in the study by Guimarães et al. (2011a) are observed in studies that investigated the interactions between chaperones and other proteins in the *S. cerevisiae* model (Gong et al., 2009; Tapley et al., 2009, 2010). Both species showed HSP60 interaction with chaperonins HSP82 (HCAG_04686), SSA4 (HCAG_05805, HSP 70 kDa precursor), and SSE1 (HCAG_00783, HSP88 type protein). In addition, they also demonstrated another interaction in *S. cerevisiae* with a peptidyl-prolyl *cis-trans* isomerase (HCAG_08833). In our analysis, all the five proteins mentioned above were also more abundant in *H. capsulatum* yeast cells.

Some proteins involved in oxidative stress protection, such as superoxide dismutases, were uniquely identified in the *H. capsulatum* mycelium form. A cytosolic copper–zinc superoxide dismutase was also identified with greater abundance in the *Paracoccidioides* spp. mycelium (Tamayo et al., 2016). Catalases were also identified in the *H. capsulatum* proteome; catalase A was uniquely identified in mycelium and catalase B with greater abundance in the same morphotype. Our findings corroborate with the data of Johnson et al. (2002) that described the higher abundance of catalase A in the mycelium and catalase B expression in both morphologies (Guimarães et al., 2008).

Fatty acid catabolism is important for virulence and survival of pathogens (Muñoz-Elías et al., 2006). The comparative *H. capsulatum* proteomics results suggest that beta-oxidation, and the glyoxylate and methylcitrate cycles are preferentially induced in the mycelium, which is different from what occurs in the genus *Paracoccidioides* (Rezende et al., 2011; Vaz, 2014). In *Aspergillus fumigatus*, the methylcitrate synthase gene deletion led to virulence attenuation (Brock and Buckel, 2004), and in *Paracoccidioides* spp., the ability to metabolize propionyl-CoA is related to virulence (Santos et al., 2020). Further studies will be necessary to assess its role on *H. capsulatum*.

Amino acid catabolism, with the substrates produced for the TCA cycle, is an aspect of the *H. capsulatum* metabolism that differs between the two fungal morphologies. According to our proteomic data, both morphologies can differentially use amino acids as an energy source. Our results demonstrated a high number of enzymes related to the metabolism of alanine, arginine, aspartate, phenylalanine, isoleucine, leucine, tyrosine, tryptophan, and valine. This finding was unusual, since similar patterns were not observed in other fungal proteomes (Monteoliva et al., 2011; Rezende et al., 2011; Vaz, 2014). A hypothesis that would explain this observation is the differences in culture medium used for fungal growth, as we utilized Nutrient Mixture Ham’s F12. This medium contains the 20 essential amino acids, in concentrations ranging from 0.01 to 1 mM. The higher abundance of proteins related to protein fate and degradation in the fungus yeast form (Supplementary Tables 3, 4) suggests that amino acids are used as an energy source during parasitism. Other hypotheses for this observation include the differences in protein turnover or the need for new proteins in the mycelial *H. capsulatum* cells relative to yeasts.

Cysteine dioxygenase appears to play an important role in *H. capsulatum* dimorphism, a process where redox control and cysteine levels are crucial (Kumar et al., 1983). In the work by Kumar et al. (1983), this protein was purified from the cytosolic fraction of yeast cells and was present only in this *H. capsulatum* morphology, an observation our data confirmed. Interestingly, two other enzymes involved in cysteine metabolism have been detected only in the yeast phase, cystathionine beta-synthase and cysteine synthase *O*-acetylserine (thiol)-liase, suggesting a role of this amino acid in yeast maintenance.

The *H. capsulatum* yeast form seems to preferentially obtain pyruvate, oxaloacetate, and alpha-ketoglutarate for the TCA cycle by the degradation of the amino acids alanine, aspartate, and arginine, respectively. In our model, the key enzymes of the TCA cycle were more abundant in yeast cells, as occurs in *P. brasiliensis* (Vaz, 2014) and *Tallaromyces marneffei* (Pasricha et al., 2017). This is in agreement with a lower fermentative metabolism observed through ethanol dosage in *H. capsulatum* yeasts. In mycelium, the TCA cycle seems to be driven by the phenylalanine and tyrosine degradation in acetoacetyl-CoA and fumarate, tryptophan, and leucine in acetyl-CoA, and leucine, isoleucine, and valine to succinyl-CoA. In *C. albicans*, hyphal growth is associated with a decrease in the TCA cycle and an increase in ethanol production (Monteoliva et al., 2011). Since hyphae are one of the *C. albicans* parasitic structures, the fermentative metabolism occurs preferentially into the host during mycelial growth, as opposed to what probably occurs with *H. capsulatum* where the yeast phase predominates.

*Histoplasma capsulatum* cell wall composition changes during morphogenesis, which has biological implications (Guimarães et al., 2011b). Previous studies comparing *H. capsulatum* yeast and mycelium report that the yeast cell walls contain more chitin (Domer et al., 1967; Kanetsuna, 1981), which is in agreement with the WGA interaction results herein presented (Liedke et al., 2017). Moreover, enzymes related to chitin biosynthesis presented lower abundance in mycelium. The results also suggest that *H. capsulatum* mycelial cell walls present more mannose, since an enzyme related to the mannose metabolism was uniquely detected in this condition. These findings are corroborated with the microscopy data, which showed greater mycelial fluorescence after ConA incubation. This is also in agreement with previous studies that quantified mannose in the mycelium and yeast cell walls of *H. capsulatum* and *H. farciminosum*, and both had higher amounts of mannose in the mycelium (Domer et al., 1967; San-Blas and Carbonell, 1974).

Melanin protects *H. capsulatum* against several harsh conditions (Guimarães et al., 2011b). *H. capsulatum* produces melanin in both morphologies: mycelium melanizes under regular culture conditions, while the yeast form requires medium supplementation with phenolic compounds for *in vitro* melanization (Nosanchuk et al., 2002). We hypothesize that tyrosinase was not detected in the yeast proteome because the culture medium used in this study was not supplemented with phenolic compounds. Hwang et al. (2003) identified a differential expression of tyrosinase, a regulator of melanin production in mycelium. This enzyme was also detected in the mycelium proteome herein described, which would support conidial melanization (Nosanchuk et al., 2002).

Pyomelanin is a water-soluble pigment produced from the homogentisate excess obtained during L-tyrosine catabolism, which is oxidized to benzoquinone acetate and then polymerized. In *H. capsulatum*, only yeasts produce pyomelanin after growth in the presence of L-tyrosine (Almeida-Paes et al., 2018). The absence of pyomelanin in the mycelium phase observed in our previous study could be explained by our current results as this phase preferentially express two enzymes (homogentisate 1,2 dioxigenase and fumarylacetoacetate hydrolase) that participate in the homogentisate degradation, the pyomelanin precursor.

The protein disulfide isomerase was identified with preferential abundance in *H. capsulatum* yeast cells. This protein was identified during the dimorphism of *P. lutzii* and *Ustilago maydis* (Böhmer et al., 2007; Rezende et al., 2011). This protein could also be associated with the *H. capsulatum* dimorphic transition. Another hypothetical role for this enzyme is the interaction with YPS3, a protein located in the *H. capsulatum* cell wall that has homology with the BAD1 adhesin of *B. dermatitidis* (Bohse and Woods, 2005).

## Conclusion

Considering the proteomic differences found in the two *H. capsulatum* morphologies, we can conclude that abundant proteins in the mycelial form without human homologs, such as saccharopine dehydrogenase (HCAG_01145) or the Y20 protein (HCAG_04745), may be future targets for the development of new prophylactic approaches to combat the development of histoplasmosis by inhibiting the dimorphic transition to the pathogenic yeast phase. Similarly, the preferential pathways in yeast cells, such as cell wall remodeling, and enzymes from amino acid metabolism not present in mammalian cells, such as anthranilate synthase (HCAG_05224 and HCAG_00748), chorismate mutase (HCAG_00290), and histidinol dehydrogenase (HCAG_02357), may be candidates as targets for the development of new antifungal drugs. Additionally, proteins present in the yeast form can be used to develop diagnostics, particularly through antibody-based methodologies (Almeida et al., 2020). In sum, our findings reveal that proteomic analyses of the dimorphic pathogen can provide important insights into disease pathogenesis and lead to potential targets that can change future clinical practice.

## Data Availability Statement

The datasets presented in this study can be found in online repositories. The names of the repository/repositories and accession number(s) can be found below: https://www.ebi.ac.uk/pride/archive/, PXD022623.

## Author Contributions

MA and LB performed the experiments. MA, LB, RA-P, AB, CB, and AG designed the experiments, analyzed and interpreted the data, and wrote the manuscript. CS and RZ-O participated in the study design, data analysis, and revised the manuscript. All authors have contributed intellectually during the writing process, and have read and approved the final manuscript.

## Conflict of Interest

The authors declare that the research was conducted in the absence of any commercial or financial relationships that could be construed as a potential conflict of interest.

## Footnotes

1 http://www.ncbi.nlm.nih.gov/bioproject/20041

2 https://www.uniprot.org/proteomes/

3 https://www.genome.jp/kegg/pathway.html

4 https://www.ncbi.nlm.nih.gov/

5 mev.tm4.org/

## Supplementary Material

Supplementary Figure 1 SDS-PAGE (12% acrylamide) in Coomassie blue staining. MM, molecular mass; Y, yeast; M, mycelium. Numbers on the left correspond to the molecular mass pattern.

Supplementary Figure 2 Peptide detection type of *H. capsulatum* mycelia and yeast forms. PepFrag1 and Pepfrag2 – percentage of peptides compared to the *H. capsulatum* database in ProteinLynx Global Server version 2.4; VarMod, variable modifications; InSource, fragmentation occurred at the ionization source; MissedCleavage, missed cleavage performed by trypsin; and Neutral loss H_2_O and NH_3_ correspond to water and ammonia precursor losses. **(A,B)** Corresponds to yeast and mycelium forms, respectively.

Supplementary Figure 3 Experiment parts per million (ppm) error at the peptide level. The graphs indicate the identified peptides number in ppm. A total of 91.8 and 91.4% identified peptides were detected with an error of less than 10 ppm in yeast **(A)** and mycelium **(B)** forms.

Supplementary Figure 4 Detection dynamic range of proteomic analysis. Graphs for yeast **(A)** and mycelium **(B)** were generated, presenting proteins identified in a regular way (red), reverse (blue), and the rabbit phosphorylase B was the external standard (yellow).

Supplementary Table 1 Identified proteins from *Histoplasma capsulatum* in mycelium.

Supplementary Table 2 Identified proteins from *Histoplasma capsulatum* in yeast cells.

Supplementary Table 3 Identified proteins from *Histoplasma capsulatum* up-regulated in mycelium compared to yeast cells.

Supplementary Table 4 Identified proteins from *Histoplasma capsulatum* up-regulated in yeast cells compared to mycelium.
