# Supplementary figures and images for: Comparative Proteomic Analysis of Histoplasma capsulatum Yeast and Mycelium Reveals Differential Metabolic Shifts and Cell Wall Remodeling Processes in the Different Morphotypes
Source: Front Microbiol. 2021 Jun 11;12:640931. doi: 10.3389/fmicb.2021.640931 (PMC8226243; doi:10.3389/fmicb.2021.640931)

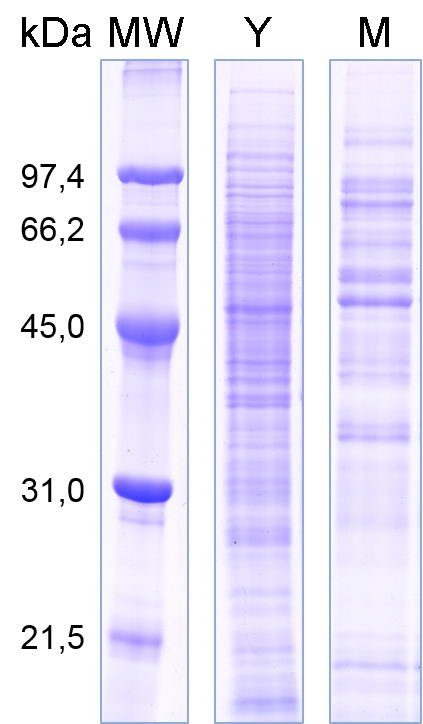

Supplement: Supplementary Figure 1 — SDS-PAGE (12% acrylamide) in Coomassie blue staining. MM, molecular mass; Y, yeast; M, mycelium. Numbers on the left correspond to the molecular mass pattern. [file Image_1.TIF]

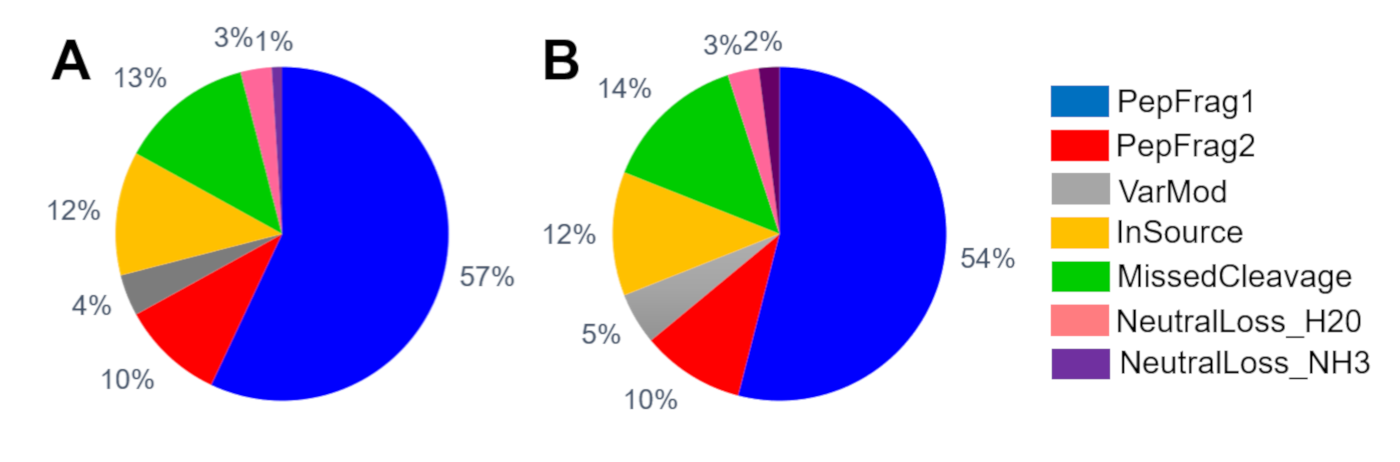

Supplement: Supplementary Figure 2 — Peptide detection type of H. capsulatum mycelia and yeast forms. PepFrag1 and Pepfrag2 – percentage of peptides compared to the H. capsulatum database in ProteinLynx Global Server version 2.4; VarMod, variable modifications; InSource, fragmentation occurred at the ionization source; MissedCleavage, missed cleavage performed by trypsin; and Neutral loss H2O and NH3 correspond to water and ammonia precursor losses. (A,B) Corresponds to yeast and mycelium forms, respectively. [file Image_2.TIF]

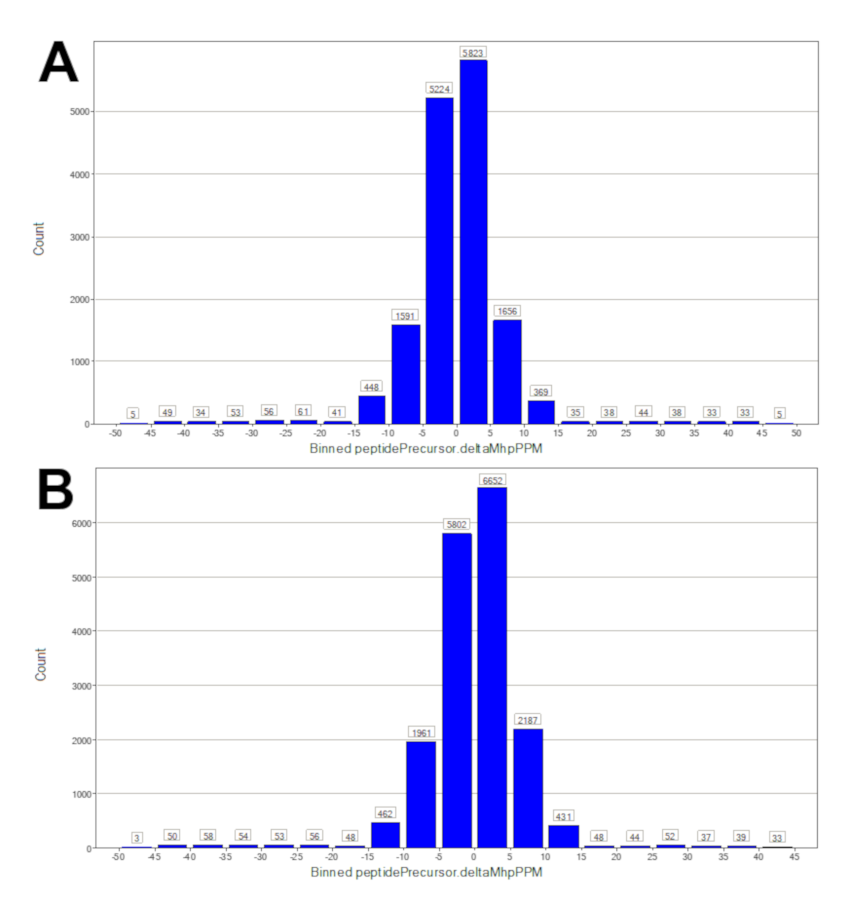

Supplement: Supplementary Figure 3 — Experiment parts per million (ppm) error at the peptide level. The graphs indicate the identified peptides number in ppm. A total of 91.8 and 91.4% identified peptides were detected with an error of less than 10 ppm in yeast (A) and mycelium (B) forms. [file Image_3.TIF]

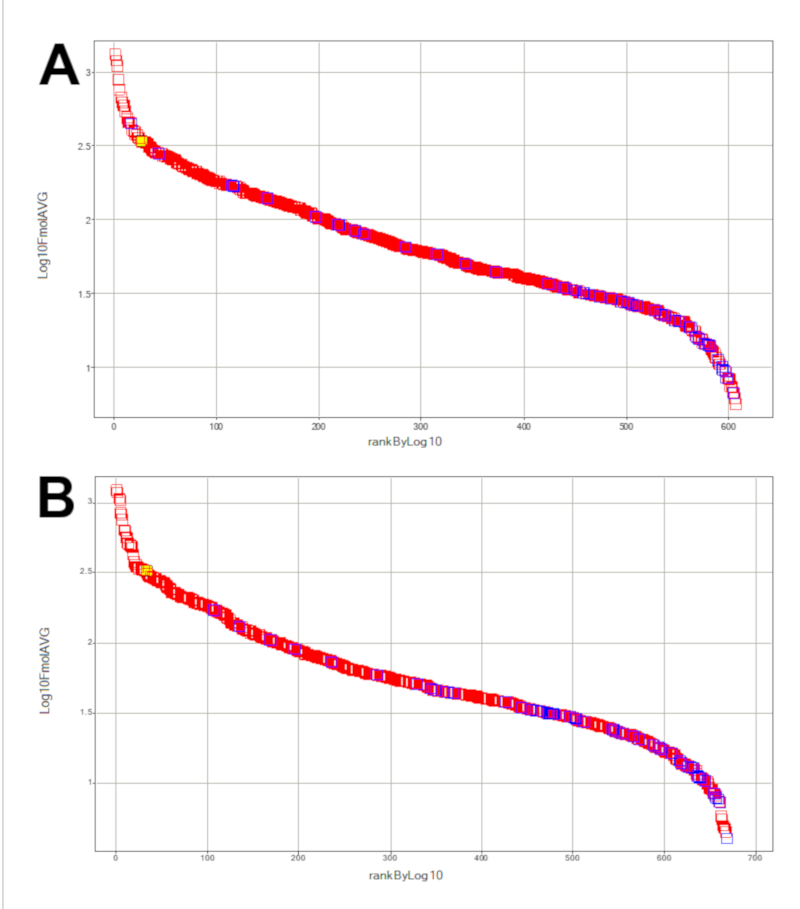

Supplement: Supplementary Figure 4 — Detection dynamic range of proteomic analysis. Graphs for yeast (A) and mycelium (B) were generated, presenting proteins identified in a regular way (red), reverse (blue), and the rabbit phosphorylase B was the external standard (yellow). [file Image_4.TIF]
