# Supplementary material for: Comparative Proteomic Analysis of Histoplasma capsulatum Yeast and Mycelium Reveals Differential Metabolic Shifts and Cell Wall Remodeling Processes in the Different Morphotypes
Source: Front Microbiol. 2021 Jun 11;12:640931. doi: 10.3389/fmicb.2021.640931 (PMC8226243; doi:10.3389/fmicb.2021.640931)
Supplement: Supplementary Table 1 — Identified proteins from Histoplasma capsulatum in mycelium. [file Table_1.DOCX]

**Supplementary Table 1: Identified proteins from *Histoplasma capsulatum* in mycelium**

| **Accession^a^** | **Protein Description^b^** | **Score** | **Quantification (Fmol)** | **Repeat** |
| --- | --- | --- | --- | --- |
| HCAG_05094 | 2 methylcitrate dehydratase | 14535,38 | 329,3534 | 3 |
| HCAG_05090 | 2 methylcitrate synthase | 16035,06 | 620,6811 | 3 |
| HCAG_05761 | 2 oxoisovalerate dehydrogenase alpha subunit | 1112,957 | 69,5762 | 2 |
| HCAG_05409 | 2,3 bisphosphoglycerate independent phosphoglycerate mutase | 505,2018 | 51,13775 | 2 |
| HCAG_00039 | 26S protease regulatory subunit | 521,78 | 40,28955 | 2 |
| HCAG_04173 | 3 family protein | 10359,37 | 313,6312 | 3 |
| HCAG_07725 | 3 hydroxybutyryl coa dehydrogenase | 1796,042 | 69,13264 | 2 |
| HCAG_01093 | 3 hydroxyisobutyrate dehydrogenase | 1743,357 | 38,8947 | 3 |
| HCAG_07104 | 3 isopropylmalate dehydratase | 982,076 | 71,54304 | 3 |
| HCAG_01596 | 3 ketoacyl coa thiolase peroxisomal A | 5890,138 | 140,9069 | 3 |
| HCAG_00678 | 3 methylcrotonyl coa carboxylase biotin containing subunit | 1005,831 | 101,0034 | 3 |
| HCAG_08375 | 4 aminobutyrate aminotransferase | 2586,583 | 130,5627 | 3 |
| HCAG_07773 | 40S ribosomal protein S11 | 2268,264 | 167,6053 | 3 |
| HCAG_06308 | 40S ribosomal protein S12 | 3153,335 | 101,314 | 3 |
| HCAG_08075 | 40S ribosomal protein S14 | 6408,525 | 199,9799 | 3 |
| HCAG_02704 | 40S ribosomal protein S15 | 7132,072 | 126,9721 | 3 |
| HCAG_08092 | 40S ribosomal protein S17 | 11788,51 | 279,7606 | 3 |
| HCAG_08667 | 40S ribosomal protein S18 | 4736,461 | 171,4727 | 3 |
| HCAG_02272 | 40S ribosomal protein S22 | 1703,227 | 172,4262 | 3 |
| HCAG_02186 | 40S ribosomal protein S25 | 8460,757 | 164,6864 | 2 |
| HCAG_05528 | 40s ribosomal protein s26 | 12441,03 | 114,4841 | 3 |
| HCAG_01228 | 40S ribosomal protein S27 | 10055,24 | 207,1882 | 3 |
| HCAG_00214 | 40S ribosomal protein S3 | 12263,16 | 342,8216 | 3 |
| HCAG_06914 | 40S ribosomal protein S3A | 6564,305 | 211,6782 | 3 |
| HCAG_02430 | 40S ribosomal protein S5 A | 7882,723 | 285,1515 | 3 |
| HCAG_06613 | 40S ribosomal protein S7e | 21835,03 | 283,8467 | 3 |
| HCAG_10915 | 40S ribosomal protein S8e | 377,9174 | 101,1142 | 2 |
| HCAG_07249 | 40S ribosomal protein S9 | 10628,59 | 273,1545 | 3 |
| HCAG_05363 | 5 oxoprolinase | 1199,545 | 59,84616 | 3 |
| HCAG_05884 | 6 phosphogluconate dehydrogenase | 13980,23 | 256,0589 | 3 |
| HCAG_04762 | 6 phosphogluconolactonase | 639,3469 | 45,2665 | 3 |
| HCAG_04089 | 6,7 dimethyl 8 ribityllumazine synthase | 450,5972 | 10,8939 | 2 |
| HCAG_02703 | 60S acidic ribosomal protein P2 | 21399,19 | 459,3534 | 3 |
| HCAG_04231 | 60S ribosomal protein | 2621,479 | 184,6273 | 3 |
| HCAG_00468 | 60S ribosomal protein | 10051,12 | 278,1601 | 3 |
| HCAG_01850 | 60S ribosomal protein L1 | 3674,466 | 152,7661 | 3 |
| HCAG_04788 | 60S ribosomal protein L12 | 9150,942 | 98,03977 | 3 |
| HCAG_07708 | 60S ribosomal protein L13 | 5709,767 | 152,4659 | 3 |
| HCAG_03611 | 60S ribosomal protein L15 | 4184,901 | 186,1198 | 3 |
| HCAG_00164 | 60S ribosomal protein L16 | 5285,426 | 150,9131 | 3 |
| HCAG_02327 | 60S ribosomal protein L17 | 3232,029 | 124,4935 | 3 |
| HCAG_03593 | 60S ribosomal protein L18A | 5298,849 | 217,2288 | 3 |
| HCAG_07408 | 60S ribosomal protein L19 | 494,2522 | 164,5588 | 3 |
| HCAG_08515 | 60S ribosomal protein L2 | 2682,03 | 204,5222 | 3 |
| HCAG_04519 | 60S ribosomal protein L23 | 3975,727 | 182,2237 | 3 |
| HCAG_03167 | 60S ribosomal protein L24 | 3306,132 | 160,2266 | 3 |
| HCAG_00055 | 60S ribosomal protein L27 A | 9320,229 | 178,0492 | 3 |
| HCAG_03923 | 60S ribosomal protein L3 | 1918,177 | 172,8798 | 3 |
| HCAG_04318 | 60S ribosomal protein L33 A | 4364,667 | 138,3058 | 3 |
| HCAG_04561 | 60S ribosomal protein L34 | 2622,963 | 141,5885 | 2 |
| HCAG_04185 | 60S ribosomal protein L36 | 5124,866 | 112,371 | 3 |
| HCAG_06534 | 60S ribosomal protein L43 | 2660,205 | 109,6931 | 2 |
| HCAG_07397 | 60S ribosomal protein L44 | 757,6205 | 50,76385 | 2 |
| HCAG_08444 | 60S ribosomal protein L5 | 20890,91 | 355,9934 | 3 |
| HCAG_01793 | 60S ribosomal protein L6 | 4920,458 | 183,8818 | 3 |
| HCAG_08706 | 60S ribosomal protein L7 | 10821,68 | 299,4507 | 3 |
| HCAG_04927 | Acetamidase | 660,6712 | 48,3988 | 2 |
| HCAG_01606 | Acetate coa ligase | 10923,25 | 376,7791 | 3 |
| HCAG_08039 | Acetoacetyl coa reductase | 556,2205 | 53,8854 | 3 |
| HCAG_06005 | Acetolactate synthase | 6257,934 | 181,4687 | 3 |
| HCAG_00814 | Acetyl coa acetyltransferase | 6222,945 | 101,2965 | 3 |
| HCAG_08621 | Acetyl coa acetyltransferase | 1934,402 | 92,3328 | 3 |
| HCAG_00780 | Acetyl-CoA acyltransferase A | 594,4658 | 25,8668 | 2 |
| HCAG_05266 | Aconitase | 8176,834 | 363,2409 | 3 |
| HCAG_08210 | Actin | 4004,194 | 220,2239 | 3 |
| HCAG_09978 | Acyl coa dehydrogenase | 1274,734 | 40,2888 | 3 |
| HCAG_09977 | Acyl coa dehydrogenase | 1808,643 | 39,02885 | 2 |
| HCAG_08510 | Acyl coa dehydrogenase | 2708,308 | 79,6237 | 3 |
| HCAG_08512 | Adenosine kinase | 4412,19 | 152,7267 | 3 |
| HCAG_10265 | Adenosylhomocysteinase | 20246,8 | 282,6097 | 3 |
| HCAG_01433 | Adenylosuccinate lyase | 904,8631 | 74,99776 | 3 |
| HCAG_06743 | Adenylosuccinate synthetase adb | 446,8559 | 25,8415 | 2 |
| HCAG_06283 | Adp ATP carrier protein | 899,5107 | 84,60615 | 2 |
| HCAG_01847 | ADP ribosylation factor | 4785,139 | 130,7831 | 2 |
| HCAG_08426 | Aflatoxin B1 aldehyde reductase member 3 | 1726,297 | 29,3097 | 2 |
| HCAG_04426 | AIF like mitochondrial oxidoreductase | 1554,428 | 105,5596 | 3 |
| HCAG_01578 | Alcohol dehydrogenase | 1022,443 | 49,0853 | 2 |
| HCAG_02317 | Alcohol dehydrogenase | 2333,302 | 60,54857 | 3 |
| HCAG_06397 | Alcohol dehydrogenase | 4000,911 | 145,3657 | 3 |
| HCAG_05083 | Alcohol dehydrogenase | 484,7904 | 86,3563 | 3 |
| HCAG_08561 | Alcohol dehydrogenase | 1717,925 | 102,6661 | 3 |
| HCAG_00576 | Alcohol dehydrogenase | 570,2202 | 27,4746 | 2 |
| HCAG_02371 | Aldehyde dehydrogenase | 9548,739 | 479,7115 | 3 |
| HCAG_06205 | Aldehyde dehydrogenase | 1162,125 | 69,94363 | 3 |
| HCAG_00980 | Alkaline phosphatase | 1848,132 | 73,92933 | 3 |
| HCAG_03188 | Allergen Asp F3 | 4377,332 | 92,9481 | 2 |
| HCAG_01535 | Alpha-ketoglutarate dehydrogenase | 370,4913 | 58,32165 | 2 |
| HCAG_04617 | Amino transferase | 886,9272 | 44,94923 | 3 |
| HCAG_06935 | Aminopeptidase | 2073,67 | 139,8825 | 3 |
| HCAG_07083 | Aminopeptidase | 1300,714 | 101,0619 | 3 |
| HCAG_05224 | Anthranilate synthase component I | 889,6423 | 46,24356 | 3 |
| HCAG_00035 | Arginase | 1191,142 | 65,40557 | 3 |
| HCAG_07805 | Argininosuccinate lyase | 445,6001 | 32,26823 | 3 |
| HCAG_06683 | Argininosuccinate synthetase | 4144,891 | 132,9179 | 3 |
| HCAG_04706 | ARP2 3 complex subunit | 681,519 | 22,11807 | 3 |
| HCAG_08678 | Aspartate aminotransferase | 1859,221 | 101,4998 | 3 |
| HCAG_06102 | Aspartate aminotransferase | 1829,467 | 123,0136 | 3 |
| HCAG_00037 | Aspartic endopeptidase Pep2 | 3331,652 | 224,2575 | 3 |
| HCAG_04297 | Aspartyl aminopeptidase | 599,9355 | 89,45097 | 3 |
| HCAG_10740 | Aspartyl trna synthetase | 408,0021 | 31,12613 | 3 |
| HCAG_04933 | ATP citrate lyase | 1465,942 | 144,323 | 3 |
| HCAG_04934 | ATP citrate synthase subunit 1 | 2515,303 | 133,7744 | 3 |
| HCAG_04273 | ATP dependent RNA helicase eif4a | 1906,92 | 46,98973 | 3 |
| HCAG_06360 | ATP phosphoribosyltransferase | 1931,301 | 38,05836 | 3 |
| HCAG_04799 | ATP synthase gamma chain | 3571,415 | 82,10416 | 3 |
| HCAG_02828 | ATP synthase subunit 4 | 914,0566 | 61,72365 | 2 |
| HCAG_02813 | ATP synthase subunit alpha | 9441,877 | 335,7782 | 3 |
| HCAG_07132 | BAR domain containing protein | 907,6732 | 45,2939 | 2 |
| HCAG_02260 | Beta isopropylmalate dehydrogenase | 924,9158 | 111,5034 | 3 |
| HCAG_04224 | Bifunctional purine biosynthesis protein ADE16 | 2698,355 | 117,0407 | 3 |
| HCAG_03336 | Branched chain alpha keto acid dehydrogenase E1 subunit beta | 1219,174 | 61,6188 | 2 |
| HCAG_03302 | Branched chain amino acid aminotransferase | 3702,893 | 147,9276 | 3 |
| HCAG_08945 | Branched chain amino acid aminotransferase | 1279,736 | 43,1891 | 3 |
| HCAG_04357 | C 1 tetrahydrofolate synthase | 506,8049 | 60,80383 | 3 |
| HCAG_02700 | Carbamoyl phosphate synthase | 349,696 | 36,00227 | 3 |
| HCAG_04508 | Carboxypeptidase | 707,7655 | 76,97805 | 2 |
| HCAG_03790 | Carnitine acetyl transferase | 1589,52 | 87,55869 | 3 |
| HCAG_05109 | Catalase | 830,4074 | 71,2571 | 3 |
| HCAG_08064 | Catalase B | 7718,121 | 458,736 | 3 |
| HCAG_08345 | Cell cycle control protein | 2857,38 | 109,1026 | 3 |
| HCAG_02452 | Cell division cycle protein | 3953,202 | 125,5184 | 3 |
| HCAG_00290 | Chorismate mutase | 457,0327 | 30,18975 | 2 |
| HCAG_06981 | Citrate synthase | 5206,978 | 129,2851 | 3 |
| HCAG_05565 | Cobalamin independent methionine synthase meth D | 17091,59 | 396,1695 | 3 |
| HCAG_08732 | Conserved hypothetical protein | 11369,82 | 232,6765 | 3 |
| HCAG_04397 | Conserved hypothetical protein | 31733,56 | 492,436 | 3 |
| HCAG_05185 | Conserved hypothetical protein | 10186,69 | 145,7858 | 3 |
| HCAG_04453 | Conserved hypothetical protein | 11834,89 | 328,9261 | 3 |
| HCAG_06099 | Conserved hypothetical protein | 1716,874 | 20,9307 | 2 |
| HCAG_11214 | Conserved hypothetical protein | 1072,553 | 40,8104 | 3 |
| HCAG_06375 | Conserved hypothetical protein | 1834,543 | 67,5463 | 3 |
| HCAG_06271 | Conserved hypothetical protein | 1396,959 | 83,15905 | 2 |
| HCAG_04776 | Conserved hypothetical protein | 1924,963 | 45,5018 | 3 |
| HCAG_06999 | Conserved hypothetical protein | 921,8329 | 50,8253 | 2 |
| HCAG_00803 | Conserved hypothetical protein | 2875,682 | 175,5884 | 3 |
| HCAG_01543 | Conserved hypothetical protein | 1031,696 | 63,14527 | 3 |
| HCAG_07305 | Conserved hypothetical protein | 3858,993 | 141,7529 | 3 |
| HCAG_08357 | Conserved hypothetical protein | 0,21985 | 0 | 2 |
| HCAG_03758 | Conserved hypothetical protein | 748,5057 | 47,87605 | 2 |
| HCAG_08106 | Conserved hypothetical protein | 6854,198 | 291,7614 | 3 |
| HCAG_02919 | Conserved hypothetical protein | 18296,61 | 288,0587 | 3 |
| HCAG_01717 | Conserved hypothetical protein | 582,5349 | 76,71436 | 3 |
| HCAG_01715 | Conserved hypothetical protein | 430,4391 | 22,44875 | 2 |
| HCAG_05962 | Conserved hypothetical protein | 397,2354 | 42,23805 | 2 |
| HCAG_08367 | Conserved hypothetical protein | 24719,58 | 892,1055 | 3 |
| HCAG_11293 | Conserved hypothetical protein | 2373,276 | 176,1933 | 3 |
| HCAG_06889 | Conserved hypothetical protein | 555,1629 | 43,21313 | 3 |
| HCAG_01864 | Copper amine oxidase | 946,0996 | 100,0781 | 3 |
| HCAG_00642 | Copper zinc superoxide dismutase | 15063,14 | 268,1146 | 3 |
| HCAG_06523 | Curved DNA binding protein | 2367,092 | 171,3637 | 3 |
| HCAG_07434 | Cystathionine beta synthase | 563,2045 | 32,7928 | 2 |
| HCAG_07539 | Cytochrome b2 | 860,3229 | 39,97995 | 2 |
| HCAG_09319 | Cytochrome c peroxidase | 5472,729 | 85,9399 | 2 |
| HCAG_07098 | Cytochrome c peroxidase | 675,1393 | 63,63453 | 3 |
| HCAG_03694 | Cytosolic large ribosomal subunit protein L30 | 12073,23 | 207,4935 | 3 |
| HCAG_01780 | Delta 1 pyrroline 5 carboxylate dehydrogenase prnc | 1509,328 | 157,907 | 2 |
| HCAG_09000 | Delta 1 pyrroline 5 carboxylate dehydrogenase prnc | 2301,627 | 100,3765 | 3 |
| HCAG_03972 | Dihydrolipoamide acetyltransferase component | 1265,468 | 85,90917 | 3 |
| HCAG_01491 | Dihydrolipoamide branched chain transacylase E2 | 1058,104 | 58,778 | 2 |
| HCAG_08825 | Dihydrolipoamide dehydrogenase | 4753,039 | 145,0324 | 3 |
| HCAG_03522 | Dihydrolipoamide succinyltransferase | 823,386 | 58,3916 | 3 |
| HCAG_03216 | Dihydroorotase | 456,6449 | 20,52707 | 3 |
| HCAG_02555 | Dihydroxyacetone kinase | 469,6963 | 30,04223 | 3 |
| HCAG_08942 | Dipeptidyl peptidase III | 610,2379 | 51,0808 | 3 |
| HCAG_05596 | Diphosphomevalonate decarboxylase | 1261,119 | 39,7084 | 3 |
| HCAG_04527 | DNA damage checkpoint protein rad24 | 14364,2 | 273,2786 | 3 |
| HCAG_05805 | DNAK molecular chaperone bipa | 1577,313 | 137,0227 | 3 |
| HCAG_07572 | DUF427 domain containing protein | 8885,566 | 281,0556 | 3 |
| HCAG_07065 | DUF757 domain containing protein | 1260,291 | 29,68417 | 3 |
| HCAG_03847 | Electron transfer flavoprotein alpha subunit | 1121,552 | 75,21796 | 3 |
| HCAG_02931 | Electron transfer flavoprotein beta subunit | 474,0967 | 43,39253 | 3 |
| HCAG_01917 | Elicitor protein | 4095,753 | 201,6862 | 3 |
| HCAG_01784 | Elongation factor 1 beta | 10332,55 | 314,6603 | 3 |
| HCAG_08236 | Elongation factor 1 gamma | 4988,462 | 162,1948 | 3 |
| HCAG_05988 | Elongation factor 2 | 8982,82 | 332,0243 | 3 |
| HCAG_03444 | Elongation factor Tu | 715,2623 | 40,39155 | 2 |
| HCAG_05757 | Endonuclease exonuclease phosphatase family protein | 959,1799 | 59,1693 | 3 |
| HCAG_08058 | Enoyl coa hydratase isomerase family protein | 2925,334 | 140,7189 | 3 |
| HCAG_04675 | Esterase D | 743,4541 | 95,5532 | 2 |
| HCAG_04139 | Eukaryotic phosphomannomutase | 833,2692 | 0 | 2 |
| HCAG_04139 | Eukaryotic phosphomannomutase | 1753,029 | 66,2838 | 3 |
| HCAG_03286 | Eukaryotic translation initiation factor 3 | 561,7601 | 37,32055 | 2 |
| HCAG_04356 | Eukaryotic translation initiation factor 3 subunit 2 | 595,5848 | 25,85705 | 2 |
| HCAG_11321 | FAD dependent oxidoreductase | 804,491 | 51,9047 | 2 |
| HCAG_00413 | Farnesyl pyrophosphate synthetase | 1608,288 | 98,53577 | 3 |
| HCAG_07636 | Fatty acid synthase alpha subunit fasa | 575,1721 | 81,3917 | 3 |
| HCAG_07637 | Fatty acid synthase beta subunit dehydratase | 852,2354 | 80,87833 | 3 |
| HCAG_07972 | FKBP type peptidyl prolyl isomerase putative | 2645,194 | 62,90073 | 3 |
| HCAG_08831 | Formamidase | 20893,82 | 1309,6 | 3 |
| HCAG_00010 | Fructose 1 6 biphosphate aldolase | 19061,63 | 594,4607 | 3 |
| HCAG_03322 | Fructose 1 6 bisphosphatase | 5285,171 | 187,6622 | 3 |
| HCAG_08789 | Fructose-bisphosphate aldolase | 749,9816 | 23,12893 | 3 |
| HCAG_08493 | Fumarate hydratase class II | 10235,79 | 292,2123 | 3 |
| HCAG_03323 | Fumarate reductase flavoprotein subunit | 7494,181 | 256,8914 | 3 |
| HCAG_02120 | Fumarylacetoacetase hydrolase family protein | 3283,069 | 185,8748 | 3 |
| HCAG_07031 | G protein comlpex beta subunit cpcb | 11282,84 | 339,8324 | 3 |
| HCAG_06177 | Gamma glutamyltranspeptidase | 526,0381 | 58,23315 | 2 |
| HCAG_03191 | Glucokinase | 373,2939 | 28,3911 | 3 |
| HCAG_04329 | Glucose 6 phosphate 1 dehydrogenase | 829,2949 | 65,74287 | 3 |
| HCAG_08202 | Glucose 6 phosphate isomerase | 4130,857 | 183,4036 | 3 |
| HCAG_08670 | Glucose methanol choline oxidoreductase | 896,7271 | 82,27527 | 3 |
| HCAG_03543 | Glutamate carboxypeptidase | 8651,085 | 270,0707 | 3 |
| HCAG_08662 | Glutamate decarboxylase | 827,3544 | 43,65443 | 3 |
| HCAG_07206 | Glutathione dependent formaldehyde dehydrogenase | 1249,904 | 151,9206 | 3 |
| HCAG_01006 | Glutathione S transferase | 1289,724 | 55,5599 | 3 |
| HCAG_04910 | Glyceraldehyde 3 phosphate dehydrogenase | 49020,48 | 1104,176 | 3 |
| HCAG_03267 | Glycerol 3 phosphate dehydrogenase | 1356,338 | 72,08383 | 3 |
| HCAG_07571 | Glycine dehydrogenase | 1945,591 | 106,2676 | 3 |
| HCAG_02914 | Glycine rich protein | 6648,504 | 191,7505 | 3 |
| HCAG_05187 | GTP binding nuclear protein GSP1 Ran | 8724,307 | 151,5491 | 3 |
| HCAG_01447 | GTP binding protein | 423,7395 | 29,68835 | 2 |
| HCAG_08408 | HAD superfamily hydrolase | 15837,67 | 538,5853 | 3 |
| HCAG_04686 | Heat shock protein | 8155,877 | 191,1247 | 3 |
| HCAG_00806 | Heat shock protein SSB1 | 3750,913 | 152,6033 | 3 |
| HCAG_08176 | Heat shock protein SSC1 | 3421,905 | 120,984 | 3 |
| HCAG_04471 | Heat shock protein STI1 | 1266,735 | 58,921 | 3 |
| HCAG_01131 | Het c2 protein | 465,9147 | 21,53045 | 2 |
| HCAG_03661 | Hexokinase hxk Aspergillus niger | 1001,208 | 59,61147 | 3 |
| HCAG_08082 | HHE domain containing protein | 1447,131 | 26,7777 | 3 |
| HCAG_03525 | Histone H2b | 1871,868 | 93,55044 | 3 |
| HCAG_03885 | Histone h4 | 663,4111 | 38,8617 | 2 |
| HCAG_05721 | Homogentisate 1 2 dioxygenase | 1293,736 | 115,7595 | 3 |
| HCAG_04943 | Hsp10 like protein | 2736,095 | 91,822 | 3 |
| HCAG_06961 | Hsp60 like protein | 6385,055 | 177,1423 | 3 |
| HCAG_01398 | Hsp70 like protein | 16194,24 | 455,0894 | 3 |
| HCAG_00783 | Hsp88 like protein | 2728,837 | 94,04237 | 3 |
| HCAG_02530 | Hydroxyacylglutathione hydrolase | 925,4011 | 54,70835 | 2 |
| HCAG_08805 | Hydroxymethylglutaryl coa synthase | 933,5016 | 86,13765 | 2 |
| HCAG_05533 | Hypothetical protein | 15199,45 | 193,9634 | 3 |
| HCAG_07506 | Hypothetical protein | 13566,85 | 120,9035 | 3 |
| HCAG_06895 | Hypothetical protein | 1054,513 | 78,41463 | 3 |
| HCAG_08057 | Hypothetical protein | 2065,569 | 87,04247 | 3 |
| HCAG_06361 | Hypothetical protein | 26314,31 | 222,7411 | 3 |
| HCAG_06110 | Immunogenic protein | 1972,064 | 136,1923 | 3 |
| HCAG_04432 | Indoleamine 2 3 dioxygenase family protein | 1806,859 | 60,20073 | 3 |
| HCAG_04307 | Inorganic pyrophosphatase | 713,3729 | 42,4819 | 2 |
| HCAG_10958 | Isocitrate lyase | 6642,721 | 153,6943 | 3 |
| HCAG_05089 | Isocitrate lyase | 811,584 | 72,62807 | 3 |
| HCAG_06694 | Isoleucyl trna synthetase cytoplasmic | 510,1708 | 62,37294 | 3 |
| HCAG_00677 | Isovaleryl coa dehydrogenase | 1284,676 | 76,4362 | 3 |
| HCAG_08890 | Ketol acid reductoisomerase | 5126,488 | 228,6571 | 3 |
| HCAG_06295 | KH domain RNA binding protein | 2010,071 | 45,01534 | 3 |
| HCAG_00626 | L xylulose reductase | 7742,359 | 237,2688 | 3 |
| HCAG_01368 | Lactoylglutathione lyase | 3072,072 | 84,62014 | 3 |
| HCAG_05713 | Lysyl trna synthetase | 1731,838 | 74,5043 | 3 |
| HCAG_06901 | Malate dehydrogenase | 19050,05 | 260,1951 | 3 |
| HCAG_03969 | Malate dehydrogenase | 31711 | 363,2881 | 3 |
| HCAG_05084 | Malate synthase | 3790,179 | 170,7948 | 3 |
| HCAG_03448 | Manganese superoxide dismutase | 6853,66 | 265,6224 | 3 |
| HCAG_08720 | Mannitol 1 phosphate dehydrogenase | 7237,526 | 220,0057 | 3 |
| HCAG_08436 | Mannose 6 phosphate isomerase | 1291,541 | 39,23697 | 3 |
| HCAG_00676 | Methylcrotonoyl coa carboxylase beta chain | 928,3769 | 57,99833 | 3 |
| HCAG_06059 | Methylmalonate semialdehyde dehydrogenase | 12767,66 | 377,3372 | 3 |
| HCAG_05584 | Mitochondrial acetolactate synthase small subunit | 639,6602 | 22,2244 | 2 |
| HCAG_06944 | Mitochondrial ATP synthase | 17559,9 | 334,6869 | 3 |
| HCAG_00064 | N acetylglucosamine phosphate mutase | 1657,187 | 53,62407 | 3 |
| HCAG_11124 | NAD dependent formate dehydrogenase acia Fdh | 9185,157 | 594,4974 | 3 |
| HCAG_04093 | NAD isocitrate dehydrogenase subunit I | 980,3948 | 20,32615 | 2 |
| HCAG_03605 | NADP dependent mannitol dehydrogenase | 30627,26 | 672,968 | 3 |
| HCAG_05099 | Nascent polypeptide associated complex subunit alpha | 3692,202 | 112,4443 | 3 |
| HCAG_05051 | Nascent polypeptide associated complex subunit beta | 4437,551 | 61,95777 | 3 |
| HCAG_03497 | Nicotinate nucleotide pyrophosphorylase | 1964,994 | 82,313 | 3 |
| HCAG_07200 | Nitrate reductase | 1082,796 | 57,6576 | 3 |
| HCAG_07558 | Nmra like family domain containing protein | 986,0771 | 62,71065 | 2 |
| HCAG_08974 | Nonspecific lipid transfer protein | 810,8122 | 61,20343 | 3 |
| HCAG_00131 | Nuclear transport factor 2 | 1311,066 | 62,7095 | 2 |
| HCAG_00544 | Nucleoside diphosphate kinase | 11436,46 | 255,8506 | 3 |
| HCAG_03106 | Nucleosome assembly protein | 1708,47 | 67,73977 | 3 |
| HCAG_00018 | Nucleosome binding protein | 1079,603 | 66,92924 | 2 |
| HCAG_07004 | O acetylhomoserine | 1248,258 | 47,57994 | 3 |
| HCAG_02754 | Oligopeptidase family protein | 1674,133 | 35,7242 | 3 |
| HCAG_00429 | Oxidoreductase | 1396,88 | 51,9609 | 2 |
| HCAG_00889 | Oxidoreductase | 6165,615 | 147,1975 | 3 |
| HCAG_05959 | Oxidoreductase | 833,6923 | 35,4285 | 2 |
| HCAG_08156 | Oxidoreductase | 1067,854 | 55,66087 | 3 |
| HCAG_08190 | Oxidoreductase 2 nitropropane dioxygenase | 2209,618 | 126,1198 | 3 |
| HCAG_06234 | PEP phosphonomutase | 821,0222 | 35,2191 | 2 |
| HCAG_08833 | Peptidyl prolyl cis trans isomerase | 1354,984 | 53,55167 | 3 |
| HCAG_04215 | Peptidyl prolyl cis trans isomerase B | 1366,089 | 25,85685 | 2 |
| HCAG_04485 | Peptidylprolyl isomerase | 20365,25 | 1203,366 | 3 |
| HCAG_00067 | Peroxisomal dehydratase | 1348,881 | 46,02555 | 2 |
| HCAG_04358 | Peroxisomal NADP dependent isocitrate dehydrogenase | 4923,432 | 131,7134 | 3 |
| HCAG_06371 | Phenylacetyl coa ligase | 616,4225 | 28,739 | 2 |
| HCAG_04499 | Phenylalanyl trna synthetase | 596,5448 | 30,8062 | 2 |
| HCAG_03270 | Phospho 2 dehydro 3 deoxyheptonate aldolase | 937,7212 | 47,5069 | 2 |
| HCAG_08116 | Phospho 2 dehydro 3 deoxyheptonate aldolase | 578,5984 | 32,21827 | 3 |
| HCAG_05681 | Phosphoenolpyruvate carboxykinase acuf | 1745,127 | 143,1986 | 3 |
| HCAG_07552 | Phosphofructokinase | 502,8675 | 26,34973 | 3 |
| HCAG_08808 | Phosphoglucomutase | 937,7065 | 67,73376 | 3 |
| HCAG_03385 | Phosphoglycerate kinase | 10755,49 | 395,0895 | 3 |
| HCAG_06026 | Polyadenylate binding protein | 611,8305 | 65,18793 | 3 |
| HCAG_02662 | Porphobilinogen deaminase | 618,114 | 37,32035 | 2 |
| HCAG_08909 | Predicted protein | 3605,773 | 123,4843 | 3 |
| HCAG_02758 | Predicted protein | 4224,784 | 172,4829 | 3 |
| HCAG_04835 | Proliferating cell nuclear antigen | 2562,088 | 124,9164 | 3 |
| HCAG_04190 | Proteasome component | 1988,271 | 37,29025 | 2 |
| HCAG_06342 | Proteasome component | 515,4178 | 58,05695 | 2 |
| HCAG_04101 | Proteasome component | 2018,485 | 63,20977 | 3 |
| HCAG_00347 | Proteasome component | 2755,601 | 60,51663 | 3 |
| HCAG_03939 | Proteasome component | 752,1971 | 51,20415 | 2 |
| HCAG_03630 | Proteindisulfidisomerase | 2207,747 | 169,5233 | 3 |
| HCAG_02994 | Pyridoxine biosynthesis protein pyroa validated | 8846,832 | 151,6493 | 3 |
| HCAG_04227 | Pyruvate carboxylase | 2930,833 | 122,8378 | 3 |
| HCAG_08778 | Pyruvate decarboxylase | 1870,131 | 106,9793 | 3 |
| HCAG_01360 | Pyruvate dehydrogenase E1 component alpha subunit | 1658,188 | 94,74577 | 3 |
| HCAG_07619 | Pyruvate dehydrogenase E1 component beta subunit | 2820,377 | 103,0271 | 3 |
| HCAG_07781 | Pyruvate kinase | 6260,613 | 119,3218 | 3 |
| HCAG_06198 | QDE2 protein | 756,4883 | 36,57727 | 3 |
| HCAG_02612 | Ran specific gtpase activating protein | 1781,451 | 96,45235 | 2 |
| HCAG_03115 | Rheb small monomeric gtpase rhba | 735,4984 | 20,55965 | 2 |
| HCAG_05560 | Rho gtpase | 505,885 | 29,5225 | 3 |
| HCAG_04939 | Ribose 5 phosphate isomerase A | 675,9375 | 36,71054 | 3 |
| HCAG_03695 | Ribosomal L10 protein | 6528,958 | 241,6813 | 3 |
| HCAG_03504 | Ribosomal protein L22 | 4629,596 | 171,2169 | 3 |
| HCAG_04987 | Ribosomal protein l22e | 5001,365 | 196,4432 | 3 |
| HCAG_03055 | Ribosomal protein L23a | 3054,514 | 136,3122 | 3 |
| HCAG_05192 | Ribosomal protein L31e | 6498,029 | 212,2969 | 3 |
| HCAG_06425 | Ribosomal protein L32 | 9567,836 | 213,3697 | 3 |
| HCAG_05221 | Ribosomal protein L7a | 9853,339 | 253,0529 | 3 |
| HCAG_04856 | Ribosomal protein P0 | 4780,641 | 229,6509 | 3 |
| HCAG_04662 | Ribosomal protein S13 | 10046,71 | 219,3145 | 3 |
| HCAG_04575 | Ribosomal protein S16 | 9060,794 | 271,8549 | 3 |
| HCAG_08821 | Ribosomal protein S20 | 4770,102 | 202,9201 | 3 |
| HCAG_04498 | Ribosomal protein s21e | 11731,47 | 239,1255 | 3 |
| HCAG_01947 | Ribosomal protein S23 | 1814,796 | 145,8665 | 3 |
| HCAG_07237 | Ribosomal protein S4 | 6952,087 | 336,9375 | 3 |
| HCAG_07961 | Ribosomal protein S5 | 7712,412 | 307,4359 | 3 |
| HCAG_01666 | Ribosomal protein S6 | 3240,884 | 115,4239 | 3 |
| HCAG_08073 | Ribosomal protein S9 | 9489,132 | 201,4683 | 3 |
| HCAG_04410 | RNA binding protein | 1484,292 | 85,57104 | 3 |
| HCAG_03428 | S adenosylmethionine synthetase | 4126,722 | 135,0952 | 3 |
| HCAG_01145 | Saccharopine dehydrogenase | 2468,422 | 124,9878 | 3 |
| HCAG_01575 | Saccharopine reductase | 5558,524 | 168,2124 | 3 |
| HCAG_05465 | Sec14 cytosolic factor | 607,2944 | 40,61875 | 2 |
| HCAG_07830 | Secretory pathway gdp dissociation inhibitor | 2555,296 | 88,19006 | 3 |
| HCAG_03836 | Septin | 873,0353 | 47,7895 | 3 |
| HCAG_00717 | Septin 1 | 1259,944 | 66,00933 | 3 |
| HCAG_00459 | Septin 2 | 872,6402 | 75,16444 | 3 |
| HCAG_02006 | Septin 3 | 1102,727 | 45,80217 | 3 |
| HCAG_05408 | Serine hydroxymethyltransferase | 3565,471 | 79,79577 | 3 |
| HCAG_07418 | Serine hydroxymethyltransferase | 1302,793 | 93,37479 | 3 |
| HCAG_00635 | Serine proteinase | 642,156 | 71,48815 | 2 |
| HCAG_04969 | Serine threonine protein phosphatase | 621,7549 | 25,05765 | 2 |
| HCAG_05071 | Short chain dehydrogenase | 1549,844 | 26,61573 | 3 |
| HCAG_04443 | Short chain dehydrogenase reductase | 3210,883 | 45,04783 | 3 |
| HCAG_00118 | Short chain dehydrogenase reductase | 841,5723 | 60,64174 | 3 |
| HCAG_04999 | Spermidine synthase | 5559,919 | 244,5098 | 3 |
| HCAG_05184 | Stress protein DDR48 | 2002,505 | 230,4442 | 3 |
| HCAG_03348 | Stress responsive A B barrel domain containing protein | 727,504 | 25,11937 | 3 |
| HCAG_10301 | Suaprga1 | 975,5952 | 75,4578 | 3 |
| HCAG_06317 | Succinate dehydrogenase | 620,0397 | 43,2296 | 3 |
| HCAG_07261 | Succinate semialdehyde dehydrogenase | 1376,718 | 54,78003 | 3 |
| HCAG_05311 | Succinyl coa 3 ketoacid coenzyme A transferase | 1229,656 | 91,72137 | 3 |
| HCAG_07697 | Succinyl coa ligase beta chain | 1143,488 | 51,70573 | 3 |
| HCAG_01212 | Sulfate adenylyltransferase | 961,9276 | 55,6312 | 3 |
| HCAG_03008 | Sulfur metabolite repression control protein | 5106,448 | 63,3235 | 3 |
| HCAG_05697 | T complex protein 1 epsilon subunit | 871,2639 | 37,58537 | 3 |
| HCAG_06315 | T complex protein 1 subunit delta | 610,7462 | 36,2257 | 2 |
| HCAG_02143 | TCTP family protein | 10244,84 | 126,6398 | 3 |
| HCAG_03261 | Thiamine biosynthesis protein | 1754,254 | 64,37814 | 3 |
| HCAG_05915 | Thiazole biosynthetic enzyme | 6234,246 | 265,9257 | 3 |
| HCAG_06128 | Thij pfpi family protein | 4375,741 | 258,2695 | 3 |
| HCAG_06210 | Thiol specific antioxidant | 11149,17 | 190,9329 | 3 |
| HCAG_00878 | Thioredoxin domain containing protein | 26346,34 | 171,0643 | 3 |
| HCAG_07096 | Thioredoxin domain containing protein | 610,1503 | 24,9219 | 2 |
| HCAG_00793 | Threonine synthase | 1258,825 | 35,2403 | 2 |
| HCAG_00638 | Transaldolase | 3553,865 | 227,0938 | 3 |
| HCAG_01318 | Transcription factor rfef putative | 5576,498 | 181,5082 | 3 |
| HCAG_05000 | Transketolase tkta | 2826,619 | 166,641 | 3 |
| HCAG_08798 | Translation elongation factor 1 alpha | 13347,61 | 761,584 | 3 |
| HCAG_06021 | Translation initiation factor eif 5A putative | 1677,034 | 40,08437 | 3 |
| HCAG_02511 | Triosephosphate isomerase | 10974,52 | 213,1973 | 3 |
| HCAG_01781 | Tubulin beta chain | 717,8038 | 35,5011 | 3 |
| HCAG_08288 | Tubulin subunit alpha 2 | 572,0717 | 31,2687 | 2 |
| HCAG_04336 | Tyrosinase | 808,3245 | 83,5613 | 3 |
| HCAG_06019 | Ubiquitin | 11173,3 | 111,632 | 2 |
| HCAG_09628 | Ubiquitin activating enzyme E1 | 740,4038 | 40,1133 | 3 |
| HCAG_06566 | Ubiquitin fusion protein | 11227,73 | 405,2535 | 3 |
| HCAG_06641 | UDP galactopyranose mutase | 682,4587 | 33,6422 | 3 |
| HCAG_09614 | UDP glucose 4 epimerase | 1282,239 | 61,382 | 3 |
| HCAG_09613 | UDP glucose 4 epimerase Gal10 | 1639,242 | 56,6437 | 3 |
| HCAG_04416 | UDP N acetylglucosamine pyrophosphorylase | 2250,071 | 78,625 | 3 |
| HCAG_06192 | UPF0010 domain containing protein | 1321,759 | 43,6643 | 3 |
| HCAG_00404 | Vacuolar ATP synthase catalytic subunit A | 571,5181 | 48,4494 | 3 |
| HCAG_06619 | Vacuolar sorting associated protein | 598,0339 | 29,11115 | 2 |
| HCAG_02992 | Xaa pro aminopeptidase | 1285,528 | 73,3595 | 3 |
| HCAG_07700 | Xanthine phosphoribosyltransferase | 800,2833 | 31,5309 | 2 |
| HCAG_04745 | Y20 protein | 37765,37 | 241,6923 | 3 |

^a^ Identification of proteins from *Histoplasma* genome database using the ProteinLynx Global Server vs. 2.4 (PLGS) (Waters Corporation, Manchester, UK) (http://www.broadinstitute.org/annotation/genome/histoplasma_capsulatum/MultiHome.html).

^b^ Genes annotation from *Histoplasma* genome database or by homology from NCBI database (http://www.ncbi.nlm.nih.gov/).
