# Supplementary material for: Comparative Proteomic Analysis of Histoplasma capsulatum Yeast and Mycelium Reveals Differential Metabolic Shifts and Cell Wall Remodeling Processes in the Different Morphotypes
Source: Front Microbiol. 2021 Jun 11;12:640931. doi: 10.3389/fmicb.2021.640931 (PMC8226243; doi:10.3389/fmicb.2021.640931)
Supplement: Supplementary Table 2 — Identified proteins from Histoplasma capsulatum in yeast cells. [file Table_2.DOCX]

**Supplementary Table 2: Identified proteins from *Histoplasma capsulatum* in yeast cells**

| **Accession^a^** | **Protein Description^b^** | **Score** | **Quantification (Fmol)** | **Repeat** |
| --- | --- | --- | --- | --- |
| HCAG_09984 | 2 isopropylmalate synthase | 809,6718 | 41,80215 | 2 |
| HCAG_05094 | 2 methylcitrate dehydratase | 8720,755 | 189,0741 | 3 |
| HCAG_05090 | 2 methylcitrate synthase | 4578,922 | 221,3285 | 3 |
| HCAG_05409 | 2,3 bisphosphoglycerate independent phosphoglycerate mutase | 492,3557 | 45,6814 | 3 |
| HCAG_02901 | 26S protease regulatory subunit | 360,6088 | 22,3046 | 2 |
| HCAG_07682 | 26S protease regulatory subunit | 402,9584 | 27,39077 | 3 |
| HCAG_04181 | 26S protease regulatory subunit | 273,1039 | 8,113267 | 3 |
| HCAG_00039 | 26S protease regulatory subunit | 1058,045 | 20,39897 | 3 |
| HCAG_00069 | 26S protease regulatory subunit | 1344,447 | 26,74167 | 3 |
| HCAG_04173 | 3 family protein | 36641,64 | 754,6643 | 3 |
| HCAG_01093 | 3 hydroxyisobutyrate dehydrogenase | 1949,321 | 54,67277 | 3 |
| HCAG_02524 | 3 hydroxyisobutyryl coa hydrolase | 2429,474 | 33,63607 | 3 |
| HCAG_07104 | 3 isopropylmalate dehydratase | 2260,901 | 101,7379 | 3 |
| HCAG_01596 | 3 ketoacyl coa thiolase peroxisomal A | 4010,7 | 86,12136 | 3 |
| HCAG_07773 | 40S ribosomal protein S11 | 2419,144 | 134,811 | 3 |
| HCAG_06308 | 40S ribosomal protein S12 | 3881,94 | 108,6785 | 3 |
| HCAG_08075 | 40S ribosomal protein S14 | 12754,87 | 190,4916 | 3 |
| HCAG_02704 | 40S ribosomal protein S15 | 14108,52 | 169,8381 | 3 |
| HCAG_08092 | 40S ribosomal protein S17 | 16364,66 | 276,7947 | 3 |
| HCAG_08667 | 40S ribosomal protein S18 | 10030,5 | 176,5375 | 3 |
| HCAG_02272 | 40S ribosomal protein S22 | 3728,225 | 222,6127 | 3 |
| HCAG_04418 | 40S ribosomal protein S24 | 3529,514 | 104,6506 | 3 |
| HCAG_02186 | 40S ribosomal protein S25 | 9014,106 | 187,2743 | 3 |
| HCAG_05528 | 40s ribosomal protein s26 | 14543,2 | 180,2246 | 3 |
| HCAG_01228 | 40S ribosomal protein S27 | 9230,458 | 210,9722 | 3 |
| HCAG_00214 | 40S ribosomal protein S3 | 13502,29 | 330,7447 | 3 |
| HCAG_06914 | 40S ribosomal protein S3A | 8963,706 | 268,4091 | 3 |
| HCAG_02430 | 40S ribosomal protein S5 A | 9329,065 | 270,826 | 3 |
| HCAG_06613 | 40S ribosomal protein S7e | 27823,93 | 288,2886 | 3 |
| HCAG_07249 | 40S ribosomal protein S9 | 9602,348 | 271,8529 | 3 |
| HCAG_01353 | 50S ribosomal protein L12 | 1917,47 | 40,2109 | 3 |
| HCAG_05884 | 6 phosphogluconate dehydrogenase | 8023,932 | 134,2552 | 3 |
| HCAG_02703 | 60S acidic ribosomal protein P2 | 37524,69 | 636,746 | 3 |
| HCAG_04231 | 60S ribosomal protein | 5466,812 | 184,5962 | 3 |
| HCAG_00468 | 60S ribosomal protein | 11604,62 | 332,2704 | 3 |
| HCAG_08351 | 60S ribosomal protein | 3132,571 | 95,03205 | 2 |
| HCAG_01850 | 60S ribosomal protein L1 | 4310,316 | 202,4637 | 3 |
| HCAG_04788 | 60S ribosomal protein L12 | 10017,52 | 96,46805 | 2 |
| HCAG_07708 | 60S ribosomal protein L13 | 5848,991 | 159,9491 | 3 |
| HCAG_03611 | 60S ribosomal protein L15 | 3859,495 | 180,8195 | 3 |
| HCAG_00164 | 60S ribosomal protein L16 | 3658,679 | 115,9205 | 3 |
| HCAG_02327 | 60S ribosomal protein L17 | 4335,706 | 131,8487 | 3 |
| HCAG_03593 | 60S ribosomal protein L18A | 6184,298 | 211,525 | 3 |
| HCAG_07408 | 60S ribosomal protein L19 | 630,1314 | 150,2913 | 3 |
| HCAG_08515 | 60S ribosomal protein L2 | 2675,752 | 190,8464 | 3 |
| HCAG_07248 | 60s ribosomal protein l21 | 9046,552 | 114,6985 | 3 |
| HCAG_04519 | 60S ribosomal protein L23 | 4206,323 | 146,5691 | 3 |
| HCAG_03167 | 60S ribosomal protein L24 | 3181,524 | 173,5972 | 3 |
| HCAG_00055 | 60S ribosomal protein L27 A | 9591,356 | 190,1584 | 3 |
| HCAG_03923 | 60S ribosomal protein L3 | 2166,346 | 190,2071 | 3 |
| HCAG_04561 | 60S ribosomal protein L34 | 3276,423 | 138,4565 | 2 |
| HCAG_03415 | 60S ribosomal protein L35 | 1572,399 | 167,4207 | 3 |
| HCAG_04185 | 60S ribosomal protein L36 | 5704,479 | 100,41 | 2 |
| HCAG_06534 | 60S ribosomal protein L43 | 2178,935 | 106,1251 | 2 |
| HCAG_07397 | 60S ribosomal protein L44 | 1394,489 | 51,6387 | 2 |
| HCAG_08444 | 60S ribosomal protein L5 | 20223,5 | 346,551 | 3 |
| HCAG_01793 | 60S ribosomal protein L6 | 6888,758 | 186,8992 | 3 |
| HCAG_08706 | 60S ribosomal protein L7 | 13876,5 | 296,9897 | 3 |
| HCAG_01606 | Acetate coa ligase | 629,007 | 42,84247 | 3 |
| HCAG_08039 | Acetoacetyl coa reductase | 436,767 | 32,5138 | 2 |
| HCAG_09712 | Acetoacetyl coa synthase | 438,9954 | 42,08695 | 2 |
| HCAG_06005 | Acetolactate synthase | 1168,337 | 64,7911 | 3 |
| HCAG_00814 | Acetyl coa acetyltransferase | 2630,681 | 70,0264 | 3 |
| HCAG_08621 | Acetyl coa acetyltransferase | 1335,855 | 43,06653 | 3 |
| HCAG_03801 | Acetylornithine aminotransferase | 703,3643 | 16,45323 | 3 |
| HCAG_05266 | Aconitase | 4464,713 | 211,8914 | 3 |
| HCAG_05531 | Aconitate hydratase | 252,6619 | 14,73045 | 2 |
| HCAG_08210 | Actin | 14794,47 | 561,8375 | 3 |
| HCAG_09978 | Acyl coa dehydrogenase | 1069,405 | 74,9761 | 3 |
| HCAG_09977 | Acyl coa dehydrogenase | 2132,312 | 40,54766 | 3 |
| HCAG_08512 | Adenosine kinase | 1205,733 | 51,25657 | 3 |
| HCAG_10265 | Adenosylhomocysteinase | 13278,38 | 150,7717 | 3 |
| HCAG_06743 | Adenylosuccinate synthetase adb | 1471,27 | 43,9758 | 3 |
| HCAG_06283 | Adp ATP carrier protein | 1485,426 | 79,02267 | 3 |
| HCAG_01847 | ADP ribosylation factor | 9345,842 | 212,8537 | 3 |
| HCAG_01583 | Aha1 domain family | 4458,064 | 137,6216 | 3 |
| HCAG_05679 | Alanine transaminase | 847,1642 | 53,8278 | 3 |
| HCAG_08561 | Alcohol dehydrogenase | 9589,18 | 315,7762 | 3 |
| HCAG_07199 | Aldehyde reductase I | 3414,787 | 60,97943 | 3 |
| HCAG_03188 | Allergen Asp F3 | 4953,224 | 87,64917 | 3 |
| HCAG_01535 | Alpha-ketoglutarate dehydrogenase | 730,2918 | 76,87164 | 3 |
| HCAG_03721 | Alternative oxidase | 450,1994 | 27,2548 | 3 |
| HCAG_06935 | Aminopeptidase | 736,5375 | 59,70234 | 3 |
| HCAG_07083 | Aminopeptidase | 874,8786 | 58,72897 | 3 |
| HCAG_08796 | Ankyrin repeat protein | 807,6809 | 31,48707 | 3 |
| HCAG_05224 | Anthranilate synthase component I | 2180,698 | 82,39523 | 3 |
| HCAG_00748 | Anthranilate synthase multifunctional enzyme | 610,8798 | 42,9185 | 2 |
| HCAG_00035 | Arginase | 3692,53 | 98,96786 | 3 |
| HCAG_07805 | Argininosuccinate lyase | 588,2655 | 33,0729 | 3 |
| HCAG_06683 | Argininosuccinate synthetase | 3385,939 | 111,2182 | 3 |
| HCAG_03866 | Arginyl trna synthetase | 465,6223 | 22,14395 | 2 |
| HCAG_06610 | Arp2 3 complex subunit | 2635,247 | 27,0049 | 3 |
| HCAG_03174 | Arp2 3 complex subunit | 1452,443 | 40,97177 | 3 |
| HCAG_04706 | ARP2 3 complex subunit | 1985,061 | 38,9795 | 3 |
| HCAG_00848 | Arp2 3 complex subunit | 862,9192 | 57,70245 | 2 |
| HCAG_00048 | Arsenical pump driving atpase | 1086,852 | 42,48613 | 3 |
| HCAG_08678 | Aspartate aminotransferase | 7898,256 | 209,2698 | 3 |
| HCAG_06102 | Aspartate aminotransferase | 2343,15 | 168,5527 | 3 |
| HCAG_10740 | Aspartyl trna synthetase | 948,9213 | 40,8965 | 3 |
| HCAG_04933 | ATP citrate lyase | 2139,029 | 126,857 | 3 |
| HCAG_04934 | ATP citrate synthase subunit 1 | 2099,132 | 125,1271 | 3 |
| HCAG_04273 | ATP dependent RNA helicase eif4a | 13675,61 | 169,9696 | 3 |
| HCAG_00406 | ATP dependent RNA helicase FAL1 | 1752,275 | 24,93793 | 3 |
| HCAG_05061 | ATP dependent RNA helicase SUB2 | 1386,906 | 69,001 | 2 |
| HCAG_06360 | ATP phosphoribosyltransferase | 1510,121 | 23,1415 | 3 |
| HCAG_05098 | ATP synthase D chain | 1066,909 | 32,02633 | 3 |
| HCAG_04799 | ATP synthase gamma chain | 4248,335 | 88,31953 | 3 |
| HCAG_02813 | ATP synthase subunit alpha | 11027,9 | 291,3853 | 3 |
| HCAG_07132 | BAR domain containing protein | 2296,888 | 86,57837 | 3 |
| HCAG_04224 | Bifunctional purine biosynthesis protein ADE16 | 3788,508 | 104,0566 | 3 |
| HCAG_08945 | Branched chain amino acid aminotransferase | 2292,734 | 71,38987 | 3 |
| HCAG_04357 | C 1 tetrahydrofolate synthase | 760,8495 | 47,88474 | 3 |
| HCAG_00633 | Calcineurin catalytic subunit cnaa | 694,1954 | 54,5769 | 2 |
| HCAG_01325 | Calmodulin | 2490,928 | 96,09657 | 3 |
| HCAG_05488 | Camp dependent protein kinase regulatory subunit pkar | 456,1616 | 34,21005 | 2 |
| HCAG_06885 | Cap binding protein | 979,2518 | 33,30407 | 3 |
| HCAG_02700 | Carbamoyl phosphate synthase | 459,5179 | 33,41815 | 2 |
| HCAG_03790 | Carnitine acetyl transferase | 1091,015 | 76,2204 | 3 |
| HCAG_08064 | Catalase | 4067,683 | 230,0302 | 3 |
| HCAG_08345 | Cell cycle control protein | 14299,69 | 330,5042 | 3 |
| HCAG_02452 | Cell division cycle protein | 7047,104 | 209,7992 | 3 |
| HCAG_00290 | Chorismate mutase | 1438,515 | 57,32437 | 3 |
| HCAG_00486 | Chromatin assembly factor 1 subunit C | 423,9109 | 57,6112 | 2 |
| HCAG_06981 | Citrate synthase | 8841,988 | 162,3114 | 3 |
| HCAG_04225 | Clathrin heavy chain | 1076,35 | 46,22586 | 3 |
| HCAG_00337 | Cleavage and polyadenylation specificity factor | 1945,841 | 63,4969 | 3 |
| HCAG_05565 | Cobalamin independent methionine synthase meth D | 12005,81 | 288,699 | 3 |
| HCAG_06960 | Cofilin | 3211,355 | 64,28446 | 3 |
| HCAG_02022 | Conserved hypothetical protein | 6511,567 | 82,4698 | 2 |
| HCAG_08732 | Conserved hypothetical protein | 12375,45 | 273,9344 | 3 |
| HCAG_04397 | Conserved hypothetical protein | 21679,95 | 246,0669 | 3 |
| HCAG_05185 | Conserved hypothetical protein | 2402,85 | 35,173 | 3 |
| HCAG_05552 | Conserved hypothetical protein | 2736,279 | 30,4943 | 3 |
| HCAG_02975 | Conserved hypothetical protein | 5327,349 | 76,036 | 2 |
| HCAG_06099 | Conserved hypothetical protein | 2587,254 | 45,5759 | 2 |
| HCAG_06375 | Conserved hypothetical protein | 5863,75 | 117,6953 | 3 |
| HCAG_04326 | Conserved hypothetical protein | 1426,516 | 20,71615 | 2 |
| HCAG_04232 | Conserved hypothetical protein | 661,942 | 23,45335 | 2 |
| HCAG_04776 | Conserved hypothetical protein | 7365,263 | 70,3944 | 3 |
| HCAG_06999 | Conserved hypothetical protein | 1586,429 | 42,0455 | 3 |
| HCAG_00803 | Conserved hypothetical protein | 1014,806 | 48,5732 | 2 |
| HCAG_07305 | Conserved hypothetical protein | 1812,133 | 81,92648 | 3 |
| HCAG_03758 | Conserved hypothetical protein | 3542,272 | 72,69483 | 3 |
| HCAG_05402 | Conserved hypothetical protein | 782,006 | 20,99755 | 2 |
| HCAG_07444 | Conserved hypothetical protein | 796,3632 | 51,81615 | 2 |
| HCAG_07936 | Conserved hypothetical protein | 402,9731 | 17,72575 | 2 |
| HCAG_04294 | Conserved hypothetical protein | 1635,122 | 40,5165 | 3 |
| HCAG_02919 | Conserved hypothetical protein | 1475,796 | 38,66193 | 3 |
| HCAG_03731 | Conserved hypothetical protein | 486,2803 | 21,9114 | 2 |
| HCAG_01332 | Conserved hypothetical protein | 865,2297 | 63,6858 | 3 |
| HCAG_07970 | Conserved hypothetical protein | 749,9848 | 196,3398 | 2 |
| HCAG_08367 | Conserved hypothetical protein | 5687,869 | 200,473 | 3 |
| HCAG_11293 | Conserved hypothetical protein | 2968,91 | 137,4345 | 3 |
| HCAG_06889 | Conserved hypothetical protein | 860,8 | 92,82117 | 3 |
| HCAG_03851 | Conserved hypothetical protein | 641,6033 | 45,97615 | 2 |
| HCAG_08192 | Conserved hypothetical protein | 1165,078 | 30,16707 | 3 |
| HCAG_00005 | Conserved hypothetical protein | 1334,497 | 49,9807 | 3 |
| HCAG_00272 | Conserved hypothetical protein | 537,689 | 27,01877 | 3 |
| HCAG_08411 | Conserved hypothetical protein | 712,8118 | 39,27887 | 3 |
| HCAG_01854 | Conserved hypothetical protein | 1276,919 | 33,41745 | 2 |
| HCAG_01835 | CORD and CS domain containing protein | 1721,563 | 67,8534 | 3 |
| HCAG_04015 | Coronin like protein crn1 | 788,0389 | 39,12313 | 3 |
| HCAG_06523 | Curved DNA binding protein | 743,7593 | 60,45393 | 3 |
| HCAG_07434 | Cystathionine beta synthase | 872,2492 | 43,00005 | 2 |
| HCAG_00616 | Cysteinyl trna synthetase | 544,9019 | 31,11764 | 3 |
| HCAG_07539 | Cytochrome b2 | 516,258 | 29,4317 | 3 |
| HCAG_00437 | Cytochrome c oxidase chain VI | 2317,187 | 53,29557 | 3 |
| HCAG_09319 | Cytochrome c peroxidase | 9986,348 | 161,9501 | 3 |
| HCAG_07098 | Cytochrome c peroxidase | 2578,487 | 157,7366 | 3 |
| HCAG_03694 | Cytosolic large ribosomal subunit protein L30 | 17386,25 | 209,9541 | 3 |
| HCAG_03111 | D 3 phosphoglycerate dehydrogenase | 585,0975 | 44,67647 | 3 |
| HCAG_09000 | Delta 1 pyrroline 5 carboxylate dehydrogenase prnc | 638,442 | 38,0306 | 2 |
| HCAG_03972 | Dihydrolipoamide acetyltransferase component | 2097,536 | 109,8462 | 3 |
| HCAG_08825 | Dihydrolipoamide dehydrogenase | 7554,961 | 182,2048 | 3 |
| HCAG_03522 | Dihydrolipoamide succinyltransferase | 1227,547 | 86,82027 | 3 |
| HCAG_05596 | Diphosphomevalonate decarboxylase | 1528,222 | 36,60697 | 3 |
| HCAG_04527 | DNA damage checkpoint protein rad24 | 23285,01 | 336,7387 | 3 |
| HCAG_00176 | Dnaj and TPR domain containing protein | 382,6965 | 25,3293 | 2 |
| HCAG_05805 | DNAK molecular chaperone bipa | 8733,758 | 496,3055 | 3 |
| HCAG_02296 | DUF1014 domain containing protein | 814,4769 | 36,02855 | 2 |
| HCAG_07572 | DUF427 domain containing protein | 1445,851 | 106,7784 | 2 |
| HCAG_07065 | DUF757 domain containing protein | 3106,05 | 39,91034 | 3 |
| HCAG_06605 | EB1 protein | 532,5627 | 10,55585 | 2 |
| HCAG_03847 | Electron transfer flavoprotein alpha subunit | 2845,263 | 99,4398 | 3 |
| HCAG_02931 | Electron transfer flavoprotein beta subunit | 1222,015 | 46,4588 | 2 |
| HCAG_01917 | Elicitor protein | 4218,983 | 188,1623 | 3 |
| HCAG_01784 | Elongation factor 1 beta | 13244,9 | 335,0582 | 3 |
| HCAG_08236 | Elongation factor 1 gamma | 8342,179 | 186,442 | 3 |
| HCAG_05988 | Elongation factor 2 | 9235,812 | 288,1314 | 3 |
| HCAG_03444 | Elongation factor Tu | 4772,664 | 123,0337 | 3 |
| HCAG_08058 | Enoyl coa hydratase isomerase family protein | 2558,898 | 79,85535 | 2 |
| HCAG_04675 | Esterase D | 958,4604 | 51,2963 | 2 |
| HCAG_04139 | Eukaryotic phosphomannomutase | 2159,559 | 62,71287 | 3 |
| HCAG_04206 | Eukaryotic translation initiation factor 2 gamma subunit | 579,6935 | 70,98791 | 3 |
| HCAG_08183 | Eukaryotic translation initiation factor 3 | 935,377 | 26,77673 | 3 |
| HCAG_08080 | Eukaryotic translation initiation factor 3 | 915,1443 | 36,842 | 3 |
| HCAG_03286 | Eukaryotic translation initiation factor 3 | 839,155 | 60,46716 | 3 |
| HCAG_08704 | Eukaryotic translation initiation factor 3 | 490,8954 | 54,30233 | 3 |
| HCAG_04356 | Eukaryotic translation initiation factor 3 subunit 2 | 1470,13 | 39,9899 | 2 |
| HCAG_03353 | Eukaryotic translation initiation factor 3 subunit 3 | 607,9618 | 39,7938 | 2 |
| HCAG_00044 | Eukaryotic translation initiation factor 3 subunit 6 | 497,7496 | 34,3335 | 2 |
| HCAG_01956 | Eukaryotic translation initiation factor 3 subunit eifcf | 2380,531 | 33,0398 | 2 |
| HCAG_00413 | Farnesyl pyrophosphate synthetase | 3870,777 | 140,5544 | 3 |
| HCAG_07636 | Fatty acid synthase alpha subunit fasa | 531,4349 | 59,70647 | 3 |
| HCAG_07637 | Fatty acid synthase beta subunit dehydratase | 661,2315 | 79,15063 | 3 |
| HCAG_07461 | Fimbrin | 1394,655 | 41,03476 | 3 |
| HCAG_03748 | FK506 binding protein 1A | 851,8973 | 52,15435 | 2 |
| HCAG_07972 | FKBP type peptidyl prolyl isomerase putative | 3158,454 | 80,91674 | 3 |
| HCAG_00010 | Fructose 1 6 biphosphate aldolase | 17987,7 | 493,8215 | 3 |
| HCAG_03322 | Fructose 1 6 bisphosphatase | 6459,385 | 221,4372 | 3 |
| HCAG_08493 | Fumarate hydratase class II | 12334,48 | 280,1246 | 3 |
| HCAG_03323 | Fumarate reductase flavoprotein subunit | 3147,101 | 125,6672 | 3 |
| HCAG_07031 | G protein comlpex beta subunit cpcb | 12519,53 | 323,788 | 3 |
| HCAG_08290 | Gamma butyrobetaine dioxygenase | 413,738 | 66,9063 | 3 |
| HCAG_07464 | Gamma glutamyl phosphate reductase | 2626,118 | 54,23727 | 3 |
| HCAG_05711 | GDP mannose pyrophosphorylase A | 2208,004 | 57,9971 | 3 |
| HCAG_04088 | Glucosamine fructose 6 phosphate aminotransferase | 4868,685 | 196,312 | 3 |
| HCAG_08202 | Glucose 6 phosphate isomerase | 2576,587 | 109,3278 | 3 |
| HCAG_03543 | Glutamate carboxypeptidase | 1578,964 | 58,8445 | 3 |
| HCAG_05651 | Glutamate dehydrogenase | 1054,277 | 56,1146 | 3 |
| HCAG_09757 | Glutamate synthase | 672,6308 | 52,95097 | 3 |
| HCAG_05070 | Glutamine synthetase | 546,2783 | 27,6783 | 2 |
| HCAG_07028 | Glutamine synthetase partial | 1904,71 | 94,95274 | 3 |
| HCAG_08250 | Glutaminyl trna synthetase | 928,2912 | 30,02714 | 3 |
| HCAG_04910 | Glyceraldehyde 3 phosphate dehydrogenase | 61046,14 | 1224,222 | 3 |
| HCAG_03267 | Glycerol 3 phosphate dehydrogenase | 999,9523 | 57,46107 | 3 |
| HCAG_07571 | Glycine dehydrogenase | 1310,57 | 52,3631 | 3 |
| HCAG_02914 | Glycine rich protein | 12427,35 | 339,1012 | 3 |
| HCAG_03017 | Glycyl trna synthetase | 529,5927 | 52,32585 | 2 |
| HCAG_04496 | GMP synthase putative | 1505,514 | 44,72493 | 3 |
| HCAG_05187 | GTP binding nuclear protein GSP1 Ran | 17182,13 | 353,1788 | 3 |
| HCAG_06262 | GTP binding protein | 756,6933 | 22,661 | 2 |
| HCAG_06941 | GTP binding protein | 2024,781 | 56,8438 | 3 |
| HCAG_07659 | GTP binding protein | 1048,013 | 13,4244 | 3 |
| HCAG_01447 | GTP binding protein | 1126,126 | 54,9423 | 3 |
| HCAG_02007 | Guanylate kinase | 844,4261 | 41,26255 | 2 |
| HCAG_06977 | H atpase | 415,0656 | 26,9263 | 3 |
| HCAG_08408 | HAD superfamily hydrolase | 12974,51 | 428,0258 | 3 |
| HCAG_04686 | Heat shock protein | 44992,54 | 1193,477 | 3 |
| HCAG_04111 | Heat shock protein 30 | 3264,537 | 302,5589 | 3 |
| HCAG_00806 | Heat shock protein SSB1 | 3801,616 | 122,2351 | 3 |
| HCAG_08176 | Heat shock protein SSC1 | 17075,71 | 378,8485 | 3 |
| HCAG_04471 | Heat shock protein STI1 | 5382,831 | 171,4749 | 3 |
| HCAG_01131 | Het c2 protein | 1105,472 | 39,93617 | 3 |
| HCAG_02357 | Histidinol dehydrogenase | 659,5228 | 61,03953 | 3 |
| HCAG_08774 | Histone chaperone asf1 | 1486,036 | 53,0967 | 3 |
| HCAG_03524 | Histone H2A | 2912,565 | 188,7169 | 3 |
| HCAG_04914 | Histone H2A | 2026,588 | 19,91765 | 2 |
| HCAG_03525 | Histone H2b | 9974,005 | 270,4625 | 3 |
| HCAG_03885 | Histone h4 | 4158,552 | 116,1169 | 3 |
| HCAG_00066 | HNRNP arginine N methyltransferase | 605,7864 | 19,72835 | 2 |
| HCAG_06098 | Homocitrate synthase | 2684,061 | 80,16656 | 3 |
| HCAG_00014 | Homoserine dehydrogenase | 794,0332 | 58,155 | 2 |
| HCAG_04943 | Hsp10 like protein | 24012,22 | 568,8479 | 3 |
| HCAG_06961 | Hsp60 like protein | 41212,29 | 828,719 | 3 |
| HCAG_01398 | Hsp70 like protein | 56129,46 | 1049,763 | 3 |
| HCAG_00783 | Hsp88 like protein | 8559,245 | 298,9356 | 3 |
| HCAG_05533 | Hypothetical protein | 24388,81 | 337,0344 | 3 |
| HCAG_07506 | Hypothetical protein | 24090,81 | 157,9821 | 3 |
| HCAG_06895 | Hypothetical protein | 843,4086 | 80,78505 | 2 |
| HCAG_06361 | Hypothetical protein | 30146,46 | 204,525 | 3 |
| HCAG_04824 | Hypothetical protein | 2565,487 | 31,19303 | 3 |
| HCAG_06110 | Immunogenic protein | 8589,824 | 326,8529 | 3 |
| HCAG_04307 | Inorganic pyrophosphatase | 1248,939 | 64,13903 | 3 |
| HCAG_06694 | Isoleucyl trna synthetase cytoplasmic | 716,8983 | 55,70977 | 3 |
| HCAG_08890 | Ketol acid reductoisomerase | 14108,47 | 519,1456 | 3 |
| HCAG_06296 | Ketoreductase | 429,1789 | 38,9558 | 2 |
| HCAG_06295 | KH domain RNA binding protein | 4513,452 | 67,63947 | 3 |
| HCAG_03732 | KH domain RNA binding protein | 652,4767 | 37,76755 | 2 |
| HCAG_05229 | Leucyl trna synthetase | 445,6577 | 41,83935 | 2 |
| HCAG_07440 | Leukotriene A4 hydrolase | 548,4642 | 30,27205 | 2 |
| HCAG_01128 | LRP16 family protein | 537,8452 | 28,0498 | 2 |
| HCAG_05713 | Lysyl trna synthetase | 1356,735 | 71,04867 | 3 |
| HCAG_06901 | Malate dehydrogenase | 16307,27 | 213,0977 | 3 |
| HCAG_03969 | Malate dehydrogenase | 37909,94 | 509,3897 | 3 |
| HCAG_05084 | Malate synthase | 461,0543 | 39,04725 | 2 |
| HCAG_03448 | Manganese superoxide dismutase | 7937,047 | 307,6306 | 3 |
| HCAG_08720 | Mannitol 1 phosphate dehydrogenase | 8214,13 | 249,4394 | 3 |
| HCAG_01552 | Mannose 1 phosphate guanyltransferase | 1321,119 | 63,20145 | 2 |
| HCAG_08436 | Mannose 6 phosphate isomerase | 926,756 | 30,90103 | 3 |
| HCAG_06059 | Methylmalonate semialdehyde dehydrogenase | 1703,577 | 132,7407 | 3 |
| HCAG_05584 | Mitochondrial acetolactate synthase small subunit | 847,1709 | 38,43224 | 3 |
| HCAG_06944 | Mitochondrial ATP synthase | 14512,35 | 259,4264 | 3 |
| HCAG_00973 | Mitochondrial F1F0 ATP synthase subunit F | 2760,902 | 43,1316 | 3 |
| HCAG_02342 | Mitochondrial processing peptidase subunit | 627,1706 | 62,89457 | 3 |
| HCAG_04853 | Mitochondrial protein import protein MAS5 | 2242,823 | 34,81233 | 3 |
| HCAG_00893 | Molybdopterin binding domain containing protein | 856,8976 | 37,4294 | 2 |
| HCAG_08291 | Monothiol glutaredoxin 4 | 494,8101 | 23,3259 | 2 |
| HCAG_04339 | Multi bridging factor 1 putative | 3816,756 | 42,63723 | 3 |
| HCAG_00064 | N acetylglucosamine phosphate mutase | 3589,48 | 92,26431 | 3 |
| HCAG_04093 | NAD isocitrate dehydrogenase subunit I | 3115,712 | 44,80767 | 3 |
| HCAG_03650 | NAD specific glutamate dehydrogenase | 702,092 | 95,95243 | 3 |
| HCAG_05099 | Nascent polypeptide associated complex subunit alpha | 8742,293 | 230,9036 | 3 |
| HCAG_05051 | Nascent polypeptide associated complex subunit beta | 11732,94 | 114,9328 | 3 |
| HCAG_03497 | Nicotinate nucleotide pyrophosphorylase | 4568,414 | 122,3166 | 3 |
| HCAG_09244 | Nuclear and cytoplasmic polyadenylated RNA binding protein pub1 | 1824,574 | 52,68423 | 3 |
| HCAG_02807 | Nucleic acid binding protein | 802,1064 | 46,19006 | 3 |
| HCAG_00544 | Nucleoside diphosphate kinase | 10785 | 238,5037 | 3 |
| HCAG_03106 | Nucleosome assembly protein | 2964,14 | 122,8346 | 3 |
| HCAG_00018 | Nucleosome binding protein | 1014,286 | 69,39923 | 3 |
| HCAG_00907 | NUDIX hydrolase | 1056,262 | 34,88947 | 3 |
| HCAG_07004 | O acetylhomoserine | 578,2699 | 19,93255 | 2 |
| HCAG_02754 | Oligopeptidase family protein | 934,8282 | 45,3226 | 2 |
| HCAG_00889 | Oxidoreductase | 2357,371 | 50,3971 | 2 |
| HCAG_08156 | Oxidoreductase | 747,5274 | 15,92415 | 2 |
| HCAG_08190 | Oxidoreductase 2 nitropropane dioxygenase | 998,795 | 31,17535 | 2 |
| HCAG_06409 | PCI domain containing protein | 656,4379 | 50,5613 | 2 |
| HCAG_04544 | Peptide methionine sulfoxide reductase | 1278,386 | 82,35105 | 2 |
| HCAG_07345 | Peptidyl prolyl cis trans isomerase | 2320,891 | 124,7127 | 3 |
| HCAG_08833 | Peptidyl prolyl cis trans isomerase | 22116,2 | 489,2655 | 3 |
| HCAG_04215 | Peptidyl prolyl cis trans isomerase B | 2465,546 | 60,22563 | 3 |
| HCAG_04485 | Peptidylprolyl isomerase | 17467,85 | 852,0681 | 3 |
| HCAG_04358 | Peroxisomal NADP dependent isocitrate dehydrogenase | 783,0504 | 38,19657 | 3 |
| HCAG_06371 | Phenylacetyl coa ligase | 426,9908 | 12,5269 | 2 |
| HCAG_04499 | Phenylalanyl trna synthetase | 681,1558 | 26,26685 | 2 |
| HCAG_00215 | Phenylalanyl trna synthetase | 546,4713 | 39,0176 | 2 |
| HCAG_03270 | Phospho 2 dehydro 3 deoxyheptonate aldolase | 1155,334 | 46,3055 | 2 |
| HCAG_05681 | Phosphoenolpyruvate carboxykinase acuf | 2235,247 | 161,563 | 3 |
| HCAG_07552 | Phosphofructokinase | 611,8942 | 31,87707 | 3 |
| HCAG_08808 | Phosphoglucomutase | 1855,729 | 102,0644 | 3 |
| HCAG_03385 | Phosphoglycerate kinase | 12423,89 | 365,3285 | 3 |
| HCAG_03809 | Phosphoribosylaminoimidazole carboxylase | 432,9993 | 14,89755 | 2 |
| HCAG_06026 | Polyadenylate binding protein | 2824,612 | 115,6125 | 3 |
| HCAG_11185 | Pre mrna processing protein | 378,269 | 17,36665 | 2 |
| HCAG_08343 | Predicted protein | 5790,637 | 66,44477 | 3 |
| HCAG_10780 | Predicted protein | 7184,29 | 99,97333 | 3 |
| HCAG_01399 | Predicted protein | 1456,296 | 95,82465 | 2 |
| HCAG_10781 | Predicted protein | 2243,416 | 90,98335 | 2 |
| HCAG_06996 | Processing enhancing protein | 1129,767 | 74,94576 | 3 |
| HCAG_08950 | Progesterone binding protein | 1655,529 | 61,9584 | 2 |
| HCAG_04835 | Proliferating cell nuclear antigen | 9254,571 | 294,8607 | 3 |
| HCAG_07283 | Prolyl trna synthetase | 1130,52 | 60,4771 | 3 |
| HCAG_04190 | Proteasome component | 2027,552 | 51,2532 | 2 |
| HCAG_04101 | Proteasome component | 3194,46 | 77,30953 | 3 |
| HCAG_00347 | Proteasome component | 3650,723 | 65,82407 | 3 |
| HCAG_04090 | Proteasome component | 424,4596 | 43,82935 | 2 |
| HCAG_03939 | Proteasome component | 1305,804 | 56,22657 | 3 |
| HCAG_05910 | Proteasome component | 718,4679 | 37,51605 | 2 |
| HCAG_00053 | Proteasome subunit alpha | 1034,737 | 59,99005 | 2 |
| HCAG_03630 | Proteindisulfidisomerase | 10252,34 | 477,6328 | 3 |
| HCAG_08383 | Psi protein | 884,1674 | 24,71737 | 3 |
| HCAG_02994 | Pyridoxine biosynthesis protein pyroa validated | 8497,033 | 129,6322 | 3 |
| HCAG_04227 | Pyruvate carboxylase | 1902,689 | 80,9469 | 3 |
| HCAG_08778 | Pyruvate decarboxylase | 1304,702 | 77,70216 | 3 |
| HCAG_01360 | Pyruvate dehydrogenase E1 component alpha subunit | 2457,061 | 106,0257 | 3 |
| HCAG_07619 | Pyruvate dehydrogenase E1 component beta subunit | 6548,242 | 121,4311 | 3 |
| HCAG_07781 | Pyruvate kinase | 3530,522 | 71,90674 | 3 |
| HCAG_02612 | Ran specific gtpase activating protein | 3245,281 | 115,1822 | 3 |
| HCAG_05560 | Rho gtpase | 670,144 | 21,85075 | 2 |
| HCAG_04939 | Ribose 5 phosphate isomerase A | 1260,122 | 25,7107 | 2 |
| HCAG_03695 | Ribosomal L10 protein | 8968,191 | 241,6503 | 3 |
| HCAG_03504 | Ribosomal protein L22 | 6314,293 | 163,6783 | 3 |
| HCAG_04987 | Ribosomal protein L22e | 5621,855 | 205,9209 | 3 |
| HCAG_03055 | Ribosomal protein L23a | 2837,255 | 96,8363 | 3 |
| HCAG_05192 | Ribosomal protein L31e | 5415,667 | 164,4855 | 3 |
| HCAG_06425 | Ribosomal protein L32 | 10957,93 | 247,8808 | 3 |
| HCAG_05221 | Ribosomal protein L7a | 11324,91 | 257,9532 | 3 |
| HCAG_04856 | Ribosomal protein P0 | 7256,87 | 229,6384 | 3 |
| HCAG_04662 | Ribosomal protein S13 | 8840,399 | 204,3448 | 3 |
| HCAG_04575 | Ribosomal protein S16 | 11695,7 | 229,2052 | 3 |
| HCAG_08821 | Ribosomal protein S20 | 7590,177 | 185,2737 | 3 |
| HCAG_04498 | Ribosomal protein S21e | 6717,14 | 225,7652 | 3 |
| HCAG_01947 | Ribosomal protein S23 | 2556,498 | 109,1607 | 3 |
| HCAG_07237 | Ribosomal protein S4 | 7116,837 | 291,4173 | 3 |
| HCAG_07961 | Ribosomal protein S5 | 9939,736 | 278,6216 | 3 |
| HCAG_01666 | Ribosomal protein S6 | 3579,4 | 111,0932 | 3 |
| HCAG_08073 | Ribosomal protein S9 | 12215,21 | 196,0245 | 3 |
| HCAG_08342 | RNA binding domain containing protein | 643,9538 | 53,3616 | 2 |
| HCAG_04410 | RNA binding protein a | 3201,677 | 131,2424 | 3 |
| HCAG_04664 | RNA recognition domain containing protein family protein | 685,8578 | 80,6694 | 2 |
| HCAG_00597 | Rnase III domain containing protein | 497,9128 | 18,30715 | 2 |
| HCAG_05306 | RNP domain containing protein | 2965,543 | 109,1367 | 3 |
| HCAG_07930 | RPEL repeat protein | 2199,187 | 66,3206 | 2 |
| HCAG_02082 | Ruvb like helicase | 443,3555 | 17,0074 | 2 |
| HCAG_03428 | S adenosylmethionine synthetase | 3760,825 | 105,5239 | 3 |
| HCAG_01145 | Saccharopine dehydrogenase | 1205,061 | 50,8569 | 3 |
| HCAG_01575 | Saccharopine reductase | 2364,546 | 89,05029 | 3 |
| HCAG_05465 | Sec14 cytosolic factor | 580,8625 | 53,7352 | 3 |
| HCAG_07830 | Secretory pathway gdp dissociation inhibitor | 2641,345 | 94,14173 | 3 |
| HCAG_03836 | Septin | 1483,816 | 55,62654 | 3 |
| HCAG_00717 | Septin 1 | 2141,66 | 78,24886 | 3 |
| HCAG_00459 | Septin 2 | 849,1599 | 59,23857 | 3 |
| HCAG_02006 | Septin 3 | 1173,765 | 60,53613 | 3 |
| HCAG_05787 | Ser Thr protein phosphatase | 861,5215 | 54,8059 | 3 |
| HCAG_05408 | Serine hydroxymethyltransferase | 4116,238 | 86,99223 | 3 |
| HCAG_07418 | Serine hydroxymethyltransferase | 2603,516 | 139,9004 | 3 |
| HCAG_08377 | Serine threonine phosphatase | 754,6139 | 41,1204 | 2 |
| HCAG_03112 | Seryl trna synthetase | 750,4748 | 49,58113 | 3 |
| HCAG_05071 | Short chain dehydrogenase | 3996,884 | 91,6941 | 3 |
| HCAG_04840 | Small COPII coat gtpase sar1 | 2362,336 | 50,18705 | 2 |
| HCAG_03703 | Small nuclear ribonucleoprotein Sm D1 | 871,0995 | 65,3369 | 2 |
| HCAG_05252 | Snare sec24 | 456,5576 | 26,563 | 2 |
| HCAG_04999 | Spermidine synthase | 5750,292 | 223,8345 | 3 |
| HCAG_03646 | Stress protein p66 | 681,952 | 38,275 | 3 |
| HCAG_03348 | Stress responsive A B barrel domain containing protein | 595,9839 | 20,58975 | 2 |
| HCAG_10301 | Suaprga1 | 2760,1 | 75,11964 | 3 |
| HCAG_06317 | Succinate dehydrogenase | 1476,041 | 66,64664 | 3 |
| HCAG_03263 | Succinate dehydrogenase iron sulfur protein | 491,2531 | 63,9407 | 2 |
| HCAG_08548 | Succinyl coa ligase alpha chain | 3350,317 | 56,04593 | 3 |
| HCAG_07697 | Succinyl coa ligase beta chain | 3804,913 | 83,8985 | 3 |
| HCAG_03008 | Sulfur metabolite repression control protein | 13253,11 | 101,0936 | 3 |
| HCAG_06628 | T complex protein | 1221,959 | 44,8042 | 2 |
| HCAG_07282 | T complex protein | 724,0911 | 36,38823 | 3 |
| HCAG_05697 | T complex protein 1 epsilon subunit | 1530,83 | 50,40176 | 3 |
| HCAG_03305 | T complex protein 1 subunit beta | 1074,614 | 45,73463 | 3 |
| HCAG_06315 | T complex protein 1 subunit delta | 1221,563 | 45,73345 | 2 |
| HCAG_05525 | T complex protein 1 subunit eta | 669,0792 | 25,61325 | 2 |
| HCAG_02143 | TCTP family protein | 10430,09 | 181,3029 | 3 |
| HCAG_06210 | Thiol specific antioxidant | 25448,88 | 350,1167 | 3 |
| HCAG_00878 | Thioredoxin domain containing protein | 32294,1 | 216,23 | 3 |
| HCAG_07096 | Thioredoxin domain containing protein | 1237,599 | 29,8413 | 2 |
| HCAG_07622 | Trans 2 enoyl coa reductase | 895,5127 | 10,68463 | 3 |
| HCAG_00638 | Transaldolase | 1522,537 | 134,8891 | 3 |
| HCAG_02611 | Transcription initiation factor TFIID subunit 14 | 1231,008 | 56,56213 | 3 |
| HCAG_05000 | Transketolase tkta | 2617,731 | 92,5933 | 3 |
| HCAG_08798 | Translation elongation factor 1 alpha | 20291,22 | 1078,696 | 3 |
| HCAG_01178 | Translation initiation factor 3 | 5862,111 | 43,7767 | 3 |
| HCAG_06021 | Translation initiation factor eif 5A putative | 1865,138 | 80,9669 | 3 |
| HCAG_00267 | Translation initiation factor eif3 | 539,4661 | 25,33065 | 2 |
| HCAG_03569 | Translation initiation factor eif3a | 827,1584 | 59,45277 | 3 |
| HCAG_02511 | Triosephosphate isomerase | 13003,76 | 275,6922 | 3 |
| HCAG_01469 | Trna ligase | 210,0928 | 0 | 2 |
| HCAG_05882 | Tryptophanyl trna synthetase | 1006,899 | 38,7007 | 3 |
| HCAG_02068 | Tubulin alpha 1 subunit | 1251,869 | 27,1183 | 2 |
| HCAG_01781 | Tubulin beta chain | 1742,919 | 83,145 | 3 |
| HCAG_08288 | Tubulin subunit alpha 2 | 3343,532 | 83,12547 | 3 |
| HCAG_06019 | Ubiquitin | 15953,08 | 0 | 2 |
| HCAG_09628 | Ubiquitin activating enzyme E1 | 1635,299 | 60,11043 | 3 |
| HCAG_06566 | Ubiquitin fusion protein | 15773,92 | 628,3005 | 3 |
| HCAG_01770 | Ubiquitin like modifier SUMO | 1110,976 | 97,10547 | 3 |
| HCAG_06641 | UDP galactopyranose mutase | 1034,324 | 88,43106 | 3 |
| HCAG_09614 | UDP glucose 4 epimerase | 670,2289 | 37,5002 | 3 |
| HCAG_09613 | UDP glucose 4 epimerase Gal10 | 576,424 | 47,69055 | 2 |
| HCAG_04416 | UDP N acetylglucosamine pyrophosphorylase | 4410,609 | 101,0834 | 3 |
| HCAG_00404 | Vacuolar ATP synthase catalytic subunit A | 1729,485 | 71,71136 | 3 |
| HCAG_05951 | Vacuolar ATP synthase subunit B | 543,7919 | 37,8907 | 2 |
| HCAG_06619 | Vacuolar sorting associated protein | 1031,433 | 25,4114 | 2 |
| HCAG_10092 | Valyl trna synthetase | 444,0311 | 35,13225 | 2 |
| HCAG_07700 | Xanthine phosphoribosyltransferase | 2093,559 | 38,55233 | 3 |
| HCAG_07567 | Zuotin | 661,366 | 28,43987 | 3 |

^a^ Identification of proteins from *Histoplasma* genome database using the ProteinLynx Global Server vs. 2.4 (PLGS) (Waters Corporation, Manchester, UK) (http://www.broadinstitute.org/annotation/genome/histoplasma_capsulatum/MultiHome.html).

^b^ Genes annotation from *Histoplasma* genome database or by homology from NCBI database (http://www.ncbi.nlm.nih.gov/).
