# Supplementary material for: Comparative Proteomic Analysis of Histoplasma capsulatum Yeast and Mycelium Reveals Differential Metabolic Shifts and Cell Wall Remodeling Processes in the Different Morphotypes
Source: Front Microbiol. 2021 Jun 11;12:640931. doi: 10.3389/fmicb.2021.640931 (PMC8226243; doi:10.3389/fmicb.2021.640931)
Supplement: Supplementary Table 3 — Identified proteins from Histoplasma capsulatum up-regulated in mycelium compared to yeast cells. [file Table_3.DOCX]

**Supplementary Table 3: Identified proteins from *Histoplasma capsulatum* up-regulated in mycelium compared to yeast cells**

| **Accession number^a^** | **Protein Description^b^** | **Score** | **Ratio Mycelium/Yeast^c^** |
| --- | --- | --- | --- |
| **Functional categories^d^** | |  |  |
| **1. METABOLISM** |  |  |  |
| **Amino acid metabolism** |  |  |  |
| HCAG_05761 | 2-oxoisovalerate dehydrogenase alpha subunit | 660,06 | 1,682027618 |
| HCAG_02751 | 3-hydroxyanthranilate 3,4-dioxygenase | 382,66 | * |
| HCAG_00678 | 3-methylcrotonyl-coa carboxylase biotin containing subunit | 963,26 | * |
| HCAG_08375 | 4-aminobutyrate aminotransferase | 2906,55 | * |
| HCAG_05363 | 5-oxoprolinase | 1227,86 | * |
| HCAG_06005 | Acetolactate synthase | 1251,86 | 3,525421454 |
| HCAG_04183 | Acetylornithine deacetylase | 479,28 | * |
| HCAG_10265 | Adenosylhomocysteinase | 12486,09 | 1,632316236 |
| HCAG_04617 | Aminotransferase | 789,41 | * |
| HCAG_05962 | Hercynylcysteine S-oxide lyase | 455,46 | * |
| HCAG_06360 | ATP phosphoribosyltransferase | 1513,2 | 1,50681778 |
| HCAG_03336 | Branched chain alpha keto acid dehydrogenase E1 subunit beta | 582 | 2,773194711 |
| HCAG_03302 | Branched chain amino acid aminotransferase | 539,16 | 5,259310669 |
| HCAG_01864 | Copper amine oxidase | 846,39 | * |
| HCAG_04455 | Cystathionine gamma synthase | 383,65 | * |
| HCAG_01780 | Delta-1-pyrroline-5-carboxylate dehydrogenase prnc | 1332,85 | * |
| HCAG_09000 | Delta-1-pyrroline-5-carboxylate dehydrogenase prnc | 758,52 | 2,611696417 |
| HCAG_07219 | Dihydrodipicolinate synthetase family protein | 809,25 | * |
| HCAG_02120 | Fumarylacetoacetase hydrolase family protein | 794,17 | 4,572225108 |
| HCAG_08662 | Glutamate decarboxylase | 785,29 | * |
| HCAG_03455 | Glycine cleavage system H protein | 700,03 | * |
| HCAG_05721 | Homogentisate 1,2-dioxygenase | 1418,7 | * |
| HCAG_03301 | Homoserine acetyltransferase | 502,76 | * |
| HCAG_02035 | Imidazole glycerol phosphate synthase hishf | 418,79 | * |
| HCAG_04432 | Indoleamine 2,3-dioxygenase family protein | 2149,66 | * |
| HCAG_00677 | Isovaleryl-coa dehydrogenase | 1677,65 | * |
| HCAG_08086 | Kynureninase | 586,87 | * |
| HCAG_01655 | Kynurenine-oxoglutarate transaminase | 673,6 | * |
| HCAG_00676 | Methylcrotonoyl-coa carboxylase beta chain | 832,9 | * |
| HCAG_06059 | Methylmalonate semialdehyde dehydrogenase | 1835,48 | 4,48168907 |
| HCAG_05540 | N alpha acyl glutamine aminoacylase | 663,15 | * |
| HCAG_00397 | NAD dependent epimerase dehydratase family protein | 513,19 | * |
| HCAG_07004 | O-acetylhomoserine | 458,61 | 1,896480852 |
| HCAG_00575 | O-methyltransferase | 686,02 | * |
| HCAG_08313 | Oxidoreductase | 742,68 | * |
| HCAG_00889 | Oxidoreductase | 2497,77 | 3,421229602 |
| HCAG_08156 | Oxidoreductase | 752,56 | 1,750672504 |
| HCAG_06371 | Phenylacetyl-coa ligase | 391,44 | 1,750672504 |
| HCAG_01145 | Saccharopine dehydrogenase | 1217,23 | 1,973877746 |
| HCAG_01575 | Saccharopine reductase | 2729,37 | 1,616074385 |
| HCAG_07261 | Succinate semialdehyde dehydrogenase | 1092,66 | * |
| HCAG_00793 | Threonine synthase | 1392,28 | * |
| HCAG_00067 | Trimethyllysine dioxygenase | 379,18 | 4,526730751 |
| HCAG_04336 | Tyrosinase | 472,51 | * |
|  |  |  |  |
| **Nitrogen, sulfur and selenium metabolism** | |  |  |
| HCAG_04927 | Acetamidase | 531,52 | * |
| HCAG_08831 | Formamidase | 20186,97 | * |
| HCAG_07200 | Nitrate reductase | 1184 | * |
| HCAG_07558 | Nmra like family domain containing protein | 1047,36 | * |
| HCAG_00429 | Oxidoreductase | 1929,25 | * |
| HCAG_08190 | Oxidoreductase 2-nitropropane dioxygenase | 1136,96 | 2,664456293 |
|  |  |  |  |
| **Nucleotide metabolism** |  |  |  |
| HCAG_08512 | Adenosine kinase | 980,35 | 2,316366916 |
| HCAG_01433 | Adenylosuccinate lyase | 794,53 | * |
| HCAG_06648 | Allantoicase | 569,75 | * |
| HCAG_01893 | Cytidine deaminase putative | 514,25 | * |
| HCAG_05757 | Endonuclease exonuclease phosphatase family protein | 1470,66 | * |
| HCAG_01212 | Sulfate adenylyltransferase | 849,88 | * |
| HCAG_03261 | Thiamine biosynthesis protein | 1801,89 | * |
| HCAG_06443 | Uracil phosphoribosyltransferase | 678,1 | * |
|  |  |  |  |
| **Phosphate metabolism** |  |  |  |
| HCAG_00980 | Alkaline phosphatase | 1272,91 | * |
| HCAG_05184 | Stress protein DDR48 | 2015,61 | * |
|  |  |  |  |
| **C-compound and carbohydrate metabolism** | |  |  |
| HCAG_05094 | 2-methylcitrate dehydratase | 9072,32 | 1,537257535 |
| HCAG_05090 | 2-methylcitrate synthase | 4778,23 | 2,585709628 |
| HCAG_01606 | Acetil-CoA ligase | 508,35 | 7,028687916 |
| HCAG_08670 | Glucose methanol choline oxidoreductase | 803,39 | * |
| HCAG_00626 | L xylulose reductase | 7307,77 | * |
| HCAG_01368 | Lactoylglutathione lyase | 2361,47 | * |
| HCAG_08449 | Mannosyl-oligosaccharide 1,2-alpha-mannosidase | 382,89 | * |
| HCAG_05089 | Methylisocitrate lyase | 392,94 | 2,013752683 |
| HCAG_11124 | NAD dependent formate dehydrogenase acia Fdh | 8004,9 | * |
| HCAG_09102 | NADP D xylose dehydrogenase | 919,71 | * |
| HCAG_03605 | NADP dependent mannitol dehydrogenase | 27375,1 | * |
| HCAG_02919 | Nucleoside-diphosphate-sugar epimerase | 1870,55 | 6,049647176 |
| HCAG_06234 | PEP phosphonomutase | 890,1 | * |
| HCAG_07680 | Ribitol kinase | 440,79 | * |
| HCAG_07206 | S-(hydroxymethyl)glutathione dehydrogenase | 405,9 | 2,181472203 |
| HCAG_05311 | Succinyl-coa 3-ketoacid coenzyme A transferase | 997,27 | * |
| HCAG_09614 | UDP-glucose 4-epimerase | 675,05 | 1,552707215 |
| HCAG_04745 | Y20 protein | 39494,69 | * |
|  |  |  |  |
| **Lipid, fatty acid and isoprenoid metabolism** | |  |  |
| HCAG_01063 | 3-oxoacyl acyl-carrier-protein reductase | 1329,7 | * |
| HCAG_00814 | Acetyl-coa acetyltransferase | 2749,15 | 1,552707215 |
| HCAG_08621 | Acetyl-coa acetyltransferase | 1229,88 | 1,568312167 |
| HCAG_01711 | Alpha beta hydrolase | 466,82 | * |
| HCAG_03184 | Cytochrome P450 oxidoreductase | 463,19 | * |
| HCAG_02555 | Dihydroxyacetone kinase | 475,11 | * |
| HCAG_11321 | FAD dependent oxidoreductase | 659,44 | * |
| HCAG_02303 | Fatty acid amide hydrolase | 681,63 | * |
| HCAG_08471 | Geranylgeranyl pyrophosphate synthase | 436,05 | * |
| HCAG_03015 | Phytanoyl-coa dioxygenase | 846,06 | * |
| HCAG_00118 | Short chain dehydrogenase reductase | 1093,78 | * |
| HCAG_04443 | Short chain dehydrogenase reductase | 3040,14 | * |
| HCAG_07776 | Sphingolipid long chain base responsive protein PIL1 | 384,5 | * |
|  |  |  |  |
| **Metabolism of vitamins, cofactors, and prosthetic groups** | |  |  |
| HCAG_04089 | 6,7-dimethyl-8-ribityllumazine synthase | 463,56 | * |
| HCAG_02662 | Porphobilinogen deaminase | 640,28 | * |
| HCAG_05915 | Thiazole biosynthetic enzyme | 6809,64 | * |
|  |  |  |  |
| **Secondary metabolism** |  |  |  |
| HCAG_06504 | 3-demethylubiquinone-9 3-methyltransferase | 2161,76 | * |
| HCAG_07305 | Isochorismatase domain-containing protein | 1899,44 | 1,584073998 |
| HCAG_04454 | Molybdopterin synthase small subunit cnxg | 683,45 | * |
| HCAG_07761 | Phenazine biosynthesis phzc phzf protein | 319,59 | * |
| HCAG_06128 | Thij pfpi family protein | 5487,97 | * |
|  |  |  |  |
| **2. ENERGY** |  |  |  |
| **Glycolysis and gluconeogenesis** | |  |  |
| HCAG_05959 | Alcohol dehydrogenase | 566,41 | * |
| HCAG_00576 | Alcohol dehydrogenase | 422,79 | * |
| HCAG_01578 | Alcohol dehydrogenase | 1014,47 | * |
| HCAG_02317 | Alcohol dehydrogenase | 1841,2 | * |
| HCAG_05083 | Alcohol dehydrogenase | 573,72 | * |
| HCAG_06397 | Alcohol dehydrogenase | 3336,29 | * |
| HCAG_08367 | Aldehyde dehydrogenase | 5659,57 | 3,632786417 |
| HCAG_06205 | Aldehyde dehydrogenase | 1321,11 | * |
| HCAG_02371 | Aldehyde dehydrogenase | 9816,14 | * |
| HCAG_08789 | Fructose-bisphosphate aldolase | 487,44 | * |
| HCAG_08202 | Glucose-6-phosphate isomerase | 3345,96 | 1,584073998 |
| HCAG_08447 | Glycogen phosphorylase | 443,75 | * |
| HCAG_03661 | Hexokinase | 998,21 | * |
| HCAG_07781 | Pyruvate kinase | 3453,42 | 1,616074385 |
|  |  |  |  |
| **Glyoxylate cycle** |  |  |  |
| HCAG_05266 | Aconitase | 4771,98 | 1,616074385 |
| HCAG_10958 | Isocitrate lyase | 1771,38 | 2,857650982 |
| HCAG_05084 | Malate synthase | 395,86 | 4,095955267 |
|  |  |  |  |
| **Pentose-phosphate pathway** | |  |  |
| HCAG_04329 | Glucose 6 phosphate 1 dehydrogenase | 657,31 | * |
| HCAG_04762 | 6-phosphogluconolactonase | 715,43 | * |
| HCAG_05884 | 6-phosphogluconate dehydrogenase | 8396,72 | 1,733253039 |
|  |  |  |  |
| **Tricarboxylic-acid pathway** | |  |  |
| HCAG_03323 | Fumarate reductase flavoprotein subunit | 3104,04 | 2,033991215 |
| HCAG_04358 | Isocitrate dehydrogenase | 783,01 | 2,664456293 |
|  |  |  |  |
| **Respiration** |  |  |  |
| HCAG_06929 | NADH ubiquinone oxidoreductase | 321,8 | * |
|  |  |  |  |
| **Oxidation of fatty acids** |  |  |  |
| HCAG_08510 | Acyl-coa dehydrogenase | 2565,34 | * |
| HCAG_09978 | Acyl-coa dehydrogenase | 1447,54 | 1,599994191 |
| HCAG_02218 | Enoyl-coa hydratase isomerase family protein | 639,95 | * |
| HCAG_07725 | 3-hydroxybutyryl-coa dehydrogenase | 850,56 | 2,181472203 |
| HCAG_00780 | Acetyl-CoA Acyltransferase 1 | 524,39 | * |
| HCAG_00928 | Short chain dehydrogenase | 885,6 | * |
| **Energy conversion and regeneration** | |  |  |
| HCAG_02828 | ATP synthase subunit 4 | 1245,09 | * |
|  |  |  |  |
| **3. CELL CYCLE AND DNA PROCESSING** | |  |  |
| **Cell cycle** |  |  |  |
| HCAG_06189 | Mitogen activated protein kinase | 459,77 | * |
| HCAG_02915 | Zinc finger protein | 323,55 | * |
|  |  |  |  |
| **4. TRANSCRIPTION** |  |  |  |
| HCAG_08106 | Nmra family transcriptional regulator | 7405,14 | * |
| HCAG_04114 | RNA binding domain containing protein | 444,19 | * |
| HCAG_01318 | Transcription factor rfef putative | 5653,99 | * |
| HCAG_06738 | Trna cytosine-5-methyltransferase NCL1 | 577,73 | * |
| HCAG_02738 | U4 U6 X U5 tri snrnp complex subunit Prp4 family | 467,68 | * |
|  |  |  |  |
| **5. PROTEIN SYNTHESIS** |  |  |  |
| HCAG_05778 | Eukaryotic peptide chain release factor GTP binding subunit | 58,05 | * |
| HCAG_06198 | QDE2 protein | 1100,39 | * |
| HCAG_03055 | Ribosomal protein L23a | 3276,01 | 1,733253039 |
| HCAG_05192 | Ribosomal protein L31e | 5516,34 | 1,50681778 |
|  |  |  |  |
| **6. PROTEIN FATE AND DEGRADATION** | |  |  |
| HCAG_06935 | Aminopeptidase | 1055,9 | 2,293318702 |
| HCAG_00037 | Aspartic endopeptidase Pep2 | 3691,16 | * |
| HCAG_04297 | Aspartyl aminopeptidase | 369,75 | 1,954237353 |
| HCAG_04508 | Carboxypeptidase | 897,4 | * |
| HCAG_08666 | Carboxypeptidase | 460,05 | * |
| HCAG_01491 | Dihydrolipoamide branched chain transacylase E2 | 923,65 | * |
| HCAG_08942 | Dipeptidyl peptidase III | 792,39 | * |
| HCAG_04161 | Geranylgeranyl transferase type I beta subunit | 315,86 | * |
| HCAG_03543 | Glutamate carboxypeptidase | 2045,48 | 3,7061735 |
| HCAG_02754 | Oligopeptidase family protein | 618,16 | 1,973877746 |
| HCAG_06342 | Proteasome component | 403,49 | * |
| HCAG_04198 | Proteasome component | 884,35 | * |
| HCAG_05739 | Proteasome component | 500,32 | * |
| HCAG_05896 | Pyroglutamyl peptidase type I | 306,76 | * |
| HCAG_00635 | Serine proteinase | 510,85 | * |
| HCAG_03747 | Serine threonine protein kinase cbk1 | 58,05 | * |
|  |  |  |  |
| **7. PROTEIN WITH BINDING FUNCTION OR COFACTOR REQUIREMENT** | |  |  |
| HCAG_04426 | AIF like mitochondrial oxidoreductase | 1439,68 | * |
| HCAG_06523 | Curved DNA binding protein | 1003,08 | 1,665291179 |
| HCAG_03115 | Rheb small monomeric gtpase rhba | 734,07 | * |
|  |  |  |  |
| **8. CELLULAR TRANSPORT, TRANSPORT FACILITIES AND TRANSPORT ROUTES** | |  |  |
| HCAG_01041 | GTP binding protein | 512,8 | * |
| HCAG_06463 | K channel protein | 790,43 | * |
| HCAG_08974 | Nonspecific lipid transfer protein | 1143,19 | * |
|  |  |  |  |
| **9. CELL RESCUE, DEFENSE AND VIRULENCE** | |  |  |
| HCAG_11461 | Benzoate 4-monooxygenase cytochrome P450 | 522,17 | * |
| HCAG_08064 | Catalase B | 3577,16 | 1,840431425 |
| HCAG_05109 | Catalase isozyme A | 540,66 | * |
| HCAG_00642 | Copper zinc superoxide dismutase | 14672,71 | * |
| HCAG_06177 | Gamma glutamyltranspeptidase | 482,4 | * |
| HCAG_01006 | Glutathione S transferase | 1541,74 | * |
| HCAG_02530 | Hydroxyacylglutathione hydrolase | 1245,04 | * |
| HCAG_01543 | Superoxide dismutase, mitochondrial precursor | 465,38 | * |
|  |  |  |  |
| **10. CELL TYPE DIFFERENTIATION** | |  |  |
| HCAG_01014 | HET C domain containing protein HetC | 17,88 | * |
|  |  |  |  |
| **11. UNCLASSIFIED** |  |  |  |
| HCAG_02758 | Conserved hypothetical protein | 4447,83 | * |
| HCAG_03253 | Conserved hypothetical protein | 425,99 | * |
| HCAG_03288 | Conserved hypothetical protein | 675,05 | * |
| HCAG_03343 | Conserved hypothetical protein | 1323,68 | * |
| HCAG_04453 | Conserved hypothetical protein | 14119,72 | * |
| HCAG_04771 | Conserved hypothetical protein | 438,69 | * |
| HCAG_05185 | Conserved hypothetical protein | 3885,41 | 3,525421454 |
| HCAG_05480 | Conserved hypothetical protein | 511,27 | * |
| HCAG_05847 | Conserved hypothetical protein | 576,56 | * |
| HCAG_06192 | Conserved hypothetical protein | 869,93 | * |
| HCAG_06271 | Conserved hypothetical protein | 1589,68 | * |
| HCAG_06488 | Conserved hypothetical protein | 42,82 | * |
| HCAG_00447 | Conserved hypothetical protein | 499,49 | 1,858928051 |
| HCAG_00629 | Conserved hypothetical protein | 1648,19 | 19,10595464 |
| HCAG_00803 | Conserved hypothetical protein | 1142,25 | 3,254374032 |
| HCAG_00947 | Conserved hypothetical protein | 672 | * |
| HCAG_01170 | Conserved hypothetical protein | 634,1 | * |
| HCAG_01717 | Conserved hypothetical protein | 485,08 | * |
| HCAG_01952 | Conserved hypothetical protein | 695,19 | * |
| HCAG_02623 | Conserved hypothetical protein | 328,74 | * |
| HCAG_10123 | Conserved hypothetical protein | 825,45 | * |
| HCAG_10241 | Conserved hypothetical protein | 543,01 | * |
| HCAG_10554 | Conserved hypothetical protein | 42,3 | * |
| HCAG_10975 | Conserved hypothetical protein | 542,38 | * |
| HCAG_11214 | Conserved hypothetical protein | 1006,73 | * |
| HCAG_11469 | Conserved hypothetical protein | 445,5 | * |
| HCAG_08319 | Dehydratase | 620,43 | * |
| HCAG_07572 | DUF427 domain containing protein | 1422,72 | 3,421229602 |
| HCAG_01449 | DUF636 domain containing protein | 687,13 | * |
| HCAG_08082 | HHE domain containing protein | 832,52 | * |
| HCAG_08057 | Hypothetical protein | 1505,67 | * |
| HCAG_10802 | Hypothetical protein | 379,72 | * |
| HCAG_00223 | Predicted protein | 326,14 | * |
| HCAG_01997 | Predicted protein | 409,34 | * |
| HCAG_04526 | Predicted protein | 931,71 | * |
| HCAG_05550 | Predicted protein | 598,91 | * |
| HCAG_05686 | Predicted protein | 640,49 | * |
| HCAG_08909 | Predicted protein | 3575,83 | * |
| HCAG_09793 | Predicted protein | 389,58 | * |
| HCAG_10773 | Predicted protein | 1025,34 | * |
| HCAG_11145 | Predicted protein | 684,71 | * |
| HCAG_11645 | Predicted protein | 551,92 | * |
| HCAG_04397 | Tdcf protein | 21977,8 | 1,840431425 |

^a^ Identification of differentially regulated proteins from *Histoplasma* genome database (http://www.broadinstitute.org/annotation/genome/histoplasma_capsulatum/MultiHome.html) using the ProteinLynx Global Server vs. 2.4 (PLGS) (Waters Corporation, Manchester, UK).

^b^ Genes annotation from *Histoplasma* genome database or by homology from NCBI database (http://www.ncbi.nlm.nih.gov/).

^c^ Mycelium/Yeast means: The level of expression in mycelium divided by the level in the yeast cells.

^d^ Biological process of differentially expressed proteins from MIPS (http://mips.helmholtz-muenchen.de/funcatDB/) and Uniprot databases (http://www.uniprot.org/).

* Identified only in mycelium.
