# Supplementary material for: Comparative Proteomic Analysis of Histoplasma capsulatum Yeast and Mycelium Reveals Differential Metabolic Shifts and Cell Wall Remodeling Processes in the Different Morphotypes
Source: Front Microbiol. 2021 Jun 11;12:640931. doi: 10.3389/fmicb.2021.640931 (PMC8226243; doi:10.3389/fmicb.2021.640931)
Supplement: Supplementary Table 4 — Identified proteins from Histoplasma capsulatum up-regulated in yeast cells compared to mycelium. [file Table_4.DOCX]

**Supplementary Table 4: Identified proteins from *Histoplasma capsulatum* up-regulated in yeast cells compared to mycelium**

| **Accession number^a^** | **Protein Description^b^** | **Score** | **Ratio Yeast/Mycelium^c^** |
| --- | --- | --- | --- |
| **Functional categories^d^** | |  |  |
| **1. METABOLISM** | |  |  |
| **Amino acid metabolism** | |  |  |
| HCAG_08247 | 1,2-dihydroxy-3-keto-5-methylthiopentene dioxygenase | 1660,32 | * |
| HCAG_09984 | 2-isopropylmalate synthase | 751,82 | 2,1382762 |
| HCAG_07104 | 3-isopropylmalate dehydratase | 2673,21 | 1,537257535 |
| HCAG_06743 | Adenylosuccinate synthetase adb | 1665,4 | 1,750672504 |
| HCAG_10952 | Alanine glyoxylate aminotransferase | 414,98 | * |
| HCAG_05679 | Alanine transaminase | 879,78 | * |
| HCAG_05224 | Anthranilate synthase component I | 1765,98 | 1,954237353 |
| HCAG_00748 | Anthranilate synthase multifunctional enzyme | 568,1 | * |
| HCAG_00035 | Arginase | 4072,51 | 1,69893226 |
| HCAG_06102 | Aspartate aminotransferase | 2279,99 | 1,50681778 |
| HCAG_03751 | Aspartate aminotransferase | 464,49 | * |
| HCAG_08678 | Aspartate aminotransferase | 9312,15 | 1,993715528 |
| HCAG_08945 | Branched chain amino acid aminotransferase | 2667 | 1,768267039 |
| HCAG_00290 | Chorismate mutase | 1601,71 | 1,648721271 |
| HCAG_04326 | Cystathionine beta-synthase | 1879,67 | * |
| HCAG_00220 | Cysteine dioxygenase | 587,28 | * |
| HCAG_00386 | Cysteine synthase o acetylserine thiol lyase | 618,51 | * |
| HCAG_07464 | Gamma glutamyl phosphate reductase | 2513,15 | * |
| HCAG_05651 | Glutamate dehydrogenase | 1340,04 | * |
| HCAG_09757 | Glutamate synthase | 489,43 | * |
| HCAG_05070 | Glutamine synthetase | 463,23 | * |
| HCAG_07028 | Glutamine synthetase partial | 3066,2 | * |
| HCAG_02357 | Histidinol dehydrogenase | 575,85 | * |
| HCAG_06098 | Homocitrate synthase | 2328,53 | 2,534509196 |
| HCAG_00014 | Homoserine dehydrogenase | 740,91 | 1,822118844 |
| HCAG_08890 | Ketol acid reductoisomerase | 14496,76 | 2,316366916 |
| HCAG_09107 | L aminoadipate semialdehyde dehydrogenase large subunit | 502,64 | * |
| HCAG_05584 | Mitochondrial acetolactate synthase small subunit | 763,43 | 1,822118844 |
| HCAG_00029 | Mitochondrial methylglutaconyl coa hydratase | 381,98 | * |
| HCAG_05073 | N acetyl gamma glutamyl phosphate reductase | 561,61 | * |
| HCAG_03650 | NAD specific glutamate dehydrogenase | 956,08 | 2,159766213 |
| HCAG_07418 | Serine hydroxymethyltransferase | 3505,89 | 1,568312167 |
| HCAG_06253 | Threonine dehydratase | 369,45 | * |
|  |  |  |  |
| **Nitrogen, sulfur and selenium metabolism** | |  |  |
| HCAG_05304 | Acetamidase | 556,04 | * |
| HCAG_03008 | Sulfur metabolite repression control protein | 13211,48 | 1,69893226 |
|  |  |  |  |
| **Nucleotide metabolism** | |  |  |
| HCAG_05730 | 3,5-bisphosphate nucleotidase | 440,92 | * |
| HCAG_09104 | Adenylate kinase | 411,51 | * |
| HCAG_04776 | Deoxyuridine 5'-triphosphate nucleotidohydrolase | 9236,77 | 2,801065755 |
| HCAG_04496 | GMP synthase putative | 1405,55 | * |
| HCAG_02007 | Guanylate kinase | 753,74 | * |
| HCAG_04495 | Inosine 5 monophosphate dehydrogenase IMD2 | 517,96 | * |
| HCAG_03809 | Phosphoribosylaminoimidazole carboxylase | 462,86 | * |
| HCAG_06816 | Ribose phosphate pyrophosphokinase | 419,75 | * |
| HCAG_00005 | Rnapii degradation factor def1 | 1257,86 | 1,648721271 |
| HCAG_07700 | Xanthine phosphoribosyltransferase | 2181,6 | 1,632316236 |
|  |  |  |  |
| **C-compound and carbohydrate metabolism** | |  |  |
| HCAG_07210 | 1,3-beta-glucanosyltransferase | 424,86 | * |
| HCAG_07199 | Aldehyde reductase I | 5599,84 | 5,929856249 |
| HCAG_00318 | Alpha-1,3-mannosyltransferase | 99,96 | * |
| HCAG_02211 | Alpha glucosidase | 393,48 | * |
| HCAG_08290 | Gamma butyrobetaine dioxygenase | 499,41 | 1,87761057 |
| HCAG_05711 | GDP mannose pyrophosphorylase A | 2220,39 | * |
| HCAG_01828 | Glucan 1,3-beta-glucosidase | 394,39 | * |
| HCAG_08152 | Glucosamine-6-phosphate deaminase | 601,67 | * |
| HCAG_04088 | Glucosamine fructose-6-phosphate aminotransferase | 5594,67 | 3,935350714 |
| HCAG_06296 | Ketoreductase | 439,18 | * |
| HCAG_01552 | Mannose-1-phosphate guanylyltransferase | 1257,19 | 2,915379653 |
| HCAG_00064 | N acetylglucosamine phosphate mutase | 3928,04 | 1,716006899 |
| HCAG_08808 | Phosphoglucomutase | 1955,24 | 1,521961536 |
| HCAG_06641 | UDP galactopyranose mutase | 1000,06 | 1,682027618 |
| HCAG_07375 | Malic enzyme | 435,48 | * |
|  |  |  |  |
| **Lipid, fatty acid and isoprenoid metabolism** | |  |  |
| HCAG_02524 | 3-hydroxyisobutyryl-coa hydrolase | 3131,38 | 2,117000017 |
| HCAG_05042 | Acyl protein thioesterase | 428,01 | * |
| HCAG_03333 | Citrate lyase subunit beta | 529,8 | * |
| HCAG_03803 | Hydroxymethylglutaryl-coa lyase | 536,56 | * |
| HCAG_01797 | Sphingosine kinase | 391,09 | * |
| HCAG_07622 | Trans-2-enoyl-coa reductase | 1325,1 | * |
|  |  |  |  |
| **Metabolism of vitamins, cofactors, and prosthetic groups** | |  |  |
| HCAG_02020 | Delta aminolevulinic acid dehydratase | 386,38 | * |
| HCAG_03497 | Nicotinate nucleotide pyrophosphorylase | 4850,88 | 1,584073998 |
| HCAG_00907 | NUDIX hydrolase | 713,38 | * |
| HCAG_04775 | Pyridoxine kinase | 511,08 | * |
| HCAG_07304 | Thiamine phosphate pyrophosphorylase | 415,42 | * |
|  |  |  |  |
| **2. ENERGY** |  |  |  |
| **Glycolysis and gluconeogenesis** | |  |  |
| HCAG_08561 | Alcohol dehydrogenase | 11737,45 | 2,718281828 |
|  |  |  |  |
| **Tricarboxylic-acid pathway** | |  |  |
| HCAG_01535 | Alpha-ketoglutarate dehydrogenase | 755,11 | 1,599994191 |
| HCAG_05531 | Aconitate hydratase | 0 | * |
| HCAG_04093 | Isocitrate dehydrogenase subunit 1, mitochondrial precursor | 3417,77 | 2,054433269 |
| HCAG_04294 | Pyruvate dehydrogenase complex component Pdx1 | 1551,66 | * |
| HCAG_06317 | Succinate dehydrogenase | 1846,04 | 1,552707215 |
| HCAG_07697 | Succinyl coa ligase beta chain | 4023,32 | 1,733253039 |
|  |  |  |  |
| **Electron transport and membrane-associated energy conservation** | |  |  |
| HCAG_05938 | Cytochrome c | 2330,65 | 1,840431425 |
| HCAG_00943 | Ubiquinol cytochrome c reductase iron sulfur subunit | 411,23 | * |
|  |  |  |  |
| **Respiration** |  |  |  |
| HCAG_03721 | Alternative oxidase | 462,41 | * |
| HCAG_02171 | ATP synthase subunit g | 477,75 | * |
| HCAG_00437 | Cytochrome c oxidase chain VI | 3001,22 | 1,733253039 |
| HCAG_06996 | Processing enhancing protein | 1222,96 | * |
| HCAG_03209 | Vacuolar ATP synthase subunit E | 773,82 | * |
|  |  |  |  |
| **Oxidation of fatty acids** | |  |  |
| HCAG_09712 | Acetoacetyl coa synthase | 453,92 | * |
| HCAG_06958 | Long chain fatty acid coa ligase | 487,49 | * |
| HCAG_05071 | Short chain dehydrogenase | 4101,87 | 2,459603053 |
|  |  |  |  |
| **Energy conversion and regeneration** | |  |  |
| HCAG_08801 | F-type H+-transporting ATPase subunit H | 1279,51 | * |
|  |  |  |  |
| **3. CELL CYCLE AND DNA PROCESSING** | |  |  |
| **DNA processing** |  |  |  |
| HCAG_00486 | Chromatin assembly factor 1 subunit C | 451,54 | * |
| HCAG_03616 | HIRA interacting protein | 602,75 | * |
| HCAG_04914 | Histone H2A | 2238,71 | * |
| HCAG_04835 | Proliferating cell nuclear antigen | 8226,67 | 2,559981412 |
| HCAG_02026 | Replication factor A 1 | 514,98 | * |
| HCAG_01673 | Ruvb like helicase | 598,11 | * |
| HCAG_02975 | Ssdna binding protein | 5580,13 | 1,50681778 |
|  |  |  |  |
| **Cell cycle** |  |  |  |
| HCAG_00848 | Arp2/3 complex subunit | 1035,21 | * |
| HCAG_01653 | Cell division control protein | 505,93 | * |
| HCAG_01332 | Cell division cycle protein 37 | 904,87 | * |
| HCAG_07829 | Condensin | 17,75 | * |
| HCAG_05172 | Deubiquitination protection protein dph1 | 793,97 | * |
| HCAG_06605 | EB1 protein | 436,01 | * |
| HCAG_01730 | G2/M phase checkpoint control protein Sum2 | 559,35 | 9,393331377 |
| HCAG_03524 | Histone H2A | 3400,57 | 4,014849996 |
| HCAG_03525 | Histone H2B | 12100,49 | 2,915379653 |
| HCAG_01156 | M protein repeat protein | 417,54 | * |
| HCAG_07172 | Mob1 family protein | 363,51 | * |
| HCAG_09244 | Nuclear and cytoplasmic polyadenylated RNA binding protein pub1 | 1784,13 | * |
| HCAG_06735 | Nuclear segregation protein Bfr1 putative | 384,16 | * |
| HCAG_04938 | Spindle assembly checkpoint protein SLDB | 586,97 | * |
|  |  |  |  |
| **4. TRANSCRIPTION** | |  |  |
| HCAG_00922 | ATP dependent RNA helicase DBP7 | 400,66 | * |
| HCAG_05061 | ATP dependent RNA helicase SUB2 | 1580,8 | * |
| HCAG_06885 | Cap binding protein | 1033,23 | * |
| HCAG_00337 | Cleavage and polyadenylation specificity factor | 1749,74 | 1,954237353 |
| HCAG_02903 | Cyclin dependent protein kinase regulator | 473,44 | * |
| HCAG_10129 | Gtpase activating protein | 384,81 | * |
| HCAG_08774 | Histone chaperone ASF1 | 2063,15 | * |
| HCAG_06701 | Histone H3 | 1333,51 | 2,225540955 |
| HCAG_03885 | Histone H4 | 4116,49 | 2,534509196 |
| HCAG_03732 | KH domain RNA binding protein | 597,22 | * |
| HCAG_04339 | Multi bridging factor 1 putative | 2269,62 | * |
| HCAG_05099 | Nascent polypeptide associated complex subunit alpha | 8198,24 | 1,750672504 |
| HCAG_05051 | Nascent polypeptide associated complex subunit beta | 19731,5 | 1,568312167 |
| HCAG_05924 | Nuclear polyadenylated RNA-binding protein NAB2 | 459,65 | 2,316366916 |
| HCAG_02807 | Nucleic acid binding protein | 752,54 | * |
| HCAG_00461 | Pre mrna processing protein | 368,44 | * |
| HCAG_11185 | Pre mrna processing protein | 397,63 | * |
| HCAG_01906 | Ran gtpase activating protein | 582,08 | 1,584073998 |
| HCAG_08342 | RNA binding domain containing protein | 526,11 | * |
| HCAG_08343 | RNA binding domain-containing protein | 6623,62 | * |
| HCAG_00646 | RNA binding protein | 472,57 | * |
| HCAG_04410 | RNA binding protein | 3653,29 | 1,616074385 |
| HCAG_04664 | RNA recognition domain containing protein family protein | 783,29 | * |
| HCAG_00597 | Rnase III domain containing protein | 468,43 | * |
| HCAG_02082 | Ruvb like helicase | 372,06 | * |
| HCAG_08281 | Small nuclear ribonucleoprotein LSM2 | 929,19 | 1,915540783 |
| HCAG_01854 | Small nuclear ribonucleoprotein Lsm8 | 1380,64 | * |
| HCAG_03703 | Small nuclear ribonucleoprotein Sm D1 | 1060,44 | * |
| HCAG_01311 | Small nuclear ribonucleoprotein Sm D3 | 2948,13 | 2,247907992 |
| HCAG_01049 | Small nuclear ribonucleoprotein smf | 1747,81 | * |
| HCAG_02780 | Small nuclear ribonucleoprotein smg | 1828,66 | * |
| HCAG_00910 | Small nuclear ribonucleoprotein U2A | 977,19 | * |
| HCAG_00407 | Splicing factor 3A subunit 3 | 373,06 | * |
| HCAG_05023 | Splicing factor 3B | 390,98 | * |
| HCAG_06525 | Transcription factor Snf5p | 383,61 | * |
| HCAG_02611 | Transcription initiation factor TFIID subunit 14 | 1290,44 | 2,225540955 |
| HCAG_03203 | Transcriptional activator | 387,1 | * |
| HCAG_06889 | Transcriptional repressor TUP1 | 632,61 | 1,648721271 |
| HCAG_01469 | Trna ligase | 246,81 | * |
| HCAG_08192 | U1 snrnp-associated protein Usp107 | 1092,85 | 5,155169438 |
|  |  |  |  |
| **5. PROTEIN SYNTHESIS** | |  |  |
| HCAG_06035 | 30S ribosomal protein S7 | 381,75 | * |
| HCAG_05528 | 40S ribosomal protein s26 | 12600,74 | 3,525421454 |
| HCAG_01353 | 50S ribosomal protein L12 | 1667,79 | * |
| HCAG_05533 | 60S acidic ribosomal protein P1 | 23882 | 1,632316236 |
| HCAG_02703 | 60S acidic ribosomal protein P2 | 42675,45 | 1,50681778 |
| HCAG_07248 | 60S ribosomal protein L21 | 7476,61 | 1,716006899 |
| HCAG_08351 | 60S ribosomal protein L26 | 2791,51 | * |
| HCAG_04561 | 60S ribosomal protein L34 | 2552,83 | 1,537257535 |
| HCAG_04246 | 60S ribosomal protein L38 | 2652,12 | * |
| HCAG_01871 | Alanyl trna synthetase | 686,05 | * |
| HCAG_03866 | Arginyl trna synthetase | 373,08 | * |
| HCAG_04273 | ATP dependent RNA helicase eif4a | 13495,53 | 4,137120263 |
| HCAG_00406 | ATP dependent RNA helicase FAL1 | 1813,32 | 2,339646908 |
| HCAG_00616 | Cysteinyl trna synthetase | 772,71 | * |
| HCAG_03444 | Elongation factor Tu | 5007,09 | 3,387187831 |
| HCAG_01136 | Eukaryotic translation initiation factor 2 alpha subunit | 834,79 | * |
| HCAG_04206 | Eukaryotic translation initiation factor 2 gamma subunit | 435,67 | * |
| HCAG_08080 | Eukaryotic translation initiation factor 3 | 1359,85 | * |
| HCAG_03286 | Eukaryotic translation initiation factor 3 | 1202,74 | 1,521961536 |
| HCAG_08183 | Eukaryotic translation initiation factor 3 | 1160,72 | * |
| HCAG_08704 | Eukaryotic translation initiation factor 3 | 712,63 | * |
| HCAG_04356 | Eukaryotic translation initiation factor 3 subunit 2 | 1307,59 | 1,786038401 |
| HCAG_03353 | Eukaryotic translation initiation factor 3 subunit 3 | 492,92 | 3,287081395 |
| HCAG_00044 | Eukaryotic translation initiation factor 3 subunit 6 | 564,66 | * |
| HCAG_01956 | Eukaryotic translation initiation factor 3 subunit eifcf | 2711,24 | 1,803988368 |
| HCAG_05402 | Eukaryotic translation initiation factor 3 subunit K | 1041,4 | * |
| HCAG_01919 | Glutamyl trna synthetase | 385,5 | * |
| HCAG_03017 | Glycyl trna synthetase | 401,95 | * |
| HCAG_05229 | Leucyl trna synthetase | 363,77 | * |
| HCAG_00215 | Phenylalanyl trna synthetase | 467,77 | * |
| HCAG_07283 | Prolyl trna synthetase | 1386,4 | * |
| HCAG_08383 | Psi protein | 1264,93 | * |
| HCAG_03112 | Seryl trna synthetase | 1008,97 | * |
| HCAG_01178 | Translation initiation factor 3 | 5478,83 | * |
| HCAG_00267 | Translation initiation factor eif3 | 646,04 | * |
| HCAG_03569 | Translation initiation factor eif3a | 989,04 | * |
| HCAG_05882 | Tryptophanyl trna synthetase | 1077,57 | * |
| HCAG_06019 | Ubiquitin-60S ribosomal protein L40 fusion protein | 14005,84 | 1,768267039 |
|  |  |  |  |
| **6. PROTEIN FATE AND DEGRADATION** | |  |  |
| HCAG_07682 | 26S protease regulatory subunit | 366,3 | * |
| HCAG_02901 | 26S protease regulatory subunit | 376,4 | * |
| HCAG_00039 | 26S protease regulatory subunit | 784,94 | 1,599994191 |
| HCAG_00069 | 26S protease regulatory subunit | 1595,18 | 1,616074385 |
| HCAG_04181 | 26S protease regulatory subunit | 203,98 | * |
| HCAG_02442 | 26S proteasome non atpase regulatory subunit | 566,23 | * |
| HCAG_00247 | 26S proteasome regulatory subunit | 414,86 | * |
| HCAG_09105 | 26S proteasome regulatory subunit | 765,49 | * |
| HCAG_01847 | ADP ribosylation factor | 10909,51 | 1,665291179 |
| HCAG_01583 | Aha1 domain family | 5043,76 | * |
| HCAG_00603 | Aspartyl aminopeptidase | 471,08 | * |
| HCAG_04686 | ATP-dependent molecular chaperone HSC82 | 46362,8 | 6,619368586 |
| HCAG_00633 | Calcineurin catalytic subunit cnaa | 1043 | * |
| HCAG_08345 | Cell cycle control protein | 15367,29 | 3,560852494 |
| HCAG_04225 | Clathrin heavy chain | 1221,95 | * |
| HCAG_06960 | Cofilin | 3278,33 | 2,159766213 |
| HCAG_00176 | Dnaj and TPR domain containing protein | 381,38 | * |
| HCAG_04095 | DUF396 domain containing protein | 403,55 | * |
| HCAG_03748 | FK506 binding protein 1A | 811,45 | * |
| HCAG_06262 | GTP binding protein | 736,14 | * |
| HCAG_04252 | Metallopeptidase | 402,94 | * |
| HCAG_04853 | Mitochondrial protein import protein MAS5 | 2012,72 | * |
| HCAG_08291 | Monothiol glutaredoxin-4 | 525,83 | 2,611696417 |
| HCAG_00073 | Nuclear localization protein | 382,29 | * |
| HCAG_07345 | Peptidyl prolyl cis trans isomerase | 1724,36 | 2,013752683 |
| HCAG_08833 | Peptidyl prolyl cis trans isomerase | 24593,79 | 7,614086141 |
| HCAG_06091 | Polyubiquitin | 18008,54 | * |
| HCAG_01225 | Proteasome 26S subunit | 419,42 | * |
| HCAG_06228 | Proteasome 26S subunit | 429,86 | * |
| HCAG_04090 | Proteasome component | 380,31 | * |
| HCAG_07121 | Proteasome component | 711,87 | 2,316366916 |
| HCAG_08215 | Proteasome subunit alpha | 679,18 | * |
| HCAG_06316 | Proteasome-activating nucleotidase | 367,73 | * |
| HCAG_03630 | Protein disulfide-isomerase precursor | 9944,26 | 2,974274172 |
| HCAG_04821 | Protein phosphatases PP1 regulatory subunit sds22 | 466,1 | * |
| HCAG_08377 | Serine threonine phosphatase | 837,72 | * |
| HCAG_07282 | T-complex protein | 611,38 | * |
| HCAG_03305 | T-complex protein 1 subunit beta | 933,57 | * |
| HCAG_05524 | T-complex protein 1 subunit eta | 1141,7 | * |
| HCAG_05525 | T-complex protein 1 subunit eta | 673,23 | * |
| HCAG_04802 | Thioredoxin domain containing protein | 409,04 | * |
| HCAG_01487 | Ubiquitin conjugating enzyme | 1927,76 | * |
| HCAG_01770 | Ubiquitin like modifier SUMO | 1362,56 | * |
| HCAG_07567 | Zuotin | 635,21 | * |
|  |  |  |  |
| **7. PROTEIN WITH BINDING FUNCTION OR COFACTOR REQUIREMENT** | |  |  |
| HCAG_01880 | Actin binding protein | 451,47 | * |
| HCAG_02452 | Cell division cycle protein | 7188,92 | 1,632316236 |
| HCAG_01328 | Clathrin light chain | 451,46 | * |
| HCAG_02914 | Glycine rich protein | 12274,53 | 2,159766213 |
| HCAG_01447 | GTP binding protein | 1405,55 | 2,386910865 |
| HCAG_06941 | GTP binding protein | 2324,63 | 1,584073998 |
| HCAG_07144 | Myosin regulatory light chain cdc4 | 544,69 | * |
| HCAG_06026 | Polyadenylate binding protein | 2456,3 | 1,632316236 |
| HCAG_08950 | Progesterone binding protein | 2207,35 | * |
| HCAG_07540 | Ras like GTP binding protein RYL2 | 1904,32 | * |
| HCAG_05306 | RNP domain containing protein | 2937,09 | * |
| HCAG_04840 | Small COPII coat gtpase sar1 | 2706,11 | * |
| HCAG_05951 | Vacuolar ATP synthase subunit B | 640,7 | * |
|  |  |  |  |
| **8. REGULATION OF METABOLISM AND PROTEIN FUNCTION** | |  |  |
| HCAG_04544 | Peptide methionine sulfoxide reductase | 1045,99 | * |
|  |  |  |  |
| **9. CELLULAR TRANSPORT, TRANSPORT FACILITIES AND TRANSPORT ROUTES** | |  |  |
| HCAG_07845 | ABC transporter | 350,43 | * |
| HCAG_06610 | Arp2/3 complex subunit | 2577,83 | 1,521961536 |
| HCAG_00048 | Arsenical pump driving atpase | 1158,54 | * |
| HCAG_03176 | DUF207 domain containing protein | 400,74 | * |
| HCAG_04824 | Dynein light chain | 2005,2 | * |
| HCAG_05187 | GTP binding nuclear protein GSP1 Ran | 18463,48 | 2,509290432 |
| HCAG_07659 | GTP binding protein | 260,09 | * |
| HCAG_07669 | Meiotically up regulated gene 87 protein | 1,48 | * |
| HCAG_03107 | Membrane protein | 583,16 | * |
| HCAG_07102 | Nuclear pore complex subunit | 359,39 | * |
| HCAG_00557 | Phosphatidylinositol transfer protein SFH5 | 391,47 | * |
| HCAG_06977 | Plasma membrane atpase | 404,61 | * |
| HCAG_10603 | Snare sec23 | 476 | * |
| HCAG_05252 | Snare sec24 | 465,46 | * |
| HCAG_00404 | Vacuolar ATP synthase catalytic subunit A | 2001,44 | 1,50681778 |
|  |  |  |  |
| **10. CELLULAR COMMUNICATION / SIGNAL TRANSDUCTION MECHANISM** | |  |  |
| HCAG_01835 | CORD and CS domain containing protein | 2542,39 | * |
| HCAG_03194 | Rab geranylgeranyl transferase escort protein | 445,84 | * |
| HCAG_06999 | RAB1A | 1369,98 | 1,934792385 |
| HCAG_05787 | Ser/Thr protein phosphatase | 1007,09 | 2,293318702 |
|  |  |  |  |
| **11. CELL RESCUE, DEFENSE AND VIRULENCE** | |  |  |
| HCAG_00272 | Arsenite resistance protein Ars2 | 449,12 | * |
| HCAG_07098 | Cytochrome c peroxidase | 2459,3 | 2,637944535 |
| HCAG_08658 | Cytochrome c peroxidase | 468,25 | * |
| HCAG_09319 | Cytochrome c peroxidase | 9386,29 | 2,117000017 |
| HCAG_01785 | Heat shock protein | 496,42 | * |
| HCAG_04943 | Hsp10 like protein | 23411,41 | 10,27794075 |
| HCAG_06961 | Hsp60 like protein | 44302,76 | 4,953032542 |
| HCAG_01398 | Hsp70 like protein | 60301,22 | 2,691234498 |
| HCAG_05805 | Heat shock 70 kda protein C precursor | 7309,1 | 2,801065755 |
| HCAG_00783 | Hsp88 like protein | 8480,9 | 2,363160728 |
| HCAG_04111 | Heat shock protein 30 | 3722,74 | * |
| HCAG_08176 | Heat shock protein SSC1 | 16061,23 | 3,287081395 |
| HCAG_04471 | Heat shock protein STI1 | 6316,99 | 3,18993317 |
| HCAG_08451 | Mitogen activated protein kinase | 663,78 | * |
| HCAG_03646 | Stress protein p66 | 605,15 | * |
| HCAG_06210 | Thiol specific antioxidant | 23966,17 | 1,915540783 |
| HCAG_07019 | Thioredoxin reductase | 419,02 | * |
|  |  |  |  |
| **12. CELL FATE** |  |  |  |
| HCAG_04115 | Arp2/3 complex subunit | 408,79 | * |
|  |  |  |  |
| **13. BIOGENESIS OF CELLULAR COMPONENTS** | |  |  |
| HCAG_08210 | Actin | 10549,22 | 2,611696417 |
| HCAG_03174 | Arp2/3 complex 20 kda subunit | 1477,2 | 1,786038401 |
| HCAG_06375 | Tropomyosin | 6226,41 | 1,716006899 |
| HCAG_01781 | Tubulin beta chain | 2290,45 | 2,1382762 |
| HCAG_08288 | Tubulin subunit alpha 2 | 4748,61 | 2,944679677 |
|  |  |  |  |
| **14. CELL TYPE DIFFERENTIATION** | |  |  |
| HCAG_07461 | Fimbrin | 1092,39 | 1,954237353 |
| HCAG_02846 | Phosphoprotein phosphatase 2A regulatory subunit | 415,23 | * |
|  |  |  |  |
| **15. UNCLASSIFIED** | |  |  |
| HCAG_04173 | 3 family protein | 43525,16 | 2,075080647 |
| HCAG_08796 | Ankyrin repeat protein | 1078,52 | * |
| HCAG_07132 | BAR domain containing protein | 2751,71 | 1,616074385 |
| HCAG_06110 | Conserved hypothetical protein | 9042,95 | 2,459603053 |
| HCAG_07343 | Conserved hypothetical protein | 576,59 | * |
| HCAG_07444 | Conserved hypothetical protein | 1044,1 | * |
| HCAG_07970 | Conserved hypothetical protein | 840,68 | * |
| HCAG_11116 | Conserved hypothetical protein | 733,6 | * |
| HCAG_11677 | Conserved hypothetical protein | 396,04 | * |
| HCAG_04232 | Conserved hypothetical protein | 794,29 | * |
| HCAG_04347 | Conserved hypothetical protein | 519,33 | * |
| HCAG_05552 | Conserved hypothetical protein | 2250,57 | * |
| HCAG_07936 | Conserved hypothetical protein | 457,04 | * |
| HCAG_09818 | Conserved hypothetical protein | 452,05 | * |
| HCAG_00017 | Conserved hypothetical protein | 422,05 | * |
| HCAG_00022 | Conserved hypothetical protein | 486,38 | * |
| HCAG_00559 | Conserved hypothetical protein | 420,22 | * |
| HCAG_01019 | Conserved hypothetical protein | 817,19 | * |
| HCAG_01388 | Conserved hypothetical protein | 420,68 | * |
| HCAG_01715 | Conserved hypothetical protein | 408,98 | 3,254374032 |
| HCAG_02022 | Conserved hypothetical protein | 5522,88 | * |
| HCAG_02522 | Conserved hypothetical protein | 61,71 | * |
| HCAG_03731 | Conserved hypothetical protein | 491,51 | * |
| HCAG_04741 | Conserved hypothetical protein | 490,11 | * |
| HCAG_02296 | DUF1014 domain containing protein | 759,25 | * |
| HCAG_06069 | DUF1348 domain-containing protein | 460,42 | * |
| HCAG_02191 | GCY protein | 444,32 | * |
| HCAG_00122 | Hypothetical protein | 1040,8 | * |
| HCAG_10350 | Hypothetical protein | 518,1 | 2,054433269 |
| HCAG_03758 | Mitochondrial grpe | 3353,5 | 2,585709628 |
| HCAG_06409 | PCI domain containing protein | 798,52 | * |
| HCAG_01399 | Predicted protein | 808,25 | * |
| HCAG_03855 | Predicted protein | 612,51 | * |
| HCAG_04505 | Predicted protein | 583,96 | * |
| HCAG_09351 | Predicted protein | 432,51 | * |
| HCAG_09929 | Predicted protein | 396,61 | * |
| HCAG_09933 | Predicted protein | 497,14 | * |
| HCAG_10315 | Predicted protein | 929,07 | * |
| HCAG_10514 | Predicted protein | 368,29 | * |
| HCAG_10780 | Predicted protein | 4735,13 | 9,1157167 |
| HCAG_10781 | Predicted protein | 1761,95 | * |
| HCAG_10924 | Predicted protein | 685,09 | * |
| HCAG_11286 | Predicted protein | 547,18 | * |
| HCAG_03851 | RSC complex subunit | 724,68 | 1,934792385 |
| HCAG_04361 | Vid27 family protein | 516,05 | * |

^a^ Identification of differentially regulated proteins from *Histoplasma* genome database (http://www.broadinstitute.org/annotation/genome/histoplasma_capsulatum/MultiHome.html) using the ProteinLynx Global Server vs. 2.4 (PLGS) (Waters Corporation, Manchester, UK).

^b^ Genes annotation from *Histoplasma* genome database or by homology from NCBI database (http://www.ncbi.nlm.nih.gov/).

^c^ Yeast/Mycelium means: The level of expression in yeast cells divided by the level in the mycelia.

^d^ Biological process of differentially expressed proteins from MIPS (http://mips.helmholtz-muenchen.de/funcatDB/) and Uniprot databases (http://www.uniprot.org/).

* Identified only in yeast.
